# Eyes on the Prize: Tracking Electron Transfer in G‑Rich Duplex and Quadruplex DNA Using Enantiopure Ruthenium Polypyridyl Infrared Redox Probes

**DOI:** 10.1021/jacs.5c05736

**Published:** 2025-08-10

**Authors:** Mark Stitch, Martin Pižl, Niamh Lehane, Gregory M. Greetham, František Hartl, Michael Towrie, Susan J. Quinn

**Affiliations:** † School of Chemistry, 8797 University College Dublin, Dublin 4, Ireland; ‡ Department of Chemistry, 6816 University of Reading, Whiteknights, Reading RG6 6DX, U.K.; § Department of Inorganic Chemistry, University of Chemistry and Technology Prague, Technická 5, Prague 6 166 28, Czech Republic; ∥ Science and Technology Facilities Council, Rutherford Appleton Laboratory, Research Complex at Harwell, Didcot, Oxfordshire OX11 0QX, U.K.

## Abstract

Photosensitized damage by the mechanism of direct 1e^–^ transfer from a nucleobase to the metal complex is a complementary approach to type I and type II methods of photodynamic therapy. In this ultrafast spectroscopic study we report the ability of a nitrile infrared redox probe to report on the photo-oxidation of guanine-rich DNA, comprising persistent runs of guanine, by the dppz-10-CN containing complex [Ru­(TAP)_2_(dppz-10-CN)]^2+^ (**1** ^2+^), dppz-10-CN = 10-cyano-dipyrido­[3,2-a:2′,3′-c]­phenazine and TAP = 1,4,5,8-tetraazaphenanthrene. Our study reveals the ability of the enantiomers of **1** ^2+^ to photo-oxidize guanine in double-stranded and quadruplex DNA. Transient visible absorption reveals a high yield of the formation of the photoreduced metal complex due to photo-oxidation of guanine in the quadruplex-bound **1** ^2+^ systems, and that this is greater for the Λ enantiomer. Spectro-electrochemical and computational studies indicate the role of the dppz-10-CN as the preferred site of reduction, while time-resolved electronic absorption (TrA) spectroscopy highlights the impact of the enantiomers on the yield of photo-oxidation in the DNA systems. Notably, time-resolved infrared (TRIR) spectroscopy allows comprehensive tracking of the photo-oxidation dynamics by monitoring four key components, namely: (1) the transient band of the Ru/TAP-based lowest ^3^MLCT excited state, (2) bleach bands associated with DNA bases in close proximity to the excited state “site effect”, (3) the guanine radical cation band at ca. 1700 cm^–1^ and (4) the amplification of the red-shifted nitrile stretching vibration of the transient dppz-reduced complex. Together, these results allow detailed profiling of photoinduced electron transfer in DNA-bound ruthenium­(II) polypyridyl complex systems and highlight the potential of such redox probes. Overall, this study presents an important insight regarding the nature of charge transfer in a Hoogsteen-bound guanine quadruplex compared to Watson-Crick GC base pairings.

## Graphical abstract

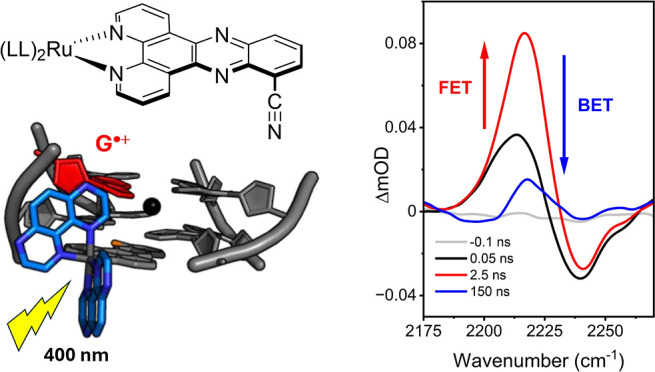

## Introduction

The ability to probe redox processes, including excited-state processes, in biological systems is essential to our understanding of enzyme function, photosynthetic centers, intrinsic photostability and the development of phototherapeutics. Photosensitized (PS) DNA damage by transition metal complexes is increasingly being considered for phototherapeutic applications.^1–6^ We are interested in developing metal polypyridyl probes that combine diagnostic and photodamaging properties and the potential to monitor their activity directly in biological environments. In addition to structural recognition, the intense charge-transfer (CT) character of transition metal polypyridyl complexes can be exploited to report on diverse nucleic acid structures through the light-switch effect,^7–11^ and to trigger DNA photo-oxidation through triplet-sensitized type I (generation of radical and reactive-oxygen species) and type II (singlet-oxygen generation)^1^ processes. Additionally, direct oxidation of guanine can be achieved by photoinduced one-electron transfer to the metal complex in what is believed to be a proton-coupled electron-transfer event (PCET).^12,13^

DNA photo-oxidation by direct electron transfer is enhanced for metal polypyridyl complexes containing extended dppz (dipyrido­[3,2-a:2′,3′-c]­phenazine) ligands, which can intercalate between the base pairs of DNA and allow strong binding to DNA.^14^ We have used time-resolved infrared (TRIR) spectroscopy to monitor single-electron photo-oxidation of guanine by [Ru­(TAP)_2_(dppz)]^2+^ (TAP = 1,4,5,8-tetraazaphenanthrene) in diverse DNA systems in solution and in crystals, and correlated the photophysical properties in solution to structural data obtained by X-ray diffraction studies on the DNA-bound systems.^12,14–17^ TRIR spectroscopy allows direct detection of the guanine radical cation (G^•+^) that absorbs at ca. 1700 cm^–1^.^18–20^ Such data is not readily obtainable by visible transient absorption (TrA) spectroscopy, as generally DNA transient species only absorb weakly at wavelengths shorter than 400 nm.^14^ Recently, we used TRIR spectroscopy to identify the role of a ligand-centered (^1^LC) state of the intercalated dppz ligand in the ultrafast direct oxidation of both guanine and adenine by a chromium­(III) complex [Cr­(TMP)_2_(dppz)]^3+^ (TMP = 3,4,7,8-tetramethyl-1,10-phenanthroline).^21^ We have extensively used the “site effect” to report on the site of photo-oxidation.^12,14,16,17^ This effect leads to diagnostic DNA-bleach bands in the 1600–1750 cm^–1^ region due to the perturbation of the nucleobases in the binding site of light-activated metal–polypyridyl probes and has been used to reveal the different binding interactions of the Λ and Δ enantiomers and the impact on the yield of the guanine photo-oxidation.^12,15,16,22,23^ The “site effect” has also been used to distinguish loop interactions from G-quartet stacking interactions for the *rac*-[Ru­(phen)_2_(dppz)]^2+^ light-switch complex bound to different structures of the human telomer sequence (**htel**) in solution.^24^

Infrared probes are useful reporters of the local environment in chemically important processes.^25,26^ Nitrile probes are particularly powerful due to the location of their vibration in the “transparent window” between 1800 and 2500 cm^–1^, which is well separated from the congested spectral regions of biological macromolecules such as proteins and DNA.^27^ This, combined with its sensitivity to its surroundings, has been exploited to characterize complex molecular environments^28,29^ including proteins,^30^ lipid membranes,^31^ and nucleic acids.^32^ The sensitivity of the ground-state nitrile vibration to the hydrogen-bonding environment in biological systems has previously been highlighted in the work of Boxer and co-workers,^29,33,34^ and the work of Bagchi and co-workers.^35^ Notably, a significant enhancement of the nitrile vibrational absorption in the infrared (IR) spectrum has been observed for the excited states of aromatic nitriles^36^ and CN-substituted bipyridyl complexes of ruthenium­(II).^37,38^ In a key paper, McCusker and co-workers demonstrated the ability of a CN-substituted bipyridyl ligand to provide an IR tag that was spectrally well-isolated, coupled into the MLCT excited-state manifold, and readily identifiable in terms of its role in the excited state (e.g., either as a spectator ligand or housing the ligand-based electron of the MLCT state).^37,39^ In the first study of its kind we recently reported the significant enhancement of the nitrile stretching vibration in the MLCT excited state of the DNA-intercalating complex [Ru­(phen)_2_(dppz-11,12-CN)]^2+^ (phen = 1,10-phenanthroline) formed upon 400 nm excitation.^40^ The linear red shift of the intense nitrile ν­(C≡N) transient absorption at 2232 cm^–1^ in response to hydrogen bonding, combined with the change in the excited state lifetime, was used to report on the binding site environment in quadruplex DNA. Molecular-dynamics simulations combined with binding-energy calculations have identified the most favorable binding site for each system, in excellent agreement with the observed TRIR solution study.^40^ The study highlighted the power of combining the environmental sensitivity of an infrared (IR) probe in its excited state with the TRIR DNA “site effect” to gain important information about the binding site of photoactive agents and points to the potential of such amplified IR probes as sensitive reporters of biological environments. The enhancement of a nitrile stretching vibration was also recently reported by Hartl and co-workers for electrochemically reduced [Ru­(TAP)_2_(PP)]^2+^ complexes, where PP = dppz-11-CN or dppz-11,12-CN. In that study, the nitrile-containing PP ligand (mainly its phenazine moiety) was observed to be the site of the initial 1e^–^ reduction.^41^

DNA binding is influenced by the presence of substituents in the dppz-11 and -12 positions,^42^ and the presence of a terminal nitrile in [Ru­(TAP)_2_(dppz-11-CN)]^2+^ is found to enhance stacking interactions with B-DNA.^43^ While studies have reported that substitution in the dppz-10 position can greatly impact DNA binding interactions. For example, the inclusion of a flexible 10-(2-(piperidin-1-yl)­ethoxy (10-pe) chain in [Ru­(bpy)_2_(dppz-10-pe)]^2+^ showed improved quadruplex stability and telomerase inhibition.^44^ Structural studies show that substitution by a methyl group in this position in [Ru­(TAP)_2_(dppz-10-Me)]^2+^ directs the group exclusively into the major groove and toward the pyrimidine side of the intercalated step in double-stranded oligomer.^42^ In addition, NH_2_ substitution in the 10-position in a dppz-related pteridinyl–phenanthroline complex has been shown to stabilize the double-stranded calf thymus DNA.^45^

In the ultrafast spectroscopic study below we aim to examine the ability of a nitrile IR redox probe to report on the photo-oxidation of guanine-rich DNA, comprising persistent runs of guanine, by the dppz-10-CN containing complex [Ru­(TAP)_2_(dppz-10-CN)]^2+^ (**1** ^2+^), see Figure 1. Here, the 10-position was chosen to report from an imbedded location within the DNA for the chirally resolved enantiomers (Δ-**1** ^2+^ and Λ-**1** ^2+^). The DNA systems chosen for the study are the single-stranded control polyG sequence, duplex d­(GGGGGCCCCC)_2_ and the quadruplex forming human telomer sequence 5′-TTGGG­(TTAGGG)_3_A-3′. These sequences are expected to be particularly susceptible to photo-oxidation, as guanine is the most readily oxidized base and its susceptibility to photo-oxidation increases for runs of guanine bases, where this susceptibility increases in the order 5′-GGG-3′ > 5′-GG-3′ > 5′-G-3′.^46,47^ Quadruplex DNA presents an important therapeutic target, as sequences capable of forming these structures are over-represented in oncogenes.^48^ The study aims to provide a comprehensive tracking of the photo-oxidation dynamics by monitoring four key components, namely: (1) the transient band of the Ru→TAP-based ^3^MLCT excited state, (2) bleach bands associated with the perturbed DNA bases in close proximity to the excited-state “site effect”, (3) the guanine radical cation band at ca. 1700 cm^–1^ and (4) the red shift in frequency and amplification of the nitrile stretching vibration of the dppz-reduced complex.

**Figure 1:**
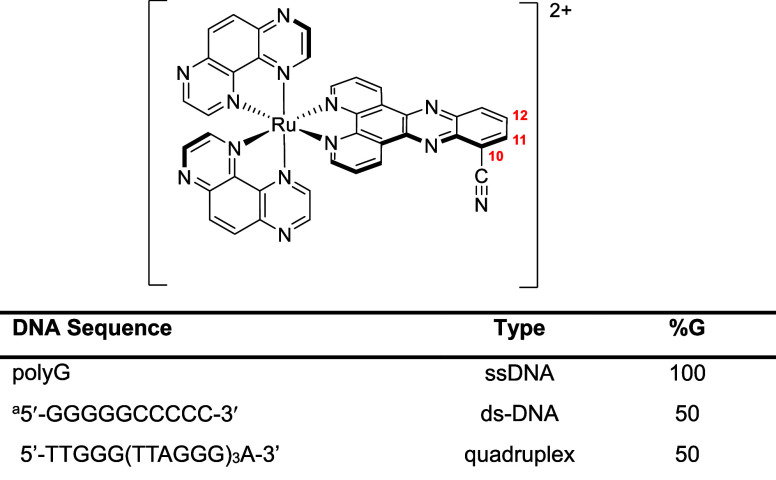
Structure of the [Ru­(TAP)_2_(dppz-10-CN)]^2+^ complex (1^2+^) together with double stranded DNA systems (^a^self-complementary), with the guanine composition varying from 50% to 100%.

## Results

### Synthesis and Spectroscopic Characterization of **1** ^2+^

The new complex [Ru­(TAP)_2_(dppz-10-CN)]­Cl_2_, [**1** ^2+^]­Cl_2_ was prepared via a two-step procedure (Scheme S1). Briefly, [Ru­(cyclo-octa-1,5-diene)_2_]­Cl_2_ was combined with two equivalents of TAP in degassed DMF and irradiated in a microwave reactor (at 433 K for 45 min) to form the dichlorido precursor complex [Ru­(TAP)_2_Cl_2_]. Further microwave reaction with a stoichiometric amount of dppz-10-CN was followed by purification via CM- sephadex C-25 column (salt gradient 10^–2^ M to 10^–1^ M) to form **1** ^2+^ as a red precipitate after desalting and solvent removal; this was confirmed by mass spectrometry, NMR and IR spectroscopies (Figures S1–S3). The enantiomers of **1** ^2+^ were then resolved by passing through a C25-sephadex column eluted with a (−)-*O*,*O*′-dibenzoyl-l-tartrate mobile phase. In all aqueous solutions **1** ^2+^ refers to the complex in its chloride salt form.

The UV–vis absorption spectrum of **1** ^2+^ recorded in an aqueous solution, is shown in Figure 2a. The intense transitions observed between 250 and 300 nm are assigned to singlet ligand-centered (^1^LC, π → π*) excitations associated with the TAP and dppz moieties. Additional ^1^LC transitions associated with the dppz ligand are observed at 377 nm. The region between 370 and 520 nm contains overlapping singlet metal-to-ligand charge-transfer (^1^MLCT) transitions from the Ru center to both TAP and dppz-10-CN ligands. The circular dichroism spectra for the Δ and Λ stereoisomers of **1** ^2+^ show opposite (but equal) Cotton effects with characteristic couplets observed for the ^1^LC and ^1^MLCT transitions (Figure S4). Complex **1** ^2+^ is highly emissive in aerated aqueous solutions; visible-light excitation results in broad phosphorescence at 630 nm, with a lifetime of 826 ns. The phosphorescence at 630 nm is characteristic of the TAP-based ^3^MLCT emission observed for the reference [Ru­(TAP)_2_(dppz)]­Cl_2_ complex and calculated hereinafter for **1** ^2+^ as the lowest-energy spin-triplet excited state (Table S4).^13^

**Figure 2:**
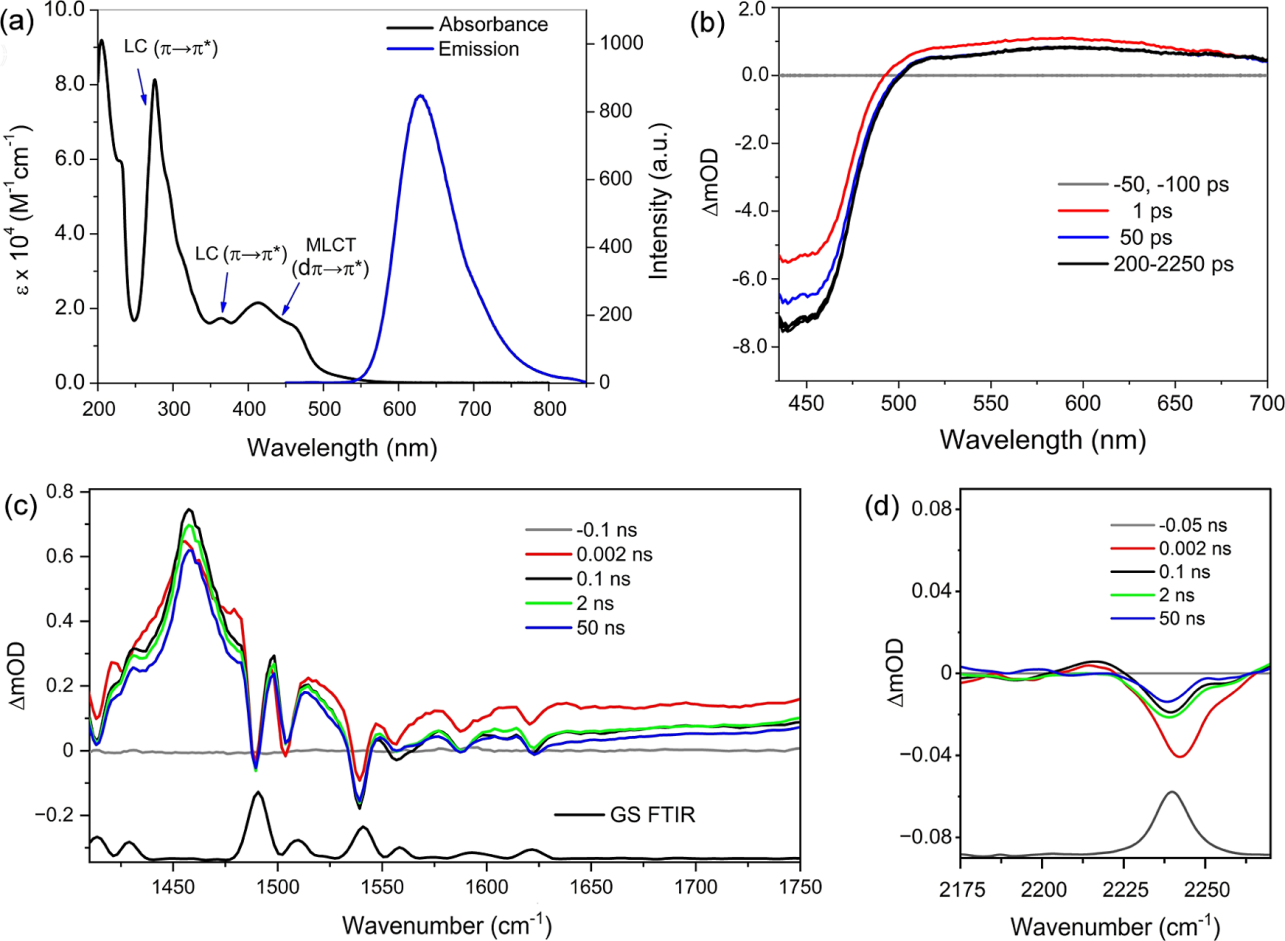
(a) UV–vis absorption and emission spectra of 11 μM **1** ^2+^ in an aqueous solution. (b) TrA of **1** ^2+^ in D_2_O upon 400 nm excitation. TRIR difference absorbance spectra of 0.8 mM **1** ^2+^ in 50 mM phosphate, pH 7, in (c) D_2_O and (d) H_2_O. Note that in (d) the spectra were vertically translated to account for the baseline offset (λ_exc_ = 400 nm, 2 kHz, 150 fs).

Photoexcitation into the ^1^MLCT absorption band of **1** ^2+^ at 400 nm is expected to optically populate both the TAP- and dppz-10-CN-based ^1^MLCT excited states, which subsequently populate the lowest ^3^MLCT state. The ps-TrA spectrum of *rac*-**1** ^2+^ in D_2_O shows the presence of a large negative “bleach” signal below 500 nm, corresponding to the removal of the ground state, see Figure 2b. This is accompanied by the appearance of a broad transient at ca. 600 nm assigned to the lowest TAP-based ^3^MLCT state ^3^[Ru^III^(TAP^•–^)­(TAP)­(dppz-10-CN]^2+^.^20,49^ This is supported by similarity of the TrA spectrum of **1** ^2+^ recorded at 50 ps and that of [Ru^III^(TAP^•–^)­(TAP)­(dppz)]^2+^ (ref. 50), see Figure S5a. The intensity of the transient band is observed to slightly decrease at early times with a lifetime of ca. 33 ± 3 ps (Figure S5b), which is accompanied by a "grow-in" of the bleach band signal. These changes are attributed to the formation of an intermediate species which likely arises due to exchange between ^3^MLCT excited states located on the TAP or at proximal (phen) or distal (phenazine) positions on the dppz ligand, this has previously been observed with related complexes.^20^ While time-resolved Raman experiments have attributed the increasing magnitude of the ground state bleach after the laser flash to progression through an absorbing intermediate excited state,^51^ which in the case of **1** ^2+^ may have a different ^3^MLCT character. The role of intersystem crossing (ISC) can be ruled out here, as this is expected to occur on the subpicosecond time scale for complexes of this type.^52^ The subsequent spectrum, recorded between 100 and 2400 ps, shows little change in the transient absorption, which is expected for this luminescent complex.

The ground-state FTIR spectrum recorded in the D_2_O solution shows structured bands from 1600 cm^–1^ down to 1300 cm^–1^ due to the ring-based vibrations on the polypyridyl ligands (Figure S3, Supporting Information). The ps-to-ns TRIR spectra recorded upon the 400 nm excitation of **1** ^2+^ are shown in Figure 2c. Between 1270 and 1545 cm^–1^, the spectra are dominated by several bleach and transient bands, corresponding to changes in the vibrational excited-state modes of **1** ^2+^. The well-resolved bleach bands at 1490, 1504 and 1540 cm^–1^ correspond to the depletion of the ground state of the complex. A particularly strong and broad transient band is observed at 1458 cm^–1^, arising from the TAP-based ^3^MLCT excited state.^12,50^ This transient absorption is observed to increase in intensity with a lifetime of ca. 28 ps (Figure S6). As observed for the TrA spectra, there is no decay of the intensity of the 1458 cm^–1^ band over 2 ns but it is observed to decay over 5 μs. Notably, there are no transient or bleach IR bands of significant intensity in the characteristic DNA region between 1625 and 1750 cm^–1^.

The FTIR spectrum of the chloride salt of **1** ^2+^ recorded in H_2_O shows a weak ν­(*C*≡*N*) vibration at 2240 cm^–1^ (Figure 2d), which is significantly separated from the vibrations associated with the crowded DNA window (1625 cm^–1^ and 1750 cm^–1^). The TRIR spectrum of the photoexcited complex shows a characteristic bleach at 2240 cm^–1^ corresponding to the loss of this ground-state vibration and the appearance of a very weak transient at ca. 2215 cm^–1^ (Figure 2d). The 25 cm^–1^ bathochromic shift of the nitrile stretching frequency indicates weakening of the CN bond in the MLCT excited state. Kinetic analysis reveals a biexponential recovery of the bleach intensity with completion of the faster process within 100 ps (Figure S6). This is similar to the behavior observed for the 1458 cm^–1^ transient and the 600 nm transient in the TrA spectra, reflecting the formation of the Ru/TAP-based ^3^MLCT. The subsequent recovery of the GS occurs over longer times (note at 50 ns the ground state has not fully recovered).

#### Electrochemistry

In dichloromethane/TBAH at ambient temperature, **1** ^2+^ undergoes four consecutive reversible reductions (R1–R4) at −1.14 V, −1.46 V, and −1.93 V vs ferrocenium/ferrocene. The first step corresponds with poorly resolved two 1e^–^ reductions (R1 + R2) associated with the remote phenazine-CN part of the dppz-10-CN ligand and the TAP ligands (Table S1 and Figure S7a). A similar situation was encountered for the related complex^41^ [Ru­(TAP)­(dppz-11-CN)]^2+^. Lowering the electrolyte temperature to 195 K resulted in a slight separation of R1 (−1.10 V) and R2 (−1.19 V) (Figure S7b). The reduction waves of **1** ^2+^ were also recorded in butyronitrile/TBAH at various working disc electrode materials (Figure S8a–c). As expected, the stepwise 1e^–^ reductions of the free ligand dppz-10-CN to the corresponding radical anion and dianion are negatively shifted to −1.46 V and −2.21 V in dichloromethane/TBAH (Figure S9), with only the first step being fully reversible at ambient temperature.

#### UV–vis and IR Spectroelectrochemistry

In situ spectroelectrochemical (SEC) measurements of **1** ^2+^ within an OTTLE cell, carried out in dichloromethane/TBAH, with outcomes summarized in Table 1, have revealed distinct changes in the visible absorption and IR spectra, associated with the sequential electrochemical reductions at R1–R3. In the case of the electronic absorption spectrum, the initial unresolved 2e^–^ reduction at R1–R2 is distinguished by the decreasing absorption of the parent complex at 276 and 414 nm, accompanied by the growth of new absorption bands at 450–600 nm (with a maximum at 472 nm) associated with the reduction of the TAP ligand to the corresponding radical anion, while the parallel reduction localized on the dppz-10-CN ligand in **1** ^2+^ results in two absorption bands between 600 and 700 nm (Figure 3a). The visible absorption spectrum of the 2e^–^-reduced **1** ^0^ is markedly different from that observed previously for the TAP-reduced [Ru­(TAP)_2_(dppz)]^*n*^ (*n* = 1+, 0),^50^ with the notable electronic absorption of the radical anion of dppz-10-CN below 600 nm, in line with the reference [Ru­(TAP)_2_(dppz-11-CN)]^2+^.^41^ The subsequent TAP-localized 1e^–^ reduction at R3 to form **1** ^**–**^ results in a modest increase in the absorption at about 500 nm (Figure S10). Similar observations were made for **1** ^*n*^ (*n* = 2+, 0, 1−) when the UV–vis SEC measurements were instead performed in butyronitrile/TBAH (Figure S11), despite the complicated CV responses in this electrolyte. Notably, the characteristic absorbance at 600–750 nm was also observed for the 1e^–^ reduction of the dppz-10-CN ligand to [dppz-10-CN]^•–^ (Figure S12a).

**Table 1:** IR SEC and UV–vis SEC Data for Dppz-10-CN, **1** ^2+^ and Their Reduced Forms

compound	*n*	solvent	ν(C≡N)/cm^–1^	λ_max_/nm
dppz-10-CN^*n*^	0	DCM	2233	263, 294, 305, 367, 386
	1^–^		2198	238, 320, 395, 454, 491, 700, 900
[Ru(TAP)_2_(dppz-10-CN)]^*n*^	2^+^	DCM	2235	276, 364, 414, 450sh
		PrCN		275, 365, 415, 450sh
	0	DCM	2205	278, 343, 472, 628, 685
		PrCN		275, 338, 471, 626, 680
	1^–^	DCM	2202	279, 342, 484, 629, 689
		PrCN		275, 341, 488, 630, 689

**Figure 3:**
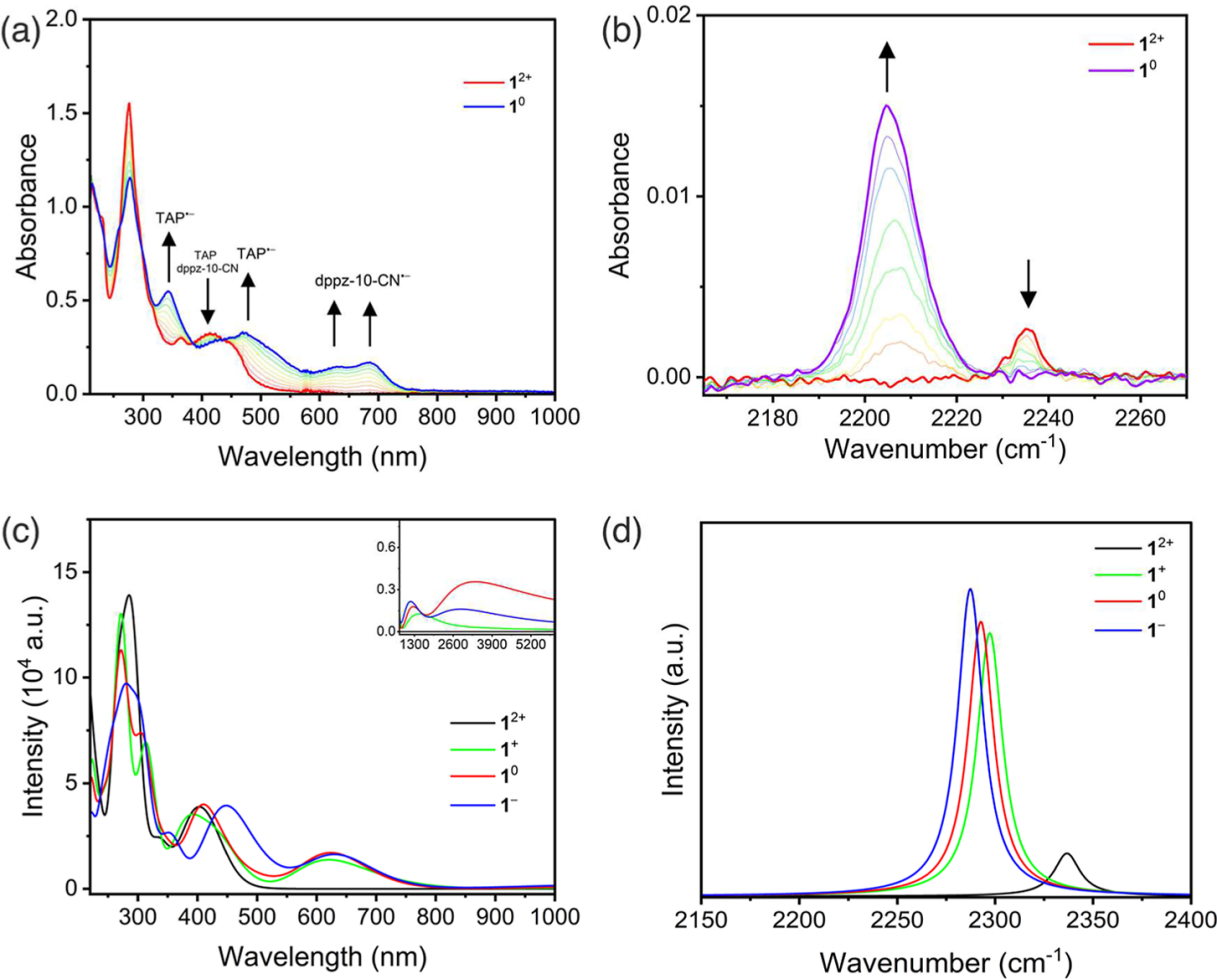
(a) UV–vis SEC of 0.75 mM **1** ^2+^ in DCM/0.1 M TBAH, and (b) IR SEC of 1.5 mM **1^2+^** in DCM/0.2 M TBAH. Conditions: an OTTLE cell with a Pt minigrid working electrode, *T* = 293 K. DFT-calculated (c) UV-vis-NIR electronic absorption spectra and (d) IR spectra of **1** ^2+^ (black line), **1** ^+^ (green line), **1** ^0^ (red line) and **1** ^–^ (blue line).

The corresponding IR SEC monitoring of **1** ^2+^ during the reduction steps R1–R3 were conducted in parallel with the UV–vis SEC experiment in dichloromethane/TBAH described above. The initial 2e^–^ reduction (R1 + R2) of **1** ^2+^ to form **1** ^0^ resulted in dramatic changes in the IR ν­(*C*≡*N*) region. The parent ν­(*C*≡*N*) absorption at 2235 cm^–1^ disappeared and a new ν­(*C*≡*N*) absorption band at 2205 cm^–1^ grew up with a markedly higher intensity, enhanced by a factor of 6 (Figure 3b). The reduction was also accompanied by changes in the fingerprint IR region between 1300 and 1500 cm^–1^ (Figure S13a). Further reduction at R3 to form **1** ^**–**^ resulted in a slight red shift of ν­(*C*≡*N*) (Table 1) and increased intensity of the nitrile vibration (Figure S14b). The spectral changes observed for the reduction of free dppz-10-CN mirrored those observed for the ligand in **1** ^2+^ at R1/R2 (Table 1 and Figure S12b), revealing that the nitrile vibration is not significantly affected by the coordination of dppz at the Ru­(II) center.

#### DFT and TDDFT Calculations

The assignment of the experimental time-resolved laser spectroscopic and spectroelectrochemical data was facilitated by DFT calculations unravelling the nature of the frontier orbitals in **1** ^2+^ and the distribution of spin densities in the reduced species. DFT and TDDFT methods were used to simulate the electronic and vibrational spectra of free dppz-10-CN and **1** ^2+^, and their reduced forms. The calculated electronic absorption spectra obtained for dppz-10-CN and [dppz-10-CN]^•–^ are in good agreement with the recorded experimental spectra, also showing the appearance of the diagnostically important intraligand absorption between 600 and 750 nm upon the reduction (Figure S15a, Table S3 and Figure S18). In the singly reduced **1** ^+^, this spectral region is dominated by ligand-to-ligand charge transfer­(LLCT) ([dppz-10-CN]^•–^→TAP, α-HOSO→α-LUSO+6) and intra-ligand charge-transfer (ILCT) ([dppz-CN]^•–^-based, α-HOSO→α-LUSO+8) transitions (Table S6 and Figure S27). The strong enhancement of the intensity of the red-shifted IR ν­(*C*≡*N*) absorption band intensity of [dppz-10-CN]^•–^ has also been reproduced (Figure S15b). The calculated spin density distribution in reduced **1** ^*n*^ (*n* = 1+, 0, 1−) is illustrated in Figure S19. The HOMO and LUMO of **1** ^2+^ are largely localized on the ruthenium­(II) center and dppz-10-CN, respectively, with the closely lying LUMO+1 located on both TAP ligands (Figure S20). The LUMO of **1** ^2+^ resides on the remote phenazine moiety, hence explaining the strong impact of the initial **1** ^2+^ reduction on the vibration of the nitrile substituent, reported above from the IR SEC study. The DFT-calculated electronic absorption (Figure S24) and IR spectra (Figure S21) of **1** ^2+^ and its reduced forms **1** ^*n*^ (*n* = 1+, 0, 1−) are found to be in good agreement with the experimental SEC data (Table 1 and Figure 3c,d). Notably, the calculated IR spectrum of **1** ^+^ confirmed the intensity enhancement of ν­(*C*≡*N*) by a factor of ca. 6 compared to **1** ^2+^, and the large decrease in the ν­(*C*≡*N*) wavenumber by 40 cm^–1^ (from 2337 to 2297 cm^–1^ (not scaled)). For 2e^–^-reduced **1** ^0^, there is an additional red shift of ν­(*C*≡*N*) by 5 cm^–1^.

Apart from the ground states of **1** ^2+^ and its reduced forms, also the low-lying excited states of **1** ^2+^ were calculated with DFT. This revealed that the Ru-TAP-localized ^3^MLCT state is the lowest ^3^MLCT state obtained by ISC from two optically populated ^1^MLCT states, viz. Ru→TAP and Ru→phen­(dppz-10-CN), upon the 400 nm excitation (Table S4). The calculated spectrum (Figure S23) is consistent with the TrA spectrum shown in Figure 2a. The calculations also predict that the Ru-TAP-localized ^3^MLCT state of **1** ^2+^ exhibits a larger ν­(*C*≡*N*) wavenumber compared to the ground state of the complex, and the ν­(*C*≡*N*) intensity becomes much less affected compared to the electrochemical 1e^–^ reduction initially residing on dppz-10-CN, as revealed by the calculated LUMO character. The calculated difference spectrum is in good agreement with that observed in the TRIR experiment (Figure S25).

#### Steady-State Spectroscopic Study of Binding of **1** ^2+^ to DNA Systems

The luminescence of [**1** ^2+^]­Cl_2_ (Figure 2a) is found to be quenched in the presence of guanine monophosphate (GMP), see Figure S31, which is similar to what is observed for the reference [Ru­(TAP)_2_(dppz)]­Cl_2_ complex.^13^ Quenching is also observed in the presence of an increasing concentration of polyguanylic acid (polyG), which is accompanied by a significant decrease in the absorbance of the dppz-based π→π* transitions at 364 nm and the ^1^MLCT band at 415 nm (Figure S32). Similar changes in the UV–vis absorption spectra are observed for the enantiomers of **1** ^2+^in the presence of the self-complementary sequence **d­(G**_**5**_ **C**_**5**_ **)**, with the appearance of a well-defined isosbestic point at 438 nm, see Figures 4a and S33. These spectral changes are characteristic of the intercalation of the dppz-10-CN ligand between the bases. However, more modest changes were observed in the UV–vis absorption spectra of **1** ^2+^ in the presence of a steadily more concentrated K^+^ stabilized hybrid-1 quadruplex **hTel** formed from the telomere DNA sequence (Figure S35). Though, notably, in the case of the quadruplex, the hypochromism was slightly greater for Δ-**1** ^2+^ than Λ-**1** ^2+^.

**Figure 4:**
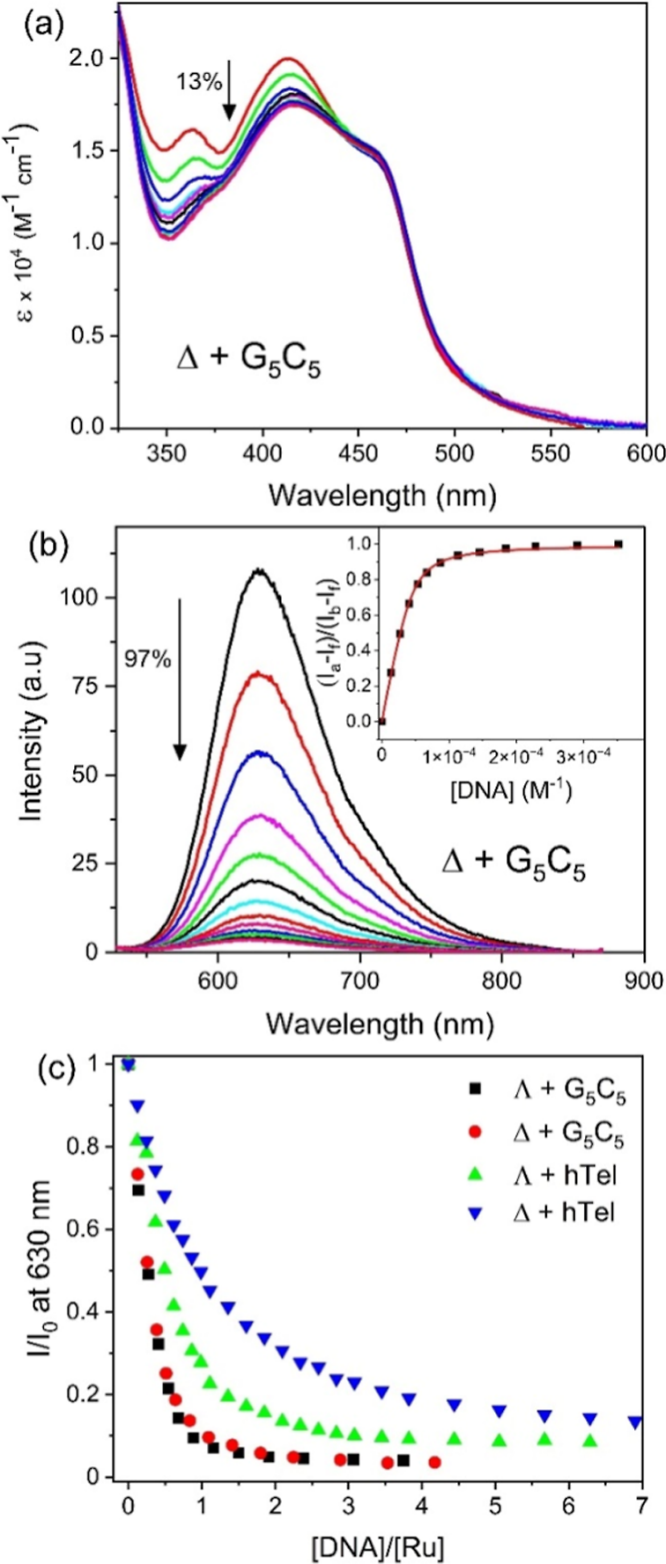
(a) UV–vis absorption spectra and (b) phosphorescence of 21.4 μM Δ-**1** ^2+^ in the presence of increasing concentrations of d­(G_5_C_5_)_2_ (0 → 91 μM duplex) in the presence of 50 mM potassium phosphate buffer, pH 7. (c) Relative changes in the phosphorescence intensity at 630 nm for Λ-**1** ^2+^ and Δ-**1** ^2+^ in the presence of the different DNA sequences (λ_exc_ = 450 nm).

Almost complete quenching of the luminescence at 630 nm was observed for both enantiomers Λ-**1** ^2+^ (96%) and Δ-**1** ^2+^ (97%) in the presence of the double-stranded **d­(G**_**5**_ **C**_**5**_ **)**_**2**_ DNA, which is expected, as each binding site locates the complex next to a guanine base (Figures 4b and S33). Binding constants derived from the luminescence data, using the Bard equation,^53^ are given in Table 2. In the case of **d­(G**_**5**_ **C**_**5**_ **)**_**2**_, the binding constant for Λ-**1** ^2+^ was found to be slightly greater than that for Δ-**1** ^2+^. Incomplete quenching (86%) was observed for both enantiomers when the titrations were repeated in the presence of the mixed-base natural DNA from salmon testes (**st-DNA**) with a 42% GC content (Figure S34). Quenching of the luminescence was also observed in the presence of **hTel** DNA, viz. 93% for Λ-**1** ^2+^ and 89% for Δ-**1** ^2+^. However, the luminescence-derived binding constants indicate a slightly weaker affinity for the quadruplex structure (Figure S35). These results are summarized in Table 2. Similar differences in the binding affinity have been observed in previous studies on related complexes.^40,54^ A comparison of the quenching behavior for the different double-strand and quadruplex systems is shown in Figure 4c. Interestingly, while only a slight difference is seen between the enantiomers in the presence of **d­(G**_**5**_ **C**_**5**_ **)**_**2**_, they are found to display different quenching in the presence of the hybrid quadruplex structure formed by the **hTel** sequence stabilized by potassium cations, see Figure 4c.

**Table 2:** DNA-Binding Constants (*K*_b_) and Binding-Site Size Determined for Enantiomers of **1** ^2+^, Using the Bard-Fitting of Luminescence at 630 nm, for DNA-Titrated Systems in the Presence of 50 mM Potassium Phosphate Buffer at pH 7

System	Binding constant *K*_b_ (M^–1^)/binding-site size
Δ-**1** ^2+^/**st**	2.4(±0.8) × 10^6^/2.5(±0.1)
Λ-**1** ^2+^/**st**	2.2(±0.5) × 10^6^/1.1(±0.1)
Δ-**1** ^2+^/**d(G_5_C_5_)_2_**	7.8(±0.8) × 10^5^/2.1(±0.1)
Λ-**1** ^2+^/**d(G_5_C_5_)_2_**	1.3(±0.2) × 10^6^/2.2(±0.1)
Δ-**1** ^2+^/**htel**	2.7(±0.2) × 10^5^/2.7(±0.1)
Λ-**1** ^2+^/**htel**	5.7(±0.2) × 10^5^/1.4(±0.1)

The standard Gibbs free energy change (Δ*G*°) associated with the oxidation of guanine was estimated from the standard potentials for the single reduction of **1** ^2+^ (−0.42 V vs NHE) and oxidation of guanine (+1.22 V vs NHE)^55^ and the excitation energy associated with the emitting MLCT state of **1** ^2+^ (see ESI). Using this approach a Δ*G*° of −0.33 eV was determined, which reflects the thermodynamically favorable electron transfer. Notably, this value is greater than −0.20 eV estimated for the Δ*G*° for the oxidation of guanine by [Ru­(TAP)_2_(dppz)]^2+^, which reflects the higher reduction potential of the parent complex (−0.55 V vs NHE).^50^

#### Time-Resolved Spectroscopic Characterization of **1** ^2+^ Bound to d­(G_5_C_5_)_2_

The TrA spectra of Λ-**1** ^2+^ and Δ-**1** ^2+^ recorded in the presence of **d­(G**_**5**_ **C**_**5**_ **)**_**2**_ at a nucleotide to complex (Nucl/Ru) ratio of 25 in aerated 50 mM phosphate D_2_O buffer, are shown in Figures 5a, and S36. The TrA spectrum of Λ-**1** ^2+^ and Δ-**1** ^2+^ measured at 1 ps after 400 nm excitation shows a broad transient at 600 nm due to the lowest TAP-based ^3^MLCT state [Ru^III^(TAP^•–^)­(TAP)­(dppz-10-CN)]^2+^. The intensity of this band is found to decrease slightly within 50 ps, which is followed between 50 and 1750 ps by the grow-in of a strong absorbance feature at 495 nm accompanied by weak transient bands at 630 and 670 nm. The transient features are in good agreement with the SEC spectrum of the complex comprising the reduced [TAP]^•–^ and [dppz-10-CN]^•–^ ligands, see Figure 3a, which indicates the formation of the singly reduced Ru­(III) metal complex [Ru^III^(TAP^•–^)­(TAP)­(dppz-10-CN^•–^)]^+^ arising from the forward electron transfer (FET) from a close-lying guanine base to the [Ru^III^(TAP^•–^)­(TAP)­(dppz-10-CN)]^2+^ excited state. Using the method previously described by Keane et al.,^22^ the relative yield of formation of the reduced species by the enantiomers was estimated by comparing the intensity of the transient absorbance of the ^3^MLCT excited state (600 nm) at 1 ps to the maximum intensity (at ca. 1 ns) of the reduced complex (505 nm), see Table 3. The results indicate a similar yield of formation for both enantiomers, which is in agreement with what was observed for the reference dppz complex.^22^ Kinetic analysis of the grow-in of the transient band reveals a slight enantiomeric dependence on the rate of forward electron transfer (FET) with a faster rate observed for Λ-**1** ^2+^ (380 ± 20 ps) compared to Δ-**1** ^2+^ (540 ± 30 ps), see Figure S36 and Table 4.

**Figure 5:**
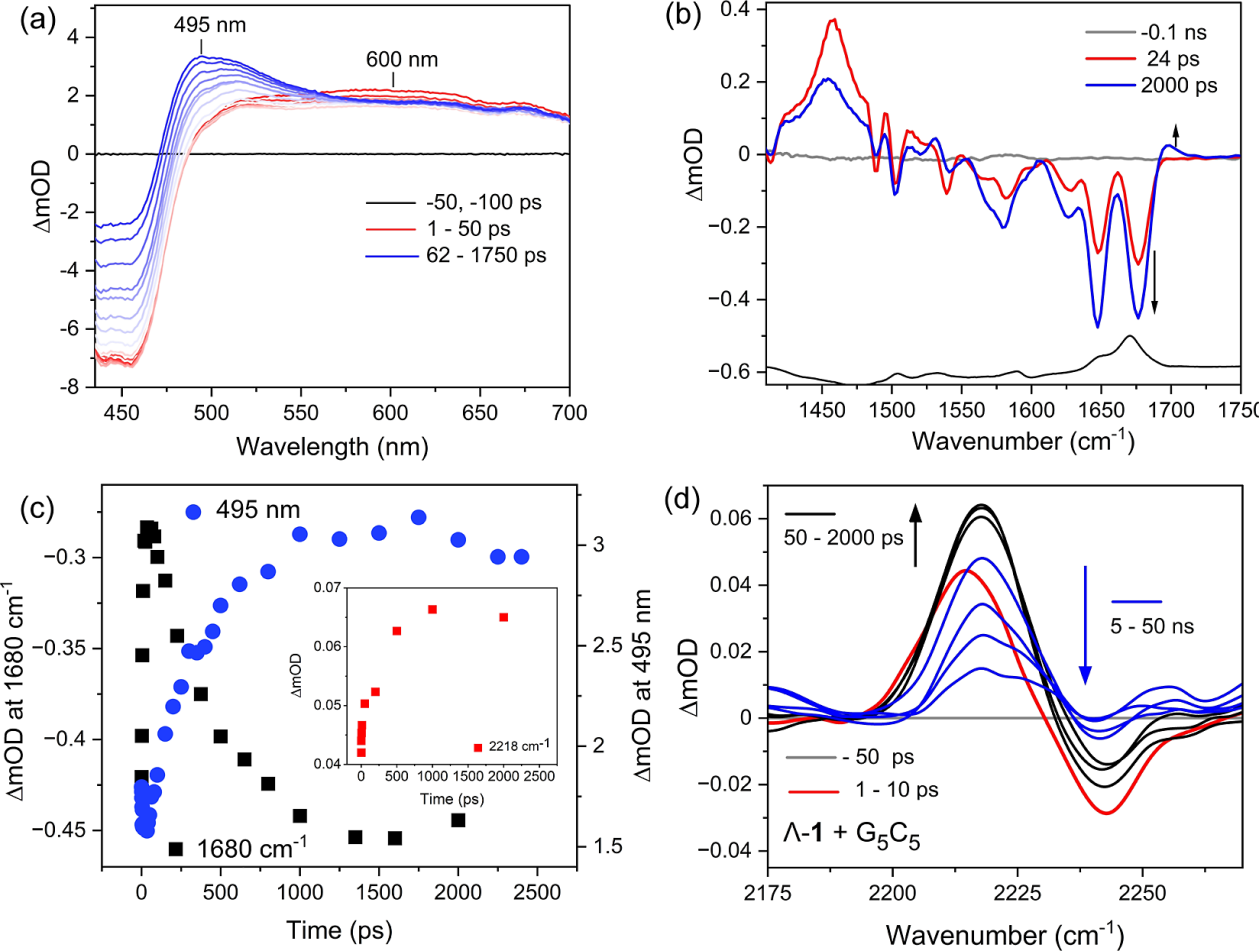
(a) ps-TrA spectra of Λ-**1** ^2+^ (50 μM) in the presence of **(dG_5_C_5_)_2_** in 50 mM phosphate buffer in D_2_O, excitation 400 nm, 150 fs. (b) TRIR difference spectra of 0.4 mM of Λ-**1** ^2+^ in the presence of 0.5 mM (per duplex) **(dG_5_** **C**_**5**_ **)_2_** DNA in 50 mM phosphate buffer in D_2_O, pH 7. (c) Comparative kinetics for the TrA and TRIR (inset showing the change for the nitrile vibration). (d) TRIR difference spectra of 0.8 mM of Λ-**1** ^2+^ in the presence of 1.0 mM (per duplex) (**G_5_C_5_)_2_** DNA in 50 mM phosphate buffer in H_2_O (λ_exc_ = 400 nm, 2 kHz, 150 fs). The TRIR spectra were vertically translated to account for baseline offset.

**Table 3:** Estimated Relative Yields (rY) of FET

system	rY^a^	system	rY^a^
Λ-**1** ^2+^/**G_5_C_5_**	1.6	Δ-**1** ^2+^/G_5_C_5_	1.7
Λ-**1** ^2+^/**hTel**	1.6	Δ-**1** ^2+^/**hTel**	1.1

a rY defined as ΔOD_505 nm(1 ns‑delay)_/ΔOD_600 nm(1 ps‑delay)_ Adapted from ref 22.

**Table 4:** Lifetimes (τ) for the Forward- and Back-Electron-Transfer Reactions Between Guanine and Λ-**1** ^2+^ and Δ-**1** ^2+^ (0.4 mM) in the Presence of 0.5 mM (per Duplex) **G**_**5**_ **C**_**5**_ in the 50 mM Aqueous Phosphate Buffer^a^

selected λ (nm) or *Ṽ* (cm^–1^)	Δ-**1** ^2+^/**G_5_C_5_** τ_(FET)_	Δ-**1** ^2+^/**G_5_C**_**5**_ τ_(BET)_	Λ-**1** ^2+^/**G_5_C**_**5**_ τ_(FET)_	Λ-**1** ^2+^/**G_5_C_5_** τ_(BET)_
505 nm (**1** ^+^)	542 ± 31 ps		378 ± 19 ps	
1650 cm^–1^ (C)	575 ± 35 ps	12.1 ± 0.8 ns	380 ± 19 ps	11.2 ± 0.8 ns
1680 cm^–1^ (G)	556 ± 41 ps	14.5 ± 1.1 ns	400 ± 27 ps	15.8 ± 1.7 ns
1700 cm^–1^ (G^•+^)	531 ± 90 ps	19.2 ± 4.9 ns	321 ± 30 ps	10.6 ± 1.8 ns
	Δ-**1** ^2+^/**hTel** τ_(FET)_	Δ-**1** ^2+^/**hTel** τ_(BET)_	Λ-**1** ^2+^/**hTel** τ_(FET)_	Λ-**1** ^2+^/**hTel** τ_(BET)_
515 nm (**1** ^+^)	1282 ± 74 ps		816 ± 36 ps	
1668 cm^–1^ (G)	1554 ± 97 ps	85.4 ± 11.6 ns	622 ± 18 ps	20.3 ± 1.1 ns
1688 cm^–1^ (G^•+^)	1269 ± 256 ps		533 ± 70 ps	
2217 cm^–1^ (G^•+^)			820 ± 75 ps	

a And of Λ-**1** ^2+^ and Δ-**1** ^2+^ (0.4 mM) in the presence of 1.2 mM **hTel**.

The TRIR spectra, recorded for Λ-**1** ^2+^ under the same conditions as above are shown in Figures 5b and S37a. At 24 ps, the Λ-**1** ^2+^ spectrum shows a strong transient absorption band at 1458 cm^–1^, which is characteristic of the formation of the TAP-based ^3^MLCT state of **1** ^2+^. This is flanked by bleaches of the ground-state vibrations at 1325, and between 1475 and 1515 cm^–1^. Notably, in contrast to the complex alone, a more intense nitrile transient band (2215 cm^–1^) is observed upon photoexcitation, which is taken to reflect the DNA-bound environment of the dppz-10-CN ligand, see Figure 5d. In addition, strong bleaches at 1650 and 1680 cm^–1^ due to the cytosine and guanine carbonyl bands, respectively, are observed. These new bleaches arise due to a perturbation of the bases in the binding site by the MLCT excited state “site effect”.

#### Forward Electron Transfer (<1.5 ns)

After ca. 50 ps the intensities of the 1650 cm^–1^ (cytosine carbonyl) and 1680 cm^–1^ (guanine carbonyl) bleach bands are found to increase, accompanied by the appearance of a transient band at 1704 cm^–1^, which reaches a maximum intensity at 1.5 ns. The change in the intensity of the 1680 cm^–1^ is found to coincide with the change in the intensity at ca. 495 nm, see Figure 5c. These changes are associated with forward electron transfer (FET) from guanine and confirm its photo-oxidation to form G^•+^.^12,18,56,57^ Similar spectra and behavior were observed for Δ-**1** ^2+^ bound to **d­(G**_**5**_ **C**_**5**_ **)**_**2**_, see Figure S37b. Of particular interest to this study is the ability of the nitrile vibration on the dppz-10-CN ligand to report on guanine photo-oxidation, noting that the dppz-10-CN is expected to mediate the FET as evidenced from the transient bands between 600 and 700 nm (Figures 2a and 3a). The "grow-in" of the 1704 cm^–1^ band is accompanied by changes in the nitrile transient band at 2215 cm^–1^, which is observed to grow in intensity and shift to a slightly higher wavenumber of 2218 cm^–1^ over 2.5 ns (Figure 5c (inset) and 5d). This shift of the CN-stretch absorption and enhancement of the intensity reflects the reduction of the dppz-10-CN ligand in the [Ru^III^(TAP^•–^)­(TAP)­(dppz-10-CN^•–^)]^+^ species, which explains why the observed red-shift is smaller than that observed (2205 cm^–1^) for the electrochemically generated (at R1 + R2) [Ru^II^(TAP^•–^)­(TAP)­(dppz-10-CN^•–^)] **(1** ^0^).

#### Back Electron Transfer (>1.5 ns)

After ca. 2.0 ns, the TRIR band at 1460 cm^–1^ starts to decrease, which is accompanied by the recovery of the carbonyl-stretching bleach band of oxidized G^•+^ at 1680 cm^–1^. On this time scale the intensity of the CN-stretching band at 2218 cm^–1^ is also observed to decrease due to the reverse (back) electron transfer (BET) process.

The rate of the BET to guanine in D_2_O solution was examined by fitting the cytosine (1650 cm^–1^) and guanine (1680 cm^–1^) bleach bands, and the G^•+^ transient band (1704 cm^–1^), see Figures S38 and S39. The lifetime for the ET from guanine to Λ-**1** ^2+^ was found to be 365 ± 25 ps, with a slower rate of 555 ± 50 ps observed for Δ-**1** ^2+^, which is in excellent agreement to that determined from the TrA experiments for Λ-**1** ^2+^ (378 ± 19 ps) and for Δ-**1** ^2+^ (542 ± 31 ps). The TRIR data also revealed a slightly slower rate of BET from guanine for Δ-**1** ^2+^ compared to Λ-**1** ^2+^, see Table 4. In the presence of both enantiomers the DNA bleach bands were found to recover with a lifetime of ca. 15 ns, which is considerably faster than that observed for the complex alone. The transient at 2215 cm^–1^ is found to decay with a lifetime of ca. 9 ns, which is comparable to the time scale determined by analysis of the carbonyl vibrations (Figure S40). The slightly faster recovery of the parent nitrile stretch is attributed to an isotope effect, as the nitrile TRIR absorption is recorded in H_2_O. This observed isotope effect for the back electron transfer is consistent with proton-coupled electron transfer from the guanine radical cation to the base-paired cytosine, which occurs due to the stronger acidity of the guanine radical cation compared to the cytosine base. A comparable kinetic isotope effect (*k*_H_/*k*_D_) of 1.6 was also reported for [Ru­(TAP)_2_(dppz)]^2+^ bound to the [poly­(dG-dC)]_2_ homopolymer .^20^

#### Time-Resolved Spectroscopic Characterization of **1** ^2+^ Bound to **hTel**

Having profiled guanine photo-oxidation in the double-stranded DNA, the photo-oxidation of guanine in the quadruplex DNA by Λ-**1** ^2+^ and Δ-**1** ^2+^ was examined. The ps-TrA spectra of Λ-**1** ^2+^ and Δ-**1** ^2+^ in the presence of **hTel** upon 400 nm excitation show a significant “grow-in” of a transient absorption at ca. 505 nm between 50 and 2000 ps again attributed to the formation of the singly reduced complex [Ru^III^(TAP^•–^)­(TAP)­(dppz-10-CN^•–^)]^+^, see Figure 6a,b. Notably, at 2.4 ns the intensity of the Λ-**1** ^2+^ transient absorption is significantly greater than Δ-**1** ^2+^ and suggests a 2-fold increase in the relative yield of FET over 2.5 ns. The rate of FET, monitored by the growth of the transient band at ca. 505 nm, was found to be slower than that observed in double-stranded DNA, with significant differences observed for the enantiomers, Λ-**1** ^2+^ (τ = 816 ± 37 ps) and Δ-**1** ^2+^ (τ = 1282 ± 74 ps), see Figure 6c.

**Figure 6:**
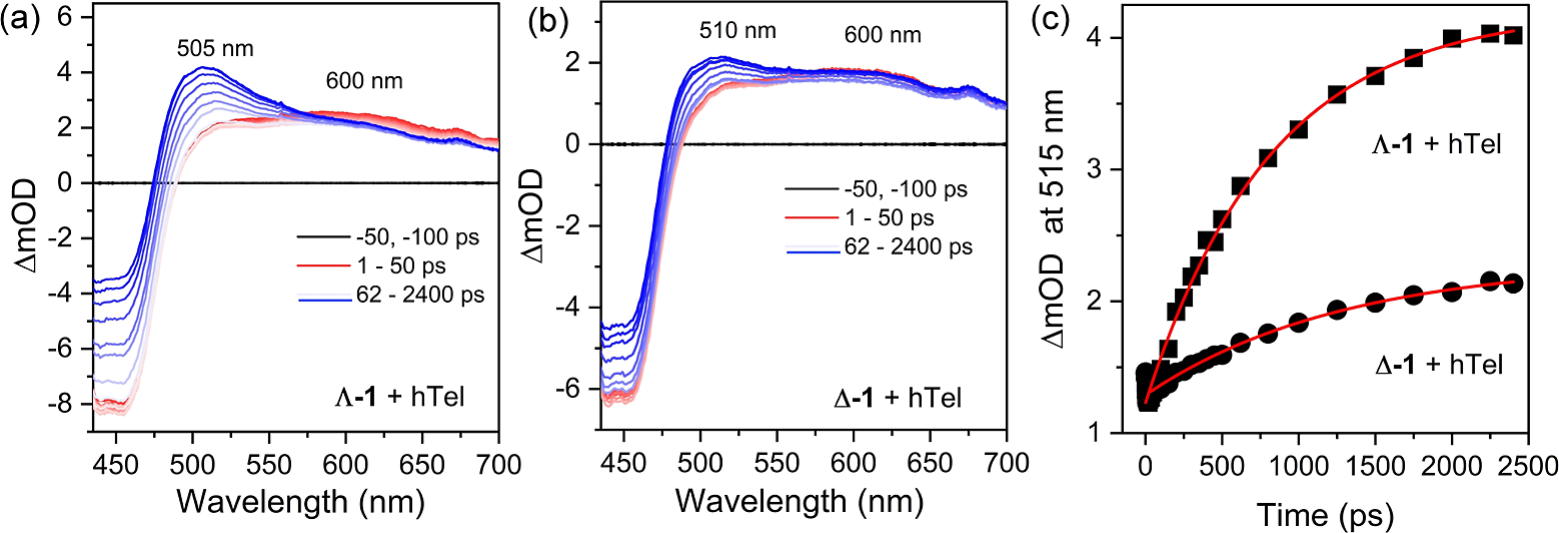
TrA spectroscopy of (a) Λ-**1** ^2+^ (50 μM) and (b) Δ-**1** ^2+^ (50 μM) in the presence of 150 μM **hTel** (per G4) in 50 mM phosphate buffer and 100 mM KCl. (c) Kinetics of the growth of the 505 nm and 510 nm bands in the respective Λ-**1^2+^** and Δ-**1** ^2+^ + **hTel** spectra of (a,b).

The TRIR spectrum of Λ-**1** ^2+^(**hTel**) at 24 ps shows a sharp, strong bleach present at 1668 cm^–1^, which is characteristic of close proximity of the excited state with the G4 bases. The intensity of the bleach is greater for Λ-**1** ^2+^ than Δ-**1** ^2+^, see Figure 7a and Figure S41. The position of the guanine carbonyl bleach is in line with the ground state FTIR spectra of Λ-**1** ^2+^ in the presence of **hTel** DNA, which is dominated by the quadruplex guanine C=O vibration (1670 cm^–1^), where the tetrad arrangement results in a shift in the guanine carbonyl vibration, see Figure 7.^24^ However, there is a notable lack of strong signal from vibrations associated with the thymine and adenine (T–A) bases in the loops; adenine (1628 cm^–1^) and thymine (1641–1645 cm^–1^ (ν_ring_), 1655–1660 cm^–1^ (ν_C4=O4_) and at 1696 cm^–1^ (ν_C2=O2_)). Between 24 and 2000 ps the intensity of the 1670 cm^–1^ bleach band is found to increase, which is accompanied by the formation of a transient band at 1688 cm^–1^ (Figure 7). This observation is in excellent agreement with the “grow-in” of a transient absorption at ca. 505 nm. Comparable changes are also observed for the TRIR band at 1650 cm^–1^ which is also attributed to the guanine radical cation in the quadruplex structure. The appearance of a broad band overlapping the bleach band possibly arises due to the delocalization of the guanine radical cation over more than one base in the tetrad structure. Again, it is noticeable that the magnitude of the bleach grow-in is greater for Λ-**1** ^2+^ than Δ-**1** ^2+^ (1.5:1). At longer times (between 2 and 500 ns) the bleach is found to decay, see Figure S41.

**Figure 7:**
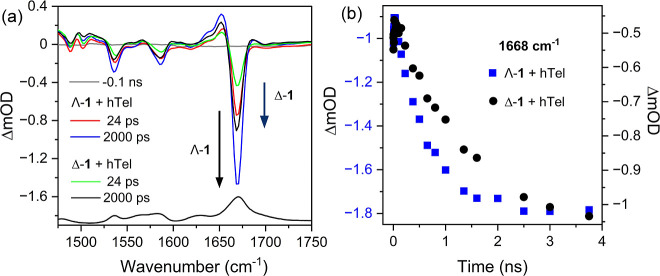
TRIR difference spectra of 0.4 mM of (a) Δ-**1** ^2+^ and Λ-**1** ^2+^ in the presence of 1.2 mM **hTel** (per G4) in 50 mM K-phosphate and 100 mM KCl, pH 7, in D_2_O (λ_exc_ = 400 nm, 2 kHz,150 fs). (b) Comparison of the grow-in of carbonyl bleach associated with forward electron transfer from guanine.

Kinetic analysis of the ps-ns TRIR was performed to profile the dynamics, see Figures S42 and S43. The rate of FET was determined by fitting the “grow-in” of the 1668 cm^–1^ bleach, and the appearance of the transient band (1688 cm^–1^), which were found to be in good agreement with the TrA data (Table 4) giving average values of ca. 575 ps for Λ-**1** ^2+^ and a lower value of ca. 1400 ps for Δ-**1** ^2+^, see Figure S43. Additionally, the rate of BET, determined by monitoring the recovery of the 1668 cm^–1^, is also found to be sensitive to the enantiomer, with the BET rate observed for Δ-**1** ^2+^ ca. four times slower than Λ-**1** ^2+^ (Λ-**1** ^2+^/**hTel** = 20.3 ± 1.1 ns, Δ-**1** ^2+^/**hTel** = 85.4 ± 11.6 ns).

Finally, given the greater yield of forward electron transfer by the Λ-**1** ^2+^, the ability of the nitrile band to report on the dynamics was investigated, see Figure 8. A dramatic change was observed in the TRIR nitrile transient. Excitation results in the appearance of a clear transient band, which is found to undergo modest changes between 1 and 50 ps, see Figure S44a. Between 50 and 2500 ps, an increase in intensity accompanied by a slight shift from 2214 to 2217 cm^–1^ with a lifetime of 820 ± 75 ps, is consistent with FET to **1** ^2+^ in the TAP-based ^3^MLCT state and in excellent agreement with the kinetics observed at 515 nm in the TrA (816 ± 36 ps). The transient was then observed to decay over tens of ns, see Figure S44b.

**Figure 8:**
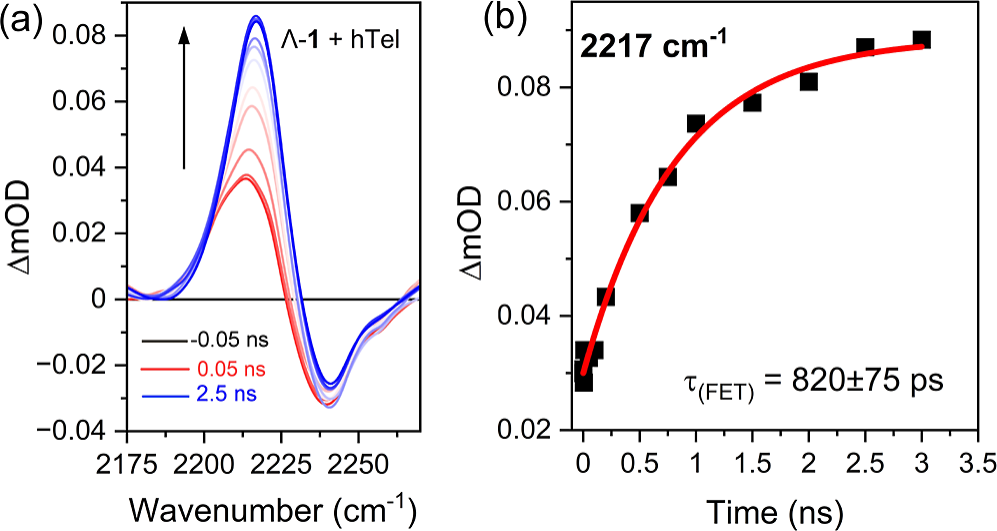
(a) TRIR difference absorbance spectra of 0.8 mM Λ-**1** ^2+^ in the presence of 2.4 mM **hTel** (per G4) in 50 mM K-phosphate and 100 mM KCl, pH 7, in H_2_O (λ_exc_ = 400 nm, 2 kHz,150 fs). (b) Kinetic analysis of the 2217 cm^–1^ band.

## Discussion

UV–vis SEC reveals that the reduction potentials of the TAP and dppz-10-CN ligands are comparable such that it is not possible to exclusively generate the 1e^–^-reduced species, but instead **1** ^2+^ is reduced directly to **1** ^0^. The spectroscopic measurements indicate that optical excitation of **1** ^2+^ at 400 nm leads to formation of TAP-based ^3^MLCT. This is supported by DFT calculations that indicate the lowest excited state to be TAP-based ^3^MLCT. In contrast to phen-based Ru­(II) complexes containing the dppz-11,12-CN and dppz-11-CN, and Ru­(II) CN–Me-bpy complexes, the optically excited complex does not show a strong nitrile transient band, which reflects the largely TAP-based ^3^MLCT excited state.^36–40^ Overall, the nitrile substituent is not observed to impact the nature of the emitting ^3^MLCT when compared to the reference complex [Ru­(TAP)_2_(dppz)]^2+^.^51^

Turning to the binding interactions with DNA. Both enantiomers show good binding affinity to double-stranded DNA, which is comparable to that of the parent complex, *rac*-[Ru­(TAP)_2_(dppz)]^2+^, for which *K*_b_ = 10^6^ M^–1^.^36^ This indicates that the introduction of the nitrile substituent to the 10-position does not impede the binding to the duplex structure, which likely binds through a mode where the nitrile is projected toward the major groove.^42^ The dppz-10-CN ligand does appear to reduce the affinity for the hybrid **hTel** structure (here 10^5^ M^–1^) compared to that previously (10^6^ M^–1^) observed for [Ru­(phen)_2_(dppz-11,12-CN)]^2+^, which also shows greater binding affinity for the Λ-**1** ^2+^ enantiomer over the Δ-**1** ^2+^ enantiomer.^40^ The almost complete emission quenching observed in the presence of **d­(G**_**5**_ **C**_**5**_ **)**_**2**_ and **hTel** is attributed to the presence of guanine in the binding site of the guanine-rich systems and the favorable Δ*G* for guanine oxidation. In the case of **st-DNA**, the observation of incomplete quenching is attributed to binding to AT-rich tracts of DNA. The lack of enantiomer dependent luminescent quenching in the presence of the double-stranded **st-DNA** and **d­(G**_**5**_ **C**_**5**_ **)**_**2**_ is notable, and mirror previous studies on the binding of [Ru­(TAP)_2_(dppz)]^2+^ enantiomers to **st-DNA** and **d­(G**_**5**_ **C**_**5**_ **)**_**2**_ systems.^12,58^ Crystallographic studies of ruthenium polypyridyl complexes have revealed a range of intercalation modes for dppz ligands, which adopt an orientation that maximizes purine/dppz stacking interactions.^15^ Enantiospecific interactions are typically associated with a difference in the binding site overlap with the base pairs and is found to be sequence dependent.^12,15^ Structural studies of the DNA binding of enantiomers of the iso-structural [Ru­(TAP)_2_(dppz)]^2+^ and [Ru­(phen)_2_(dppz)]^2+^ complexes indicate that in excess DNA the Δ-enantiomer preferentially binds to a GC/GC step^22^ while the Λ enantiomer preferentially binds to a CC/GG step.^59^ Thus, the lack of enantiospecific quenching is explained by the presence of both GC/GC and CC/GG steps in **d­(G**_**5**_ **C**_**5**_ **)**_**2**_ that provide binding sites with good overlap between the dppz ligand and a guanine base. Notably, in contrast to what is observed for st-DNA, the change in emission observed for **hTel** is sensitive to the enantiomer form, Figure 4c. At 1:1 equiv of B-DNA the emission of both enantiomers is almost completely quenched, while at 1:1 equiv **hTel** DNA with 80% quenching observed for Λ-**1** ^2+^ and 50% for Δ-**1** ^2+^. There are relatively few reports of enantioselective binding of ruthenium polypyridyl complexes to quadruplex DNA, a notable example being the recent report for the related [Ru­(phen)_2_(dppz-11-CN)]^2+^ complex^60^ and the extended Λ-[Ru­(phen)_2_(qdppz)]^2+^ complex, which was observed to inhibit replication of the human telomeric sequence.^61^ While a modest difference was observed in the **hTel** binding affinity of Δ- and Λ-[Ru­(phen)_2_(dppz)]^2+^.^62^ Ruthenium polypyridyl complexes are known to bind to G4 DNA through end stacking interactions and are impacted by loop interactions.^24,40,60–64^ It is expected that the enantiospecific binding observed here is due to differences in the G-tetrad overlap that arise due to the loop arrangement of the hybrid potassium stabilized hybrid G4 structure, and a possible effect of the substitution at the 10-position.^40,60^

The observation of emission quenching in the G-rich systems suggests the role of electron transfer. In the case of double-stranded B-DNA **d­(G**_**5**_ **C**_**5**_ **)**_**2**_, the transient absorption studies combined with the SEC and DFT allow mechanisms for both the FET and BET to be proposed, see eqs 1 and 2. In the case of **d­(G**_**5**_ **C**_**5**_ **)**_**2**_, the TrA reports on the FET process with a "grow-in" of the band at ca. 500 nm within 2 ns that indicates formation of the singly reduced metal complex. This is accompanied by a further "grow-in" of the guanine bleach at 1668 cm^–1^ and the appearance of an IR transient at 1704 cm^–1^, which reports on the formation of the guanine radical cation, Figure 5a–c. Critically, on the same time scale, an enhancement of the nitrile vibration is observed, Figure 5d. The kinetics reveal a slight lag between the loss of the TAP transient and the evolution of the nitrile transient. This suggests a sequential process whereby the BET transfer is initiated by electron transfer from [TAP]^•–^ to the Ru­(III) metal center, which then leads to back electron transfer from [dppz-10-CN]^•–^ to the guanine radical cation (G^•+^). The kinetic isotope effect of 1.6 between H_2_O and D_2_O (*k*_H_/*k*_D_) observed for the BET in the case of **1** ^2+^ bound to **d­(G**_**5**_ **C**_**5**_ **)**_**2**_ is consistent with the role of proton-coupled electron transfer between the hydrogen-bonded nucleobases upon photo-oxidation (**G**≡**C** → **G** ^•^≡**C**H^+^, where C = cytosine and G = guanine, ≡ represents the hydrogen bonded nucleobases).

Forward electron transfer

$${\left[{Ru}^{II}{\left(TAP\right)}_{2}\left(dppz‐10‐CN\right)\right]}^{2+}+G\to^{3}{\left[{Ru}^{III}\left({TAP}^{•-}\right)\left(TAP\right)\left(dppz‐10‐CN\right)\right]}^{2+}+G\to {\left[{Ru}^{III}\left({TAP}^{•-}\right)\left(TAP\right)\left(dppz‐10‐{CN}^{•-}\right)\right]}^{+}+G^{•+}$$ 1

Back electron transfer

$${\left[Ru^{III}\left(T{AP}^{•-}\right)\left(TAP\right)\left(dppz‐10‐{CN}^{•-}\right)\right]}^{+}+G^{•+}\to {\left[{Ru}^{II}{\left(TAP\right)}_{2}\left(dppz‐10‐{CN}^{•-}\right)\right]}^{+}+G^{•+}\to {\left[{Ru}^{II}{\left(TAP\right)}_{2}\left(dppz‐10‐CN\right)\right]}^{2+}+G$$ 2

The observed changes are even more clearly seen for the **hTel** system where BET to the close-lying guanine base is again triggered by transfer from the [TAP]^•–^ species and then facilitated by the intercalated [dppz-10-CN]^•–^ state (eq 2).

BET from the singly photoreduced [Ru­(TAP)_2_(dppz)]^+^ to G^•+^ has previously been monitored in solution by TrA and in DNA crystals by TRIR and found to occur between 7 and 15 ns.^12,16^ However, the incorporation of the nitrile probe on the intercalating dppz-10-CN ligand in **1** ^2+^ provides an additional insight into the mechanism and the role of the intercalated [dppz-10-CN]^•–^ species in mediating this process. This complements a recent study on the complex [Cr­(TMP)_2_(dppz)]^3+^ where optical excitation resulted in the formation of an energetic ^1^LC excited state based on the intercalated dppz ligand, which was capable of oxidizing adenine and also facilitated rapid FET (<1 ps) and BET (ca. 100 ps) processes in G-rich DNA.^21^ In contrast to the chromium­(III) system, the TAP-based [Ru^III^(TAP^•–^)­(TAP)­(dppz-10-CN)]^2+^ excited state in **1** ^2+^, with the Ru^III^–TAP^•–^ charge separation initially persisting as the photoreduced **1** ^+^ (eq 1), is spatially removed from G^•+^. This results in delaying the BET process and thus understanding the intermediate involved in facilitating FET is significant.

The TrA spectra reveal slight differences in the rate of FET with a faster rate observed for Λ-**1** ^2+^ (378 ± 19 ps) compared to Δ-**1** ^2+^ (542 ± 31 ps), which is attributed to differences in the binding associated with the CC/GG and GC/GC steps. This is in good agreement with the trend observed in previous studies on the parent complex [Ru­(TAP)_2_(dppz)]^2+^ bound to **d­(G**_**5**_ **C**_**5**_ **)**_**2**_, where the FET observed for the Λ-enantiomer was 460 ± 70 and for the Δ 970 ± 150 ps.^22^ The faster FET processes in the case of **1** ^2+^ may be explained by the lower reduction potential (−0.42 V compared to −0.55 V, vs NHE). Interestingly, for [Ru­(TAP)_2_(dppz)]^2+^ bound to **d­(G**_**5**_ **C**_**5**_ **)**_**2**_ the differences in FET did not result in significantly different relative yields of ET,^22^ which is also the case for the similar, and high relative yield of ET observed for Λ-**1** ^2+^ and Δ-**1** ^2+^. Indeed, the study by Keane et al. noted that for intercalator systems the yields and rates of ET are not necessarily proportional. Additionally, guanine photo-oxidation by the parent [Ru­(TAP)_2_(dppz)]^2+^ in model oligonucleotide systems observed **d­(G**_**5**_ **C**_**5**_ **)**_**2**_ to be the only system where reverse ET was more efficient for Λ-**1** than Δ-**1**.^22^ Together these results strongly suggest that the nitrile substitution at the 10-position does not significantly impact the binding to **d­(G**_**5**_ **C**_**5**_ **)**_**2**_.

Contrasting behavior is observed for the **hTel** system. While the Λ enantiomer again showed faster FET, these rates were appreciably lower than that observed for double-stranded **d­(G**_**5**_ **C**_**5**_ **)**_**2**_ DNA, see Table 4. The impact of the different enantiomeric binding to **hTel** DNA has previously been reported for the related light-switch [Ru­(phen)_2_(dppz)]^2+^ and [Ru­(phen)_2_(dppz-11,12-CN)]^2+^ complexes.^41,58^ In the case of **1** ^2+^, the enantiomeric difference is likely due to different orientations in the binding site, which is reflected in the different binding affinities of the two complexes, see Figure S33. Significantly, the clear difference in binding results in a significant difference in the yield of reduced species formed by 2400 ps. In both cases good agreement between the kinetic analyses of the TRIR and TrA data was observed.

As expected, in the case of **d­(G**_**5**_ **C**_**5**_ **)**_**2**_ the TRIR is dominated by guanine and cytosine bleaches that reflect binding to the **GC** site. For **hTel** the strong carbonyl bleach associated with the quadruplex guanine tetrad combined with the absence of bleaches associated with the thymine and adenine bases in the (TTA) loops suggests that the major binding interaction for both enantiomers is with the G-tetrad. Notably, monitoring the recovery of the carbonyl bleach band at 1668 cm^–1^ in the TRIR spectrum indicates that the charge-separated state is stabilized for significantly longer in quadruplex **hTel** (ca. 20 ns for Λ-**1** ^2+^, and >50 ns for Δ-**1** ^2+^) than in double-stranded **d­(G**_**5**_ **C**_**5**_ **)**_**2**_ DNA, and notably longer than that observed for the parent reference [Ru­(TAP)_2_(dppz)]^2+^ complex in the presence of GC-rich DNA systems.^12^ Such slow back electron transfer has potential implications for the DNA photodamage.

While nitrile probes as reporters of ground-state environment have been highlighted in the work of Boxer and co-workers^29,33,34^ and of Bagchi and co-workers,^35^ who have used them to report on biologically relevant systems, there have been very few examples of the use of nitrile probes to report on excited-state processes. One example is their use to quantify the effect of changing electric fields on the active enzyme site by monitoring the magnitude of the electrostatic perturbation introduced by photoexcitation of a fluorescent analogue of the reaction intermediate.^65^ In that study, photoexcitation of a coumarin-183 analogue resulted in a change in the intensity of the nitrile transient band observed using ns-TRIR, which was found to track the excited state process of the dye. In a significant development, we exploited the photoenhancement of the nitrile stretching vibration in the MLCT excited state of [Ru­(phen)_2_(dppz-11,12-CN)]^2+^, to report on the binding environment in quadruplex DNA.^40^ In this study by pivoting the lowest ^3^MLCT state to the nonintercalating (ancillary) TAP ligand, we report a probe with a weak nitrile vibration in the excited state, whose signal becomes greatly enhanced upon photo-oxidation of guanine-rich DNA and can be used to monitor FET and BET events in the IR window.

## Conclusions

IR probes are finding increasing application in cellular studies and have been used to image metabolic processes.^66^ Related to this, we have recently reported the use of TRIR to monitor the excited states of probes in cells.^67^ In this study the presence of an IR redox probe serves to both modulate, and monitor photoinduced ET from DNA. There are exciting developments related to both roles. On the one hand the ability to design sensitizers that cause long-lived charge-separated states in DNA is expected to impact the effectiveness of phototherapies based on direct electron transfer, which will be governed by the rate of BET. Additionally, the ability to detect the enhanced nitrile vibration associated with guanine photo-oxidation, in the biological window, points to the potential of monitoring photo-oxidation in cells. Future work will consider the ability to tune the rate of BET by combining suitable nitrile containing ancillary and intercalating ligands to prepare complexes capable of targeting guanine-rich DNA such as G4 structures, which are highly prevalent in oncogenes and show an excellent ability to stabilize charge-separated states. Additionally, the incorporation of nitrile containing ligands in chromium polypyridyl complexes^21^ would allow monitoring of the enhanced electron transfer processes that occur in those systems and the photo-oxidation of nucleobases such as adenine.

## Supplementary Material



## Notes

Additional information on the synthesis and characterization of the system including spectroscopic and computational data is available (PDF)
