# Supplementary material for: Eyes on the Prize: Tracking Electron Transfer in G‑Rich Duplex and Quadruplex DNA Using Enantiopure Ruthenium Polypyridyl Infrared Redox Probes
Source: J Am Chem Soc. 2025 Aug 10;147(33):29801–14. doi: 10.1021/jacs.5c05736 (PMC12371865; doi:10.1021/jacs.5c05736)
Supplement: Supplementary file 1 [file ja5c05736_si_001.pdf]

Supporting Information

Eyes on the Prize: Tracking Electron Transfer in G-rich Duplex and Quadruplex DNA using Enantiopure Ruthenium Polypyridyl Infrared Redox Probes

Mark Stitch,[1] Martin Pižl,[2,3] Niamh Lehane,[1] Gregory M. Greetham,[4] František Hartl,[2]* Michael Towrie[4] and Susan J. Quinn[1]*

e-mail: f.hartl@reading.ac.uk, susan.quinn@ucd.ie;

[1]School of Chemistry, University College Dublin, Dublin 4, Ireland.

[2]Department of Chemistry, University of Reading, Whiteknights, Reading RG6 6AX, United Kingdom

[3]Department of Inorganic Chemistry, University of Chemistry and Technology Prague, Technická 5, 166 28 Prague 6, Czech Republic.

[4]Science and Technology Facilities Council, Rutherford Appleton Laboratory, Research Complex at Harwell, Didcot, Oxfordshire, OX11 0QX, United Kingdom

Materials

5-guanosine monophosphate (GMP) and salmon testes DNA (stDNA) were supplied by Sigma Aldrich. 5'-TTGGGTTAGGGTTAGGGTTAGGGA-3' (molar absorptivity 244300 M^{-1} cm^{-1} /single-strand) 5'-GGGGGCCCCC-3' (molar absorptivity 163400 M^{-1} cm^{-1}/double-stranded), and were synthesized by Eurogentec (Liege, Belgium) and obtained in a HPLC-purified form. Oligodeoxynucleotide concentrations were determined spectrophotometrically using the single-strand molar absorption coefficients at 260 nm shown in parenthesis. and made up in D_2O in presence of 50 mM potassium phosphate buffer, pH 7.

Synthesis of [Ru(TAP)$_2$(dppz-10-CN)]Cl$_2$ ([**1**$^{2+}$]Cl$_2$)

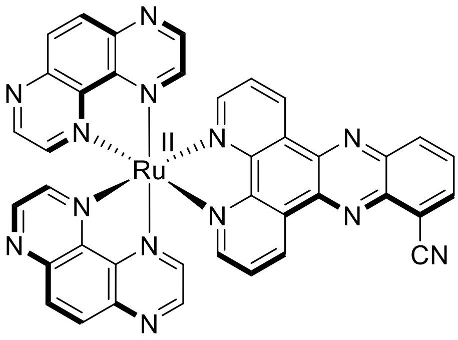

[Ru(TAP)$_2$Cl$_2$] (195 mg, 0.36 mol) and dppz-10-CN (119 mg, 0.38 mol) were suspended in ethylene glycol (15 mL) in a microwave tube. The purple solution was degassed with N_2 for 15 min at room temperature. The tube was placed in a microwave reactor at 418 K for 45 min. After allowing the brown solution to cool, it was filtered, washing with water. The target complex **1**$^{2+}$ was precipitated from the filtrate by the addition of NH_4PF_6. The crude orange product was filtered, washed with water, and dried with diethyl ether. The complex was converted back to the chloride salt by gently shaking it with Amberlite exchange resin in MeOH (10 mL) overnight. Methanol containing extracted [**1**$^{2+}$]Cl$_2$ was removed by rotary evaporation. The resulting crystals were dissolved in water and the solution was filtered through celite to remove impurities. The complex was further purified on a CM Sephadex C25 column, with NaCl stock solutions of increasing concentration ratios. The aqueous solvent was removed, and the resulting precipitate was washed with MeCN to remove the residual salt. Additional counterion exchange was performed using NH_4PF_6 to obtain the PF_6^- salt which was used for CV/SEC measurements. ^{1}H NMR (500 MHz, D_2O) δ 9.80 (dd, *J* = 8.2, 1.3 Hz, 1H), 9.69 (dd, *J* = 8.3, 1.3 Hz, 1H), 8.98 – 8.86 (m, 4H), 8.66 (dd, *J* = 8.6, 1.3 Hz, 1H), 8.58 (d, *J* = 1.2 Hz, 4H), 8.51 (dd, *J* = 7.2, 1.3 Hz, 1H), 8.42 (dd, *J* = 6.9, 2.9 Hz, 2H), 8.29 (t, *J* = 2.5 Hz, 2H), 8.19 – 8.12 (m, 2H), 8.10 (dd, *J* = 8.7, 7.2 Hz, 1H), 7.82 (ddd, *J* = 8.3, 5.5, 1.4 Hz, 2H).FTIR CN vibrational stretch 2240 cm^{-1}. HRMS (ES); *m/z* calc. for $[RuC_{39}H_{21}N_{13}]^{2+}$: 386.5543, found: 386.5541 [M^{2+}].

Chiral resolution was performed using CM Sephadex C-25 column eluting with an aqueous solution of (−)-*O*,*O'*-dibenzoyl-L-tartrate (Sigma-Aldrich) (0.1 M) and the pump speed was gradually increased to 1 mL min^{-1}.

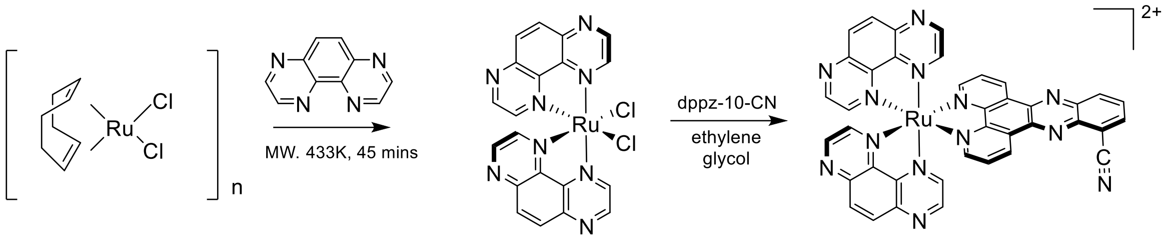

Scheme S1. Synthesis of $\mathbf{1}^{2+}$ (prepared as both the chlorido and PF_6^- salts).

Instrumental methods

UV-Vis absorption spectra were recorded on a Varian Cary 200 spectrophotometer. Steady-state luminescence spectra were recorded on a Varian Cary Eclipse. Circular dichroism measurements were recorded on a JASCO J180. Infrared spectra were recorded on a Nicolet Avatar FT-IR spectrometer or Varian 3100 FT-IR spectrometer fitted with a Universal ATR Sampling Accessory for solid samples or N_2 flushed liquid samples between CaF_2 plates with an optical path length of 100 or 150 µm and at a resolution of 4 cm^{-1} and 256 scans. Solution samples for transient spectroscopy measurements were prepared by dropping a known volume of solution (25–35 µL) between two CaF_2 (25-mm diameter) windows (Crystran Ltd, UK), separated by a 50-µm Teflon spacer, in a demountable solution IR cell (Harrick Scientific Products Inc., New York).

Cyclic voltammetry (CV) was performed under an inert argon atmosphere with a heart-shaped single-compartment glass cell containing a coiled Ag wire pseudo-reference electrode, a coiled Pt wire counter electrode, and a variety working electrode: 0.4-mm diameter Pt microdisk, 0.4-mm diameter Au microdisk or glassy carbon (GC) microdisk. The cell was held inside an earthed Faraday cage and connected to a Metrohm-Autolab PGSTAT302N potentiostat. The internal standard ferrocene/ferrocenium (Fc^+/Fc) was added prior to the final scans. The sample solutions were 1 mM in analyte dissolved in dichloromethane (DCM) or butyronitrile (PrCN) containing 10^{-1} M tetrabutylammonium hexafluorophosphate (TBAH).
Spectroelectrochemical measurements were performed using an optically transparent thin-layer electrochemical (OTTLE) cell[1] equipped with a Pt minigrid working electrode. The sample solution contained 2×10^{-1} M TBAH as the supporting electrolyte and 1.5 mM redox-active compounds for IR and 0.75 mM for UV-Vis SEC. The course of the spectroelectrochemical experiment was monitored by thin-layer cyclic voltammetry (v = 2 mV s^{-1}), with a potential control by a PalmSens EmStat3+ potentiostat operated with the PSTrace5 software.
IR spectroelectrochemistry was conducted using the Bruker Vertex 70v FT-IR spectrometer equipped with a pyroelectric DTLaGS detector. UV-Vis spectroelectrochemical measurements

were performed using a Scinco S-3100 spectrophotometer with a diode array detector (200–1100 nm).

Computational Methods

DFT calculations on dppz-10-CN and 1^{2+}

All calculations were performed in Gaussian 16, Revision C01 (G16)[2] together with the three-parameterized Becke, Lee, Yang Park (B3LYP) functional.[3-4] For the Ru atom, we used a quasi-relativistic effective-core pseudopotential and a corresponding optimized basis set.[5] For nonmetal atoms, we used the 6-311G(d) basis set.[6] Solvent effects (DCM) were described by the conductor-like polarizable continuum model (CPCM).[7] Open shell systems were calculated using the unrestricted Kohn-Sham approach (UKS) and time-dependent DFT (TDDFT) was used to calculate electronic transitions and analyze them in terms of contributing one-electron excitations.[8]

TRIR measurements using LIFEtime apparatus

TRIR measurements were performed using the LIFEtime and ULTRA apparatus at the Central Laser Facility.[9-10] Briefly, a dual regenerative amplifier laser system (Pharos, Light Conversion) was used to generate 1030 nm synchronized probe (100 kHz, 0.060 mJ, 180 fs) and pump (0.01–50 kHz, 0.150 mJ, 260 fs) pulses. The probe laser was used to drive two optical parametric amplifiers (OPA) (Orpheus-ONE, Light Conversion), which generated tunable mid-IR probe light via successive steps of optical parametric generation in BBO, KTA and difference frequency generation in GaSe. The two independently tunable mid-IR probe beams were focused through the sample with <70 μm spot sizes (FWHM) and dispersed onto two separate 128-pixel MCT array detectors (IR Associates). The pump laser was used to pump a BBO-based OPA (Orpheus-HP, Light Conversion), generating 400 nm pump light from the second harmonic of the signal. The 400 nm pump light was delivered to the sample via a 1.2 m length two-pass delay stage (Newport) and focused through the sample with a spot size of 150 μm FWHM (pump-probe polarization was set at magic angle). Pump-probe delays were set using a combination of the delay stage (0-16 ns) and seeding of the two regenerative amplifiers with different seed pulses from their common oscillator (giving delays in 12 ns out to 10 µs). 100 kHz probing allows measurements multiple probing to milliseconds before the sample is pumped again. In the present experiments, the samples were observed to recover on a microsecond timescale, and so 50 kHz pumping in combination with rastering of the sample was used. The 400 nm pump energy was 500 nJ.

ps-TA (400 nm)

The TA experiments were performed on ULTRA apparatus at the Central Laser Facility.[10] For the ps-TA measurements, both pump and probe pulses were generated by the ULTRA laser system, comprising of a dual output titanium sapphire chirped pulse amplifier (Thales Laser), producing an 800 nm (0.8 mJ) output with 40 fs pulse duration and a repetition rate of 10 kHz, which is split to generate the pump and probe pulses. The white light continuum (WLC) was generated in a CaF_2 plate, which was continuously rastered to avoid color center formation and to improve stability in the probe. The WLC was dispersed through the grating monochromator and detected using a linear silicon array (Quantum Detectors). In front of the monochromator, long-pass filter was placed in order to remove scatter from the excitation beam. For the pump beam a spot size of 150 μm FWHM (pump-probe polarization was set at magic angle) and 500 nJ was used.

All spectra were processed using the in-house Ultraview software provided by the Central Laser Facility (CLF). Samples were calibrated from pixels into wavenumbers using the Ultracal software. The metal complex and DNA region (1250–1860 cm^{-1}) was calibrated by fitting to the absorption bands of polystyrene. All experiments were carried out at 298 K and samples were checked before and after the experiment by UV-Visible spectroscopy (PerkinElmer Lambda 950 spectrophotometer) and FTIR in a Nicolet Avatar spectrometer.

Kinetic analysis

Kinetics were evaluated using Origin software by fitting the data using either a mono or biexponential fitting function. In the case of the TRIR data, the influence of any background absorption was removed by subtracting the ΔOD at 1750 cm^{-1}, where no bands are present.

Estimation the standard Gibbs free energy change (Δ*G*)

The standard Gibbs free energy change (Δ*G*) was estimated using the Rhem-Weller equation below:

Equation 1: $\Delta G° = E° ((D^+/D) - E° (A/A^-) - h\nu$

Where ΔG^0 is the standard Gibbs free energy, *E*° are the standard potentials of donor and acceptor, *hv* is the excited state energy of photo-excited ruthenium complex $\mathbf{1}^{2+}$, while electrostatic terms are neglected. The value of *h*v was determined from the emission maximum at 630 nm to be 1.97 eV and and the one-electron reduction potential of the neutral guanyl radical in the GC base pair of duplex DNA to be +1.22 V (vs. NHE).[11] It should be noted that the oxidation potential of guanine is known to be dependent on to the stacking interactions of the particular adjacent bases and so this is an approximation for the G4 system.

The first reduction potential of $\mathbf{1}^{2+}$ of −0.42 V (vs. NHE) was obtained from the experimental value of −1.14 V (vs. Fc^+/Fc) using the conversion factor of +0.72 V.[12-13]
Inserting below yields a ΔG° of −0.33 eV.

$$\Delta G^\circ = E^\circ\ ((\mathbf{G}^{\bullet+}/\mathbf{G}) - E^\circ\ (\mathbf{1}^{2+}/\mathbf{1}^{+}) - h\nu$$

Determining the relative yield of ET

The relative yields of electron transfer for $\mathbf{1}^{2+}$ enantiomers fully bound to the ODNs were estimated by comparing the ratios of the ΔOD signals for singly reduced $\mathbf{1}^{+}$ and the initially formed ^{3}MLCT excited state of $\mathbf{1}^{2+}$. The signal intensity at 600 nm was recorded at early times (1 ps), giving an indication of the amount of photo-excited Ru complex formed. The signal intensity for the reduced species was then recorded at its maximum (~1000 ps delay) at the peak maximum (~515 nm). The ratio ΔOD 505 nm/ΔOD 600 nm therefore gives an indication of the amount of excited complex undergoing electron transfer. Although it does not equate to the absolute quantum yield of electron transfer, it allows for the relative yields to be compared for different systems when recorded under similar conditions.

Figures

Spectrum Peaks

m/z	Z	Abund	Diff (ppm)	Height %	Height % (Calc)	Ion Species	Formula
383.5556	2	3596	0.44	12.76	13.45	M+2	C39H21N13Ru
384.0571	2	1861	0.88	6.60	6.35	M+2	C39H21N13Ru
384.5551	2	1703	-0.15	6.04	6.01	M+2	C39H21N13Ru
385.0547	2	10155	0.08	36.03	33.35	M+2	C39H21N13Ru
385.5543	2	13928	-0.28	49.42	45.73	M+2	C39H21N13Ru
386.0548	2	17993	0.09	63.84	59.31	M+2	C39H21N13Ru
386.5541	2	28183	-0.56	100.00	100.00	M+2	C39H21N13Ru
387.0558	2	11773	1.09	41.77	41.21	M+2	C39H21N13Ru
387.5548	2	15059	0.32	53.44	54.30	M+2	C39H21N13Ru
388.0558	2	6244	0.04	22.15	22.66	M+2	C39H21N13Ru
388.5566	2	1176	-1.20	4.17	5.07	M+2	C39H21N13Ru
398.5534	2	718	0.79	100.00	100.00	(M+H+Na)+2	C39H21N13Ru
399.0539	2	311	-0.66	43.35	41.22	(M+H+Na)+2	C39H21N13Ru
399.5532	2	505	-0.85	70.31	54.30	(M+H+Na)+2	C39H21N13Ru

Compound Spectra

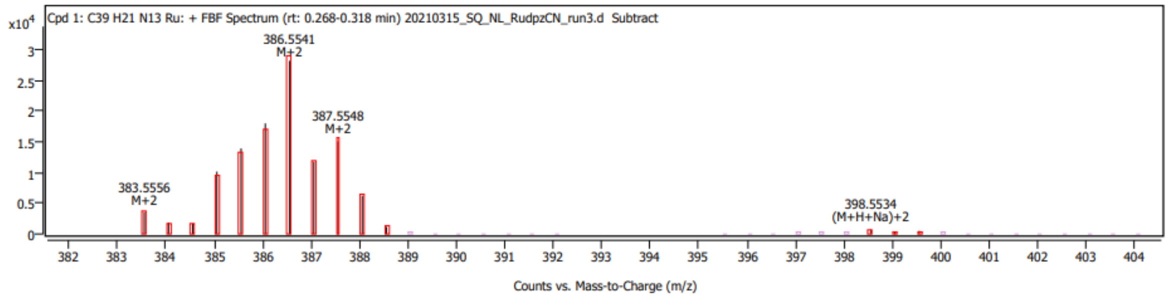

Figure S1: HRMS data for [**1**$^{2+}$]Cl_2.

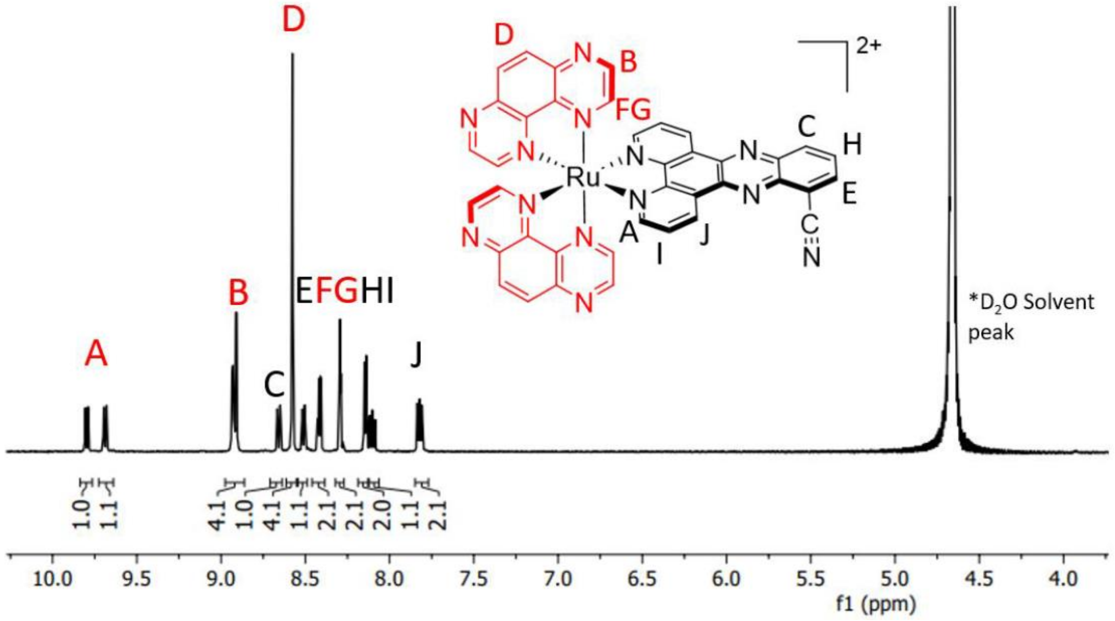

Figure S2: ^{1}H NMR spectrum of [**1**$^{2+}$]Cl_2 (500 MHz, D_2O).

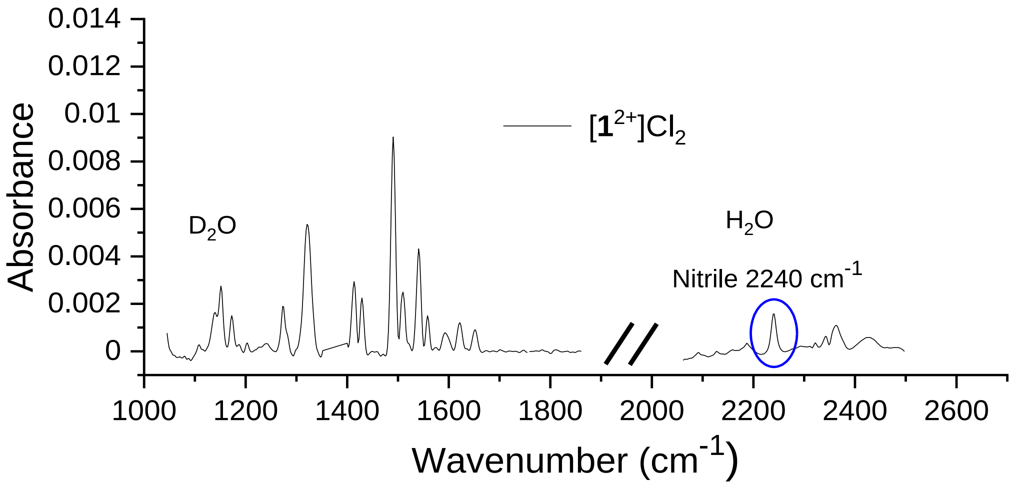

Figure S3. Combined FTIR spectra of [**1**$^{2+}$]Cl$_2$ recorded in H_2O and D_2O, showing the nitrile ν(C≡N) band at 2240 cm^{-1}.

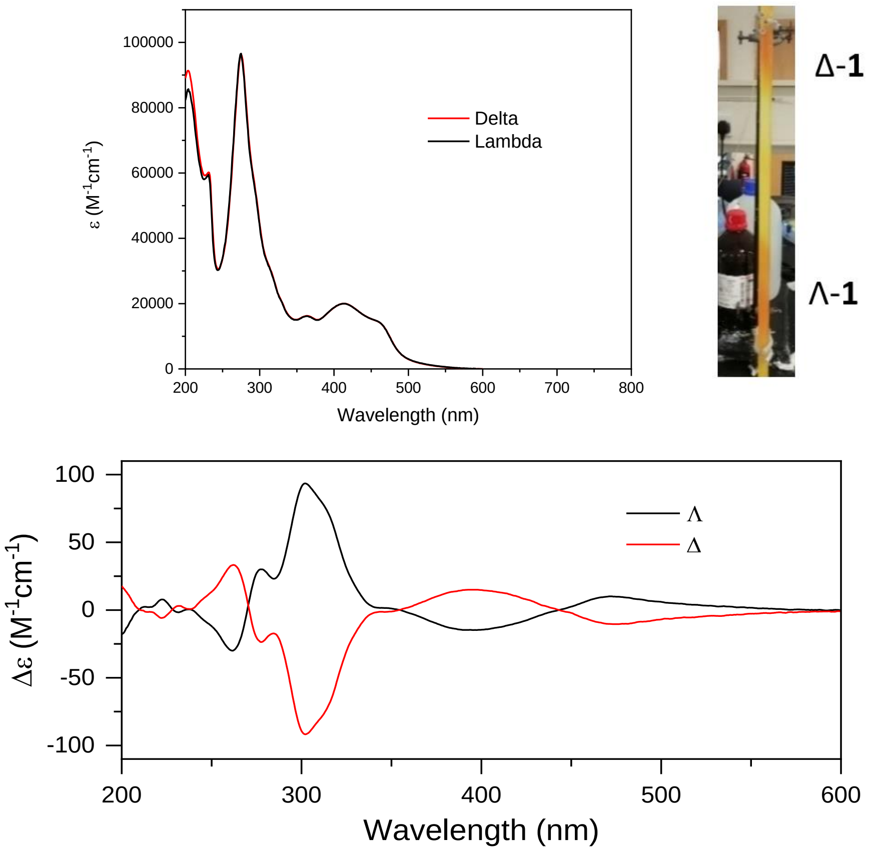

**Δε M^{-1} cm^{-1}**	**Δε(261 nm)**	**Δε(303 nm)**	**Δε(393 nm)**	**Δε(472 nm)**
Δ	33	–93	15	–10
Λ	-31	94	-15	10

Figure S4. (top) Comparative UV-Vis absorption the of enantiomers of **1**$^{2+}$ after tartrate removal and an image of the chiral column (elution using eluted with a (−)-O,O′-dibenzoyl-L-tartrate mobile phase) on the 2nd pass. (bottom) CD spectra of Λ-**1**$^{2+}$ (10.3 µM, black) and Δ-**1**$^{2+}$ (10.4 µM, red) and their tabulated Δε values in an aqueous solution.

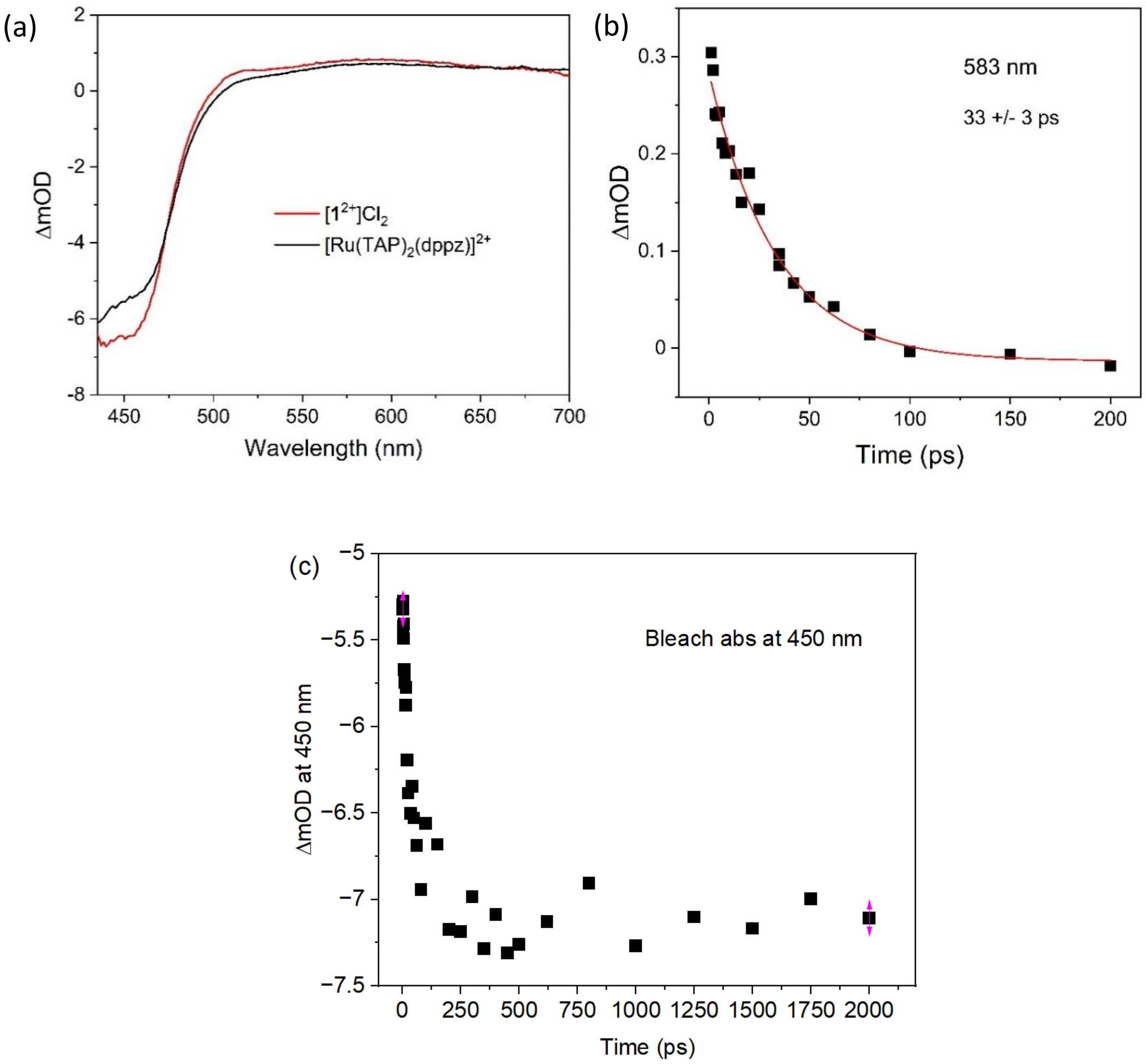

Figure S5. (a) Overlap of TrA of [**1**$^{2+}$]Cl_2 and reference [Ru(TAP)$_2$(dppz)]$^{2+}$ recorded at 50 ps upon 400-nm excitation. (b) Kinetic analysis of [**1**$^{2+}$]Cl_2 recorded in D_2O upon 400-nm excitation. (c) Comparaitve profile of the change in profile fo the bleach absorbace at 450 nm.

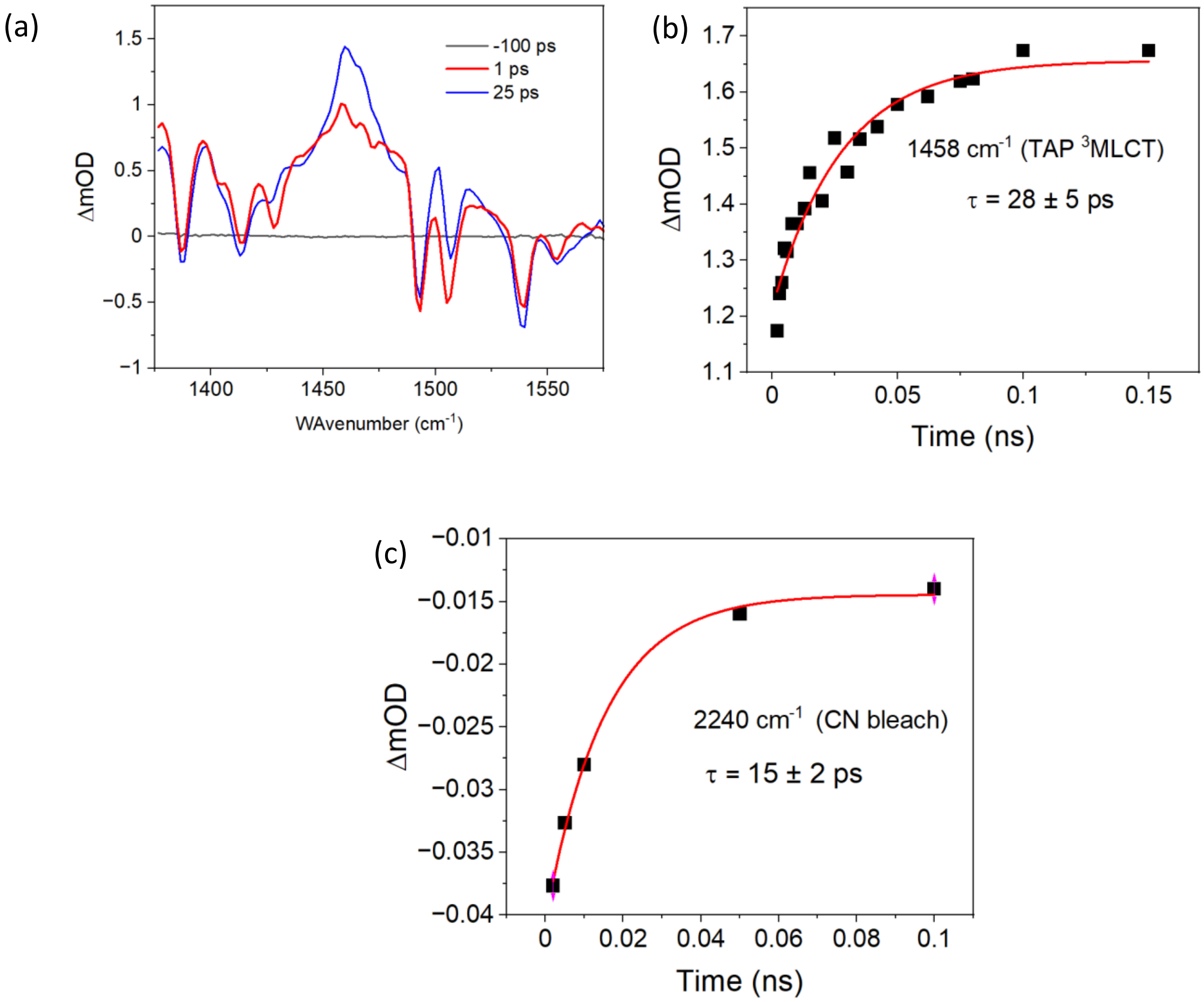

Figure S6 (a) TRIR difference spectra of 0.8 mM [**1**$^{2+}$]Cl_2, in D_2O at pD 7 (λ_{exc} = 400 nm, 2 kHz,150 fs). This spectrum was vertically translated to account for baseline offset. TRIR kinetic plots of *rac*-[**1**$^{2+}$]Cl_2 (0.8 mM) recorded for (b) decay of the metal complex transient band at 1458 cm $^{-1}$ and (c) the recovery of nitrile bleach at 2240 cm^{-1} recorded in H_2O.

Cyclic voltammetry of 1^{2+} and dppz-10-CN

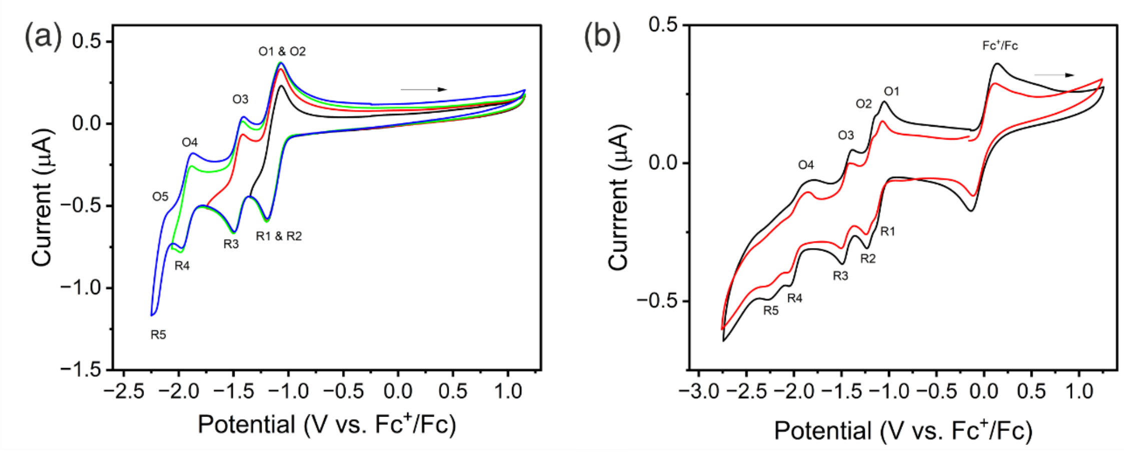

Figure S7. Cyclic voltammograms of 1 mM $\mathbf{1}^{2+}$ in DCM/10^{-1} M TBAH, Pt microdisk working electrode; (a) T = 293 K, v = 100 mV s^{-1}, and (b) T = 195 K, v = 50 mV s^{-1} (red curve) and = 100 mV s^{-1} (black curve).

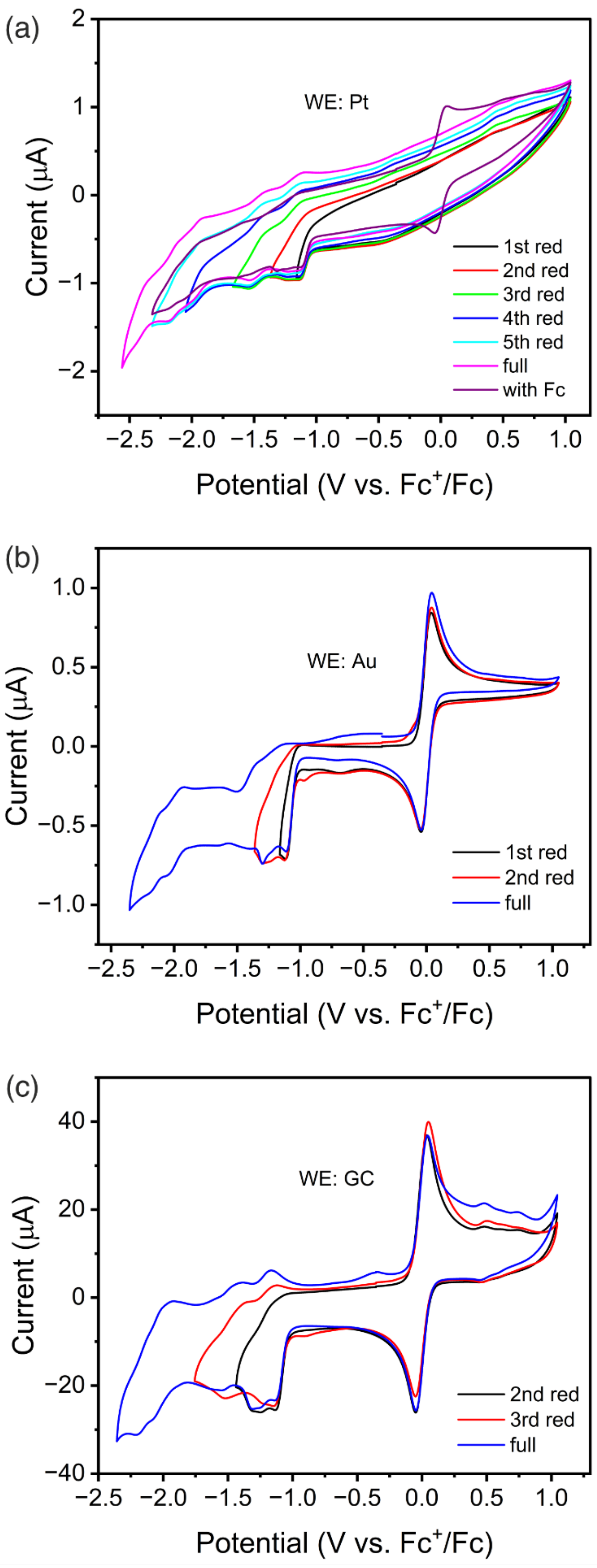

Figure S8. Cyclic voltammograms of 1 mM $\mathbf{1}^{2+}$ in PrCN/10^{-1} M TBAH, (a) Pt microdisk working electrode, (b) Au microdisk working electrode and (c) GC disk working electrode. T = 293 K, v = 100 mV s^{-1}.

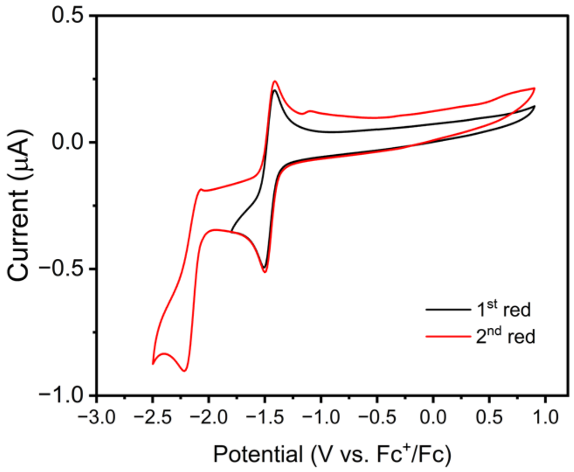

Figure S9. Cyclic voltammograms of 1 mM dppz-10-CN in DCM/10^{-1} M TBAH, Pt microdisk working electrode; T = 293 K, v = 100 mV s^{-1}.

Table S1. Redox potentials (E / V vs. Fc^+/Fc) of the studied compounds in DCM/TBAH.

Compound	Solvent	T [K]	$E_{1/2,1}$	$E_{1/2,2}$	$E_{1/2,3}$	$E_{1/2,4}$
dppz-10-CN	DCM	293	–1.46			–2.21[a]
**1**$^{2+}$	DCM	293	–1.14		–1.46	–1.93
		195	–1.10	–1.19	–1.44	–1.92

[a] The value corresponds to $E_{p,c}$.

Spectroelectrochemistry of 1^{2+} and dppz-10-CN

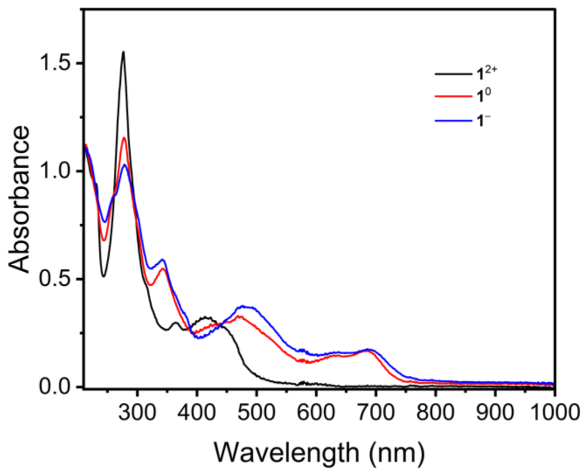

Figure S10. UV-Vis absorption spectra of $\mathbf{1}^{2+}$ (0.75 mM) and its 2e⁻ and 3e⁻-reduced forms in DCM/0.1 M TBAH in an OTTLE cell (Pt minigrid as WE) at T = 293 K.

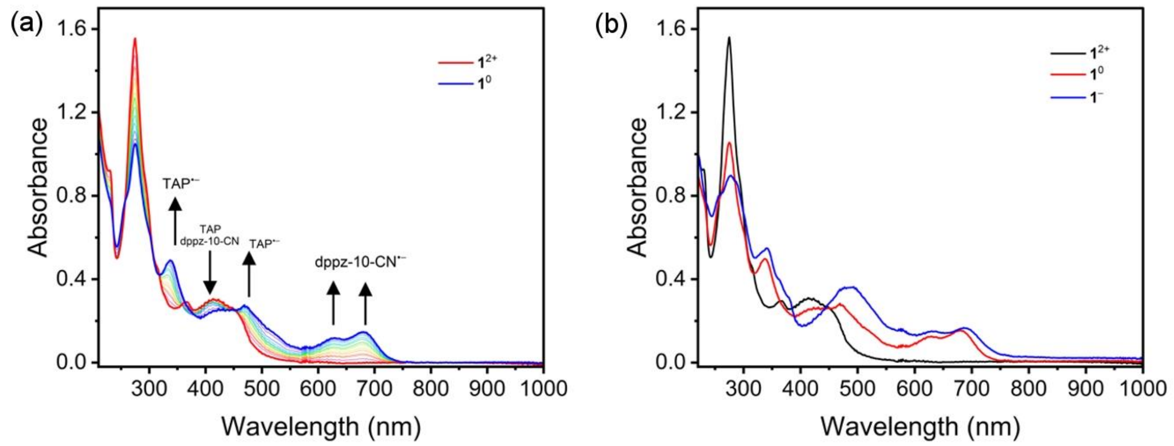

Figure S11. (a) UV-Vis spectral changes during the unresolved 2e reduction of $\mathbf{1}^{2+}$ (0.75 mM) in PrCN/10^{-1} M TBAH in an OTTLE cell (Pt minigrid as WE) at 293 K. (b) Comparison of the UV-Vis absorption spectra of parent $\mathbf{1}^{2+}$ at its 2e⁻ and 3e⁻-reduced forms.

The UV-Vis SEC spectral changes accompanying the electrochemical reduction of **1**$^{2+}$ reveal the characteristic intra-ligand absorption of reduced [dppz-10-CN]$^{•–}$ at 600–700 nm (Figures S10, S11 and S12(a)). This absorption appears in the TDDFT-calculated UV-Vis absorption spectra at about 620 nm (Figure S24). These reference data aid the assignment of the electronic absorption of the ligand [dppz-10-CN]$^{•–}$ in the reduced complexes (Figure 5 in the main text). The detailed assignment of electronic transitions in the UV–Vis absorption spectra of dppz-10-CN and [dppz-10-CN]$^{•–}$ (Figure S15) is given in Tables S2 and S3 linked to Figures S17 and S18, respectively. The corresponding IR SEC monitoring of the conversion of free dppz-10-CN to the corresponding radical anion in DCM was accompanied by the replacement of the parent ν(C≡N) absorption band at 2232 cm^{-1} with a new intense feature at 2195 cm^{-1} (Figure S12(b)). The absorbance was enhanced by a factor of four. These observations correlate with the DFT-calculated IR spectra (Figure S15), showing the same behavior and predicting even larger enhancement by a factor of six.

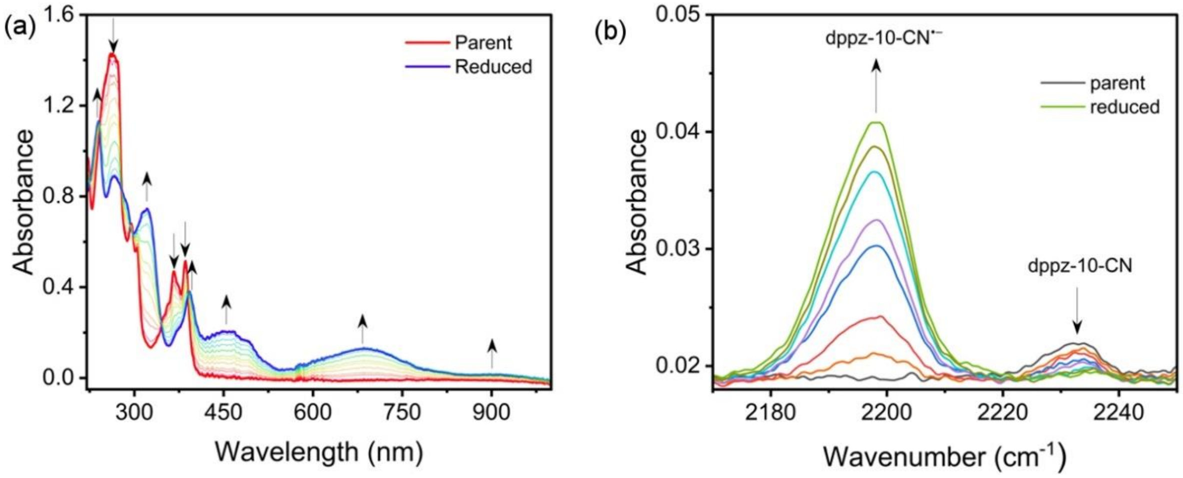

Figure S12. (a) UV-Vis SEC of dppz-10-CN (2mM/saturated) in DCM/0.2 M TBAH in an OTTLE cell (Pt as WE) at 293 K, TBAH (0.2 M) showing formation of the singly reduced [dppz-10-CN]$^{•–}$. (b) IR-SEC of dppz-10-CN (2mM/saturated) in DCM/0.2 M TBAH, Pt working electrode, at 293 K, showing the formation of singly reduced [dppz-10-CN]$^{•–}$.

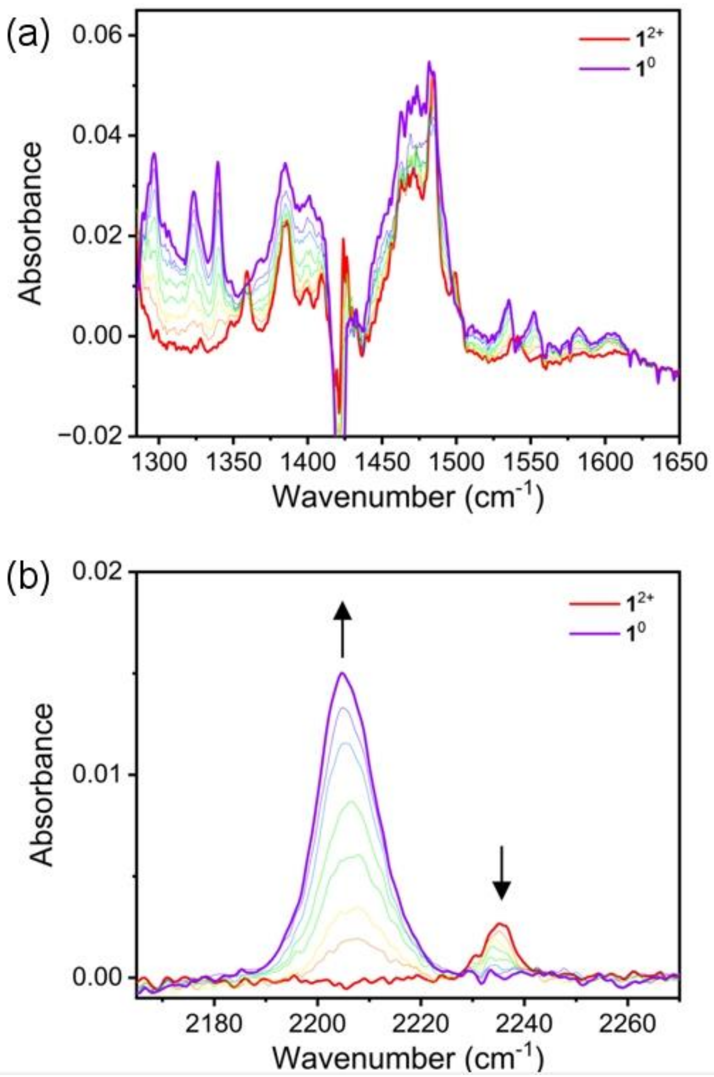

Figure S13. IR SEC of **1**$^{2+}$ (1.5 mM) in DCM/0.2 M TBAH, converting to neutral **1**^{0} at unresolved reduction waves R1 + R2. Experimental conditions: an OTTLE cell, Pt minigrid working electrode, *T* = 293 K.

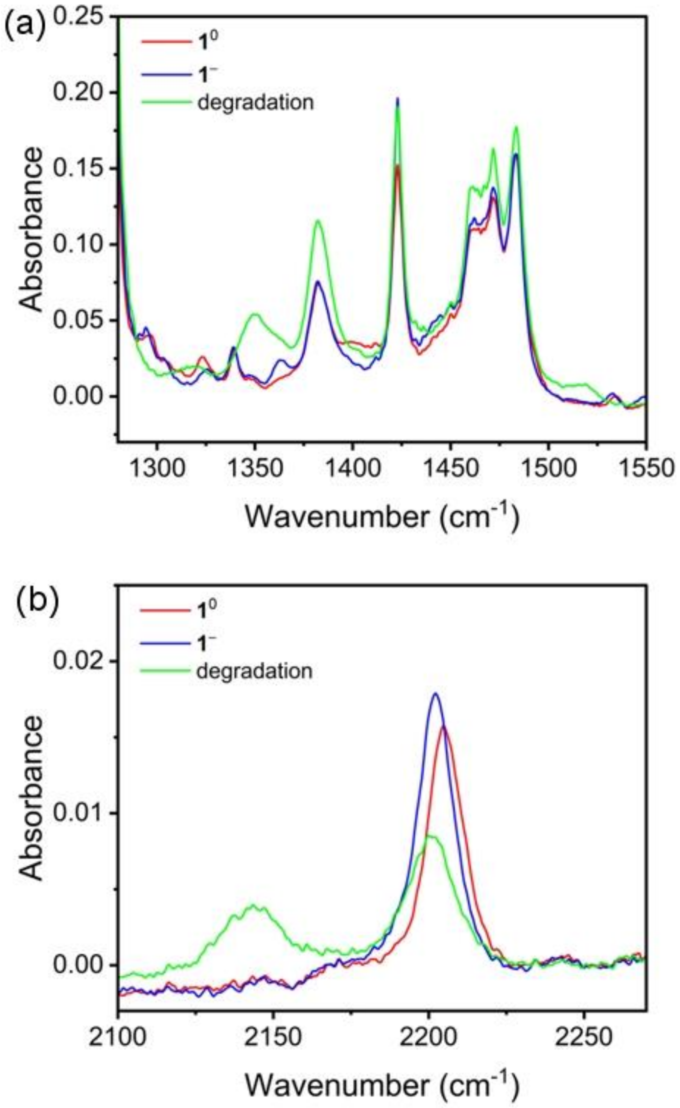

Figure S14. IR SEC of **1**^{0} (1.5 mM) in DCM/0.2 M TBAH further reducing to unstable **1**$^-$ at R3, Pt working electrode, at T = 293 K.

DFT and TDDFT calculations on free dppz-10-CN and its radical anion

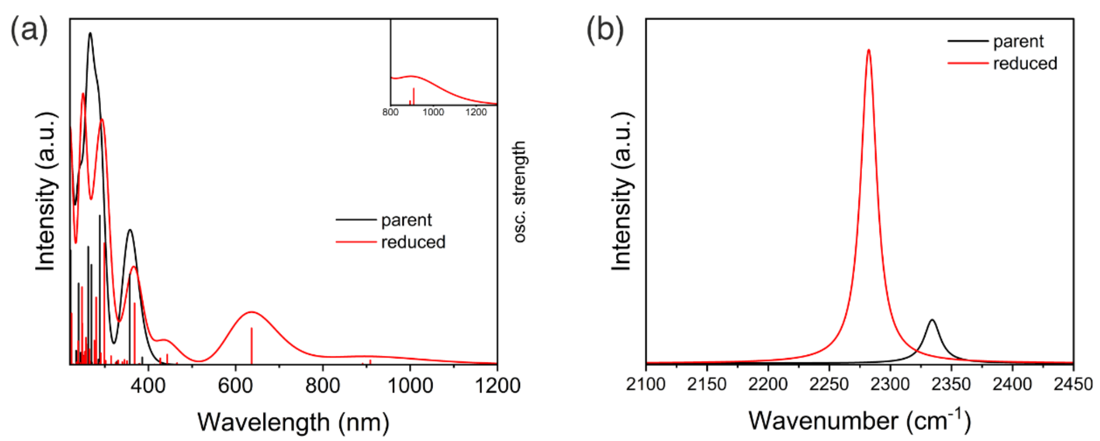

Figure S15. (a) TDDFT-calculated UV-Vis absorption spectra of dppz-10-CN (black) and [dppz-10-CN]•– (red)**.** (b) DFT-calculated IR spectra of dppz-10-CN (black) and [dppz-10-CN]•– (red).

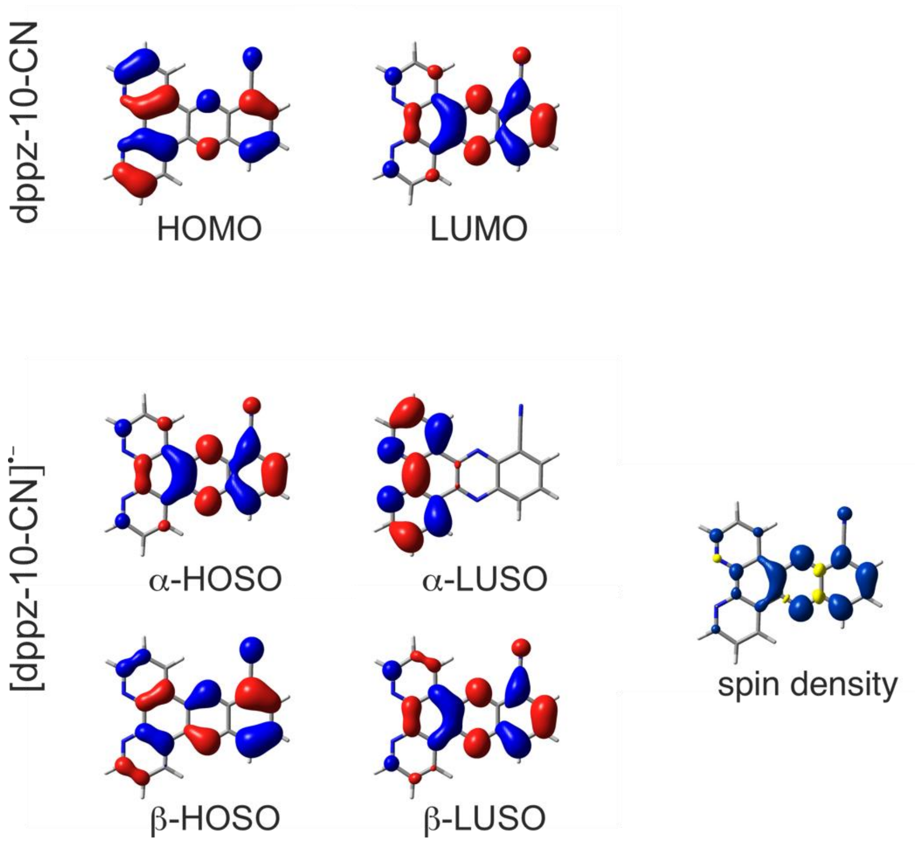

Figure S16. Frontier orbitals of dppz-10-CN and [dppz-10-CN]•–, and the spin density in [dppz-10-CN]•–.

Table S2. Major electronic excitations in dppz-10-CN determined by TD-DFT.

Wavelength (nm)	Osc. strength (*f*)	Major contributions (%)
**386**	0.032	HOMO → LUMO (70)
**357**	0.392	HOMO-1 → LUMO (67)
**289**	0.646	HOMO-1 → LUMO+2 (32)<br>HOMO → LUMO+1 (59)
**269**	0.433	HOMO-7 → LUMO (20)<br>HOMO-3 → LUMO+1 (30)<br>HOMO-1 → LUMO+2 (48)<br>HOMO → LUMO+1 (30)
**262**	0.511	HOMO-6 → LUMO (32)<br>HOMO-1 → LUMO+1 (46)<br>HOMO → LUMO+2 (37)
**248**	0.118	HOMO-7 → LUMO (46)<br>HOMO-1 → LUMO+2 (22)<br>HOMO → LUMO+3 (41)
**240**	0.352	HOMO-3 → LUMO+2 (67)
**222**	0.496	HOMO-3 → LUMO+3 (61)
**218**	0.158	HOMO-6 → LUMO+1 (47)<br>HOMO → LUMO+4 (47)

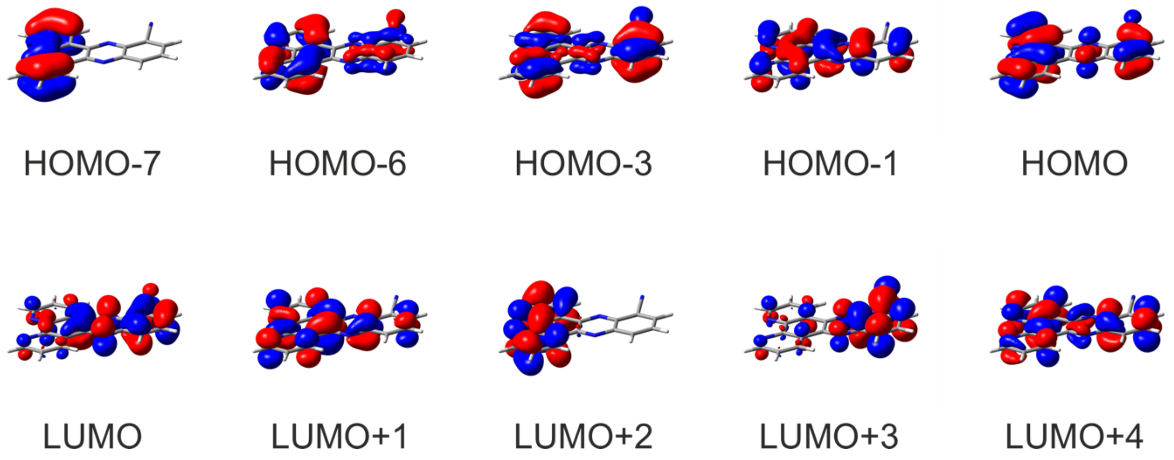

Figure S17. MOs involved in electronic transitions in dppz-10-CN (listed in Table S2).

Table S3. Major electronic excitations in $[\text{dppz-10-CN}]^{\bullet-}$ determined by TD-DFT.

Wavelength (nm)	Osc. strength (*f*)	Major contributions (%)
**908**	0.019	α-HOSO → α-LUSO+1 (98)
**636**	0.158	α-HOSO → α-LUSO+2 (98)
**368**	0.266	α-HOSO-1 → α-LUSO+1 (25)<br>β-HOSO-1 → β-LUSO (92)
**299**	0.526	α-HOSO-5 → α-LUSO+1 (33)<br>α-HOSO-2 → α-LUSO (31)<br>α-HOSO-1 → α-LUSO+1 (59)<br>β-HOSO-1 → β-LUSO (20)<br>β-HOSO-1 → β-LUSO+1 (20)<br>β-HOSO → β-LUSO+2 (49)
**280**	0.291	α-HOSO-5 → α-LUSO+1 (49)<br>α-HOSO-2 → α-LUSO (47)<br>α-HOSO-1 → α-LUSO+1 (46)<br>β-HOSO-3 → β-LUSO+2 (23)<br>β-HOSO-1 → β-LUSO+2 (23)
**276**	0.104	α-HOSO-2 → α-LUSO+1 (54)<br>α-HOSO → α-LUSO+10 (30)<br>β-HOSO-3 → β-LUSO+1 (36)<br>β-HOSO-1 → β-LUSO+2 (50)
**257**	0.117	α-HOSO-7 → α-LUSO (22)<br>α-HOSO-5 → α-LUSO+2 (28)<br>α-HOSO-1 → α-LUSO+2 (28)<br>β-HOSO-6 → β-LUSO+1 (32)<br>β-HOSO-3 → β-LUSO+1 (41)<br>β-HOSO-3 → β-LUSO+2 (28)
**249**	0.179	α-HOSO-5 → α-LUSO (29)<br>α-HOSO-5 → α-LUSO+1 (28)<br>α-HOSO-1 → α-LUSO+4 (23)<br>α-HOSO → α-LUSO+10 (22)<br>β-HOSO-8 → β-LUSO (37)<br>β-HOSO-6 → β-LUSO (24)<br>β-HOSO-3 → β-LUSO+1 (23)<br>β-HOSO-1 → β-LUSO+3 (34)<br>β-HOSO → β-LUSO+4 (31)
**248**	0.336	α-HOSO-5 → α-LUSO (37)<br>α-HOSO-2 → α-LUSO+1 (20)<br>α-HOSO-1 → α-LUSO+2 (20)<br>β-HOSO-8 → β-LUSO (26)<br>β-HOSO-6 → β-LUSO (49)<br>β-HOSO-3 → β-LUSO+1 (21)<br>β-HOSO-3 → β-LUSO+3 (23)<br>β-HOSO-1 → β-LUSO+3 (24)
**239**	0.102	α-HOSO-2 → α-LUSO+2 (27)<br>β-HOSO-8 → β-LUSO (27)<br>β-HOSO-3 → β-LUSO+2 (35)<br>β-HOSO-1 → β-LUSO+3 (33)<br>β-HOSO → β-LUSO+4 (62)

Wavelength (nm)	Osc. strength (*f*)	Major contributions (%)
**224**	0.222	α-HOSO-5 → α-LUSO+2 (31)<br>α-HOSO-1 → α-LUSO+4 (23)<br>α-HOSO → α-LUSO+14 (21)<br>β-HOSO-10 → β-LUSO (48)<br>β-HOSO-9 → β-LUSO+2 (24)<br>β-HOSO-6 → β-LUSO+1 (23)<br>β-HOSO-3 → β-LUSO+3 (32)<br>β-HOSO → β-LUSO+5 (35)
**220**	0.157	α-HOSO-7 → α-LUSO (25)<br>α-HOSO-5 → α-LUSO+2 (33)<br>β-HOSO-10 → β-LUSO (30)<br>β-HOSO-8 → β-LUSO+1 (25)<br>β-HOSO-3 → β-LUSO+3 (57)<br>β-HOSO → β-LUSO+5 (41)
**218**	0.268	α-HOSO-7 → α-LUSO+1 (21)<br>α-HOSO-1 → α-LUSO+3 (25)<br>α-HOSO-1 → α-LUSO+4 (49)<br>β-HOSO-9 → β-LUSO (21)<br>β-HOSO-6 → β-LUSO+2 (43)<br>β-HOSO → β-LUSO+4 (34)<br>β-HOSO → β-LUSO+6 (22)

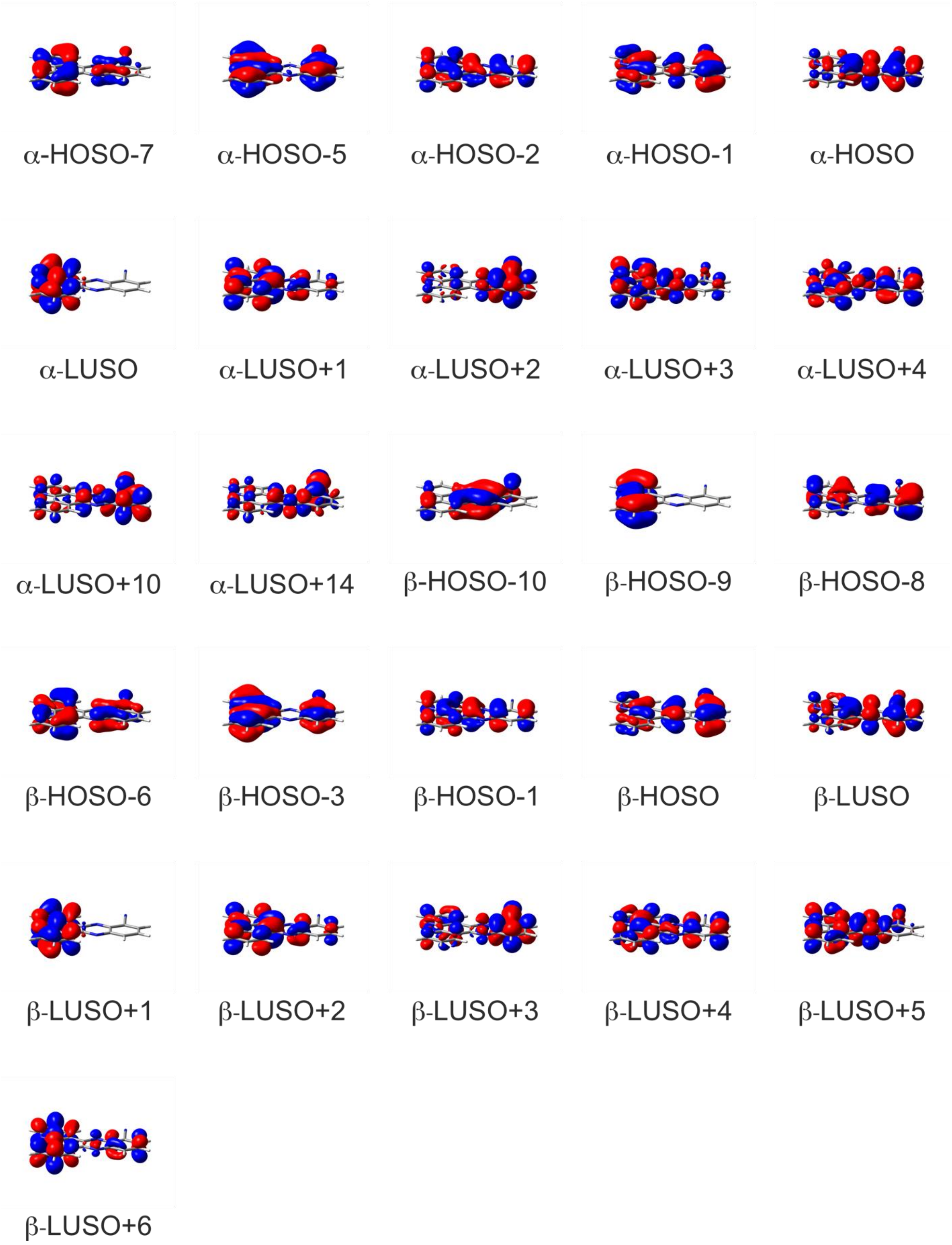

Figure S18. MOs involved in electronic transitions in [dppz-10-CN]$^{\bullet-}$ (listed in Table S3).

DFT and TDDFT calculations on 1^{2+} and its reduced forms

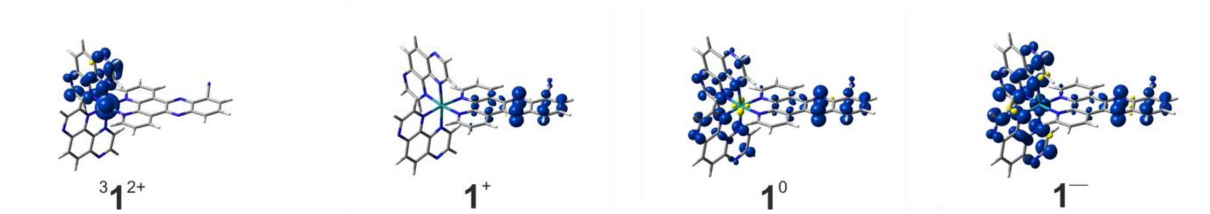

Figure S19. Distribution of spin densities in **1**$^{2+}$ (in the lowest ^{3}MLCT state) and the 1e^{-}-reduced (at R1; doublet) **1**$^{+}$, 2e^{-}-reduced (at R2; triplet) **1**^{0} and 3e^{-}-reduced (at R3; quadruplet) **1**$^{-}$.

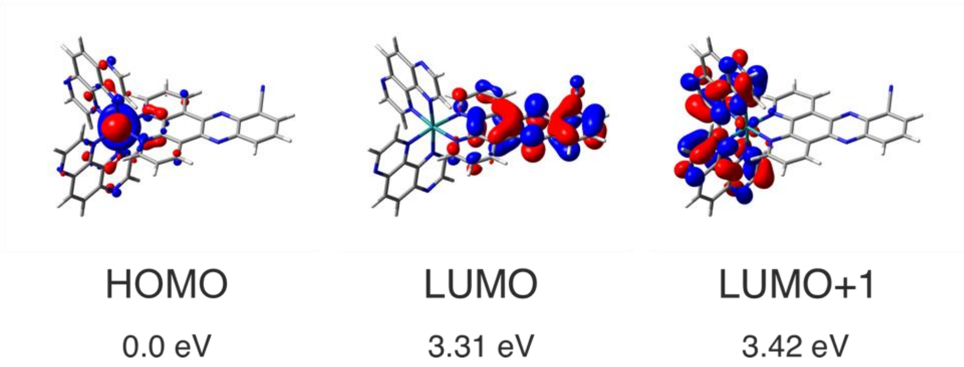

Figure S20. Frontier MOs of **1**$^{2+}$ and their relative energies, with impact on the poorly resolved reduction steps R1 (dppz-10-CN-localized) and R2 (TAP-localized).

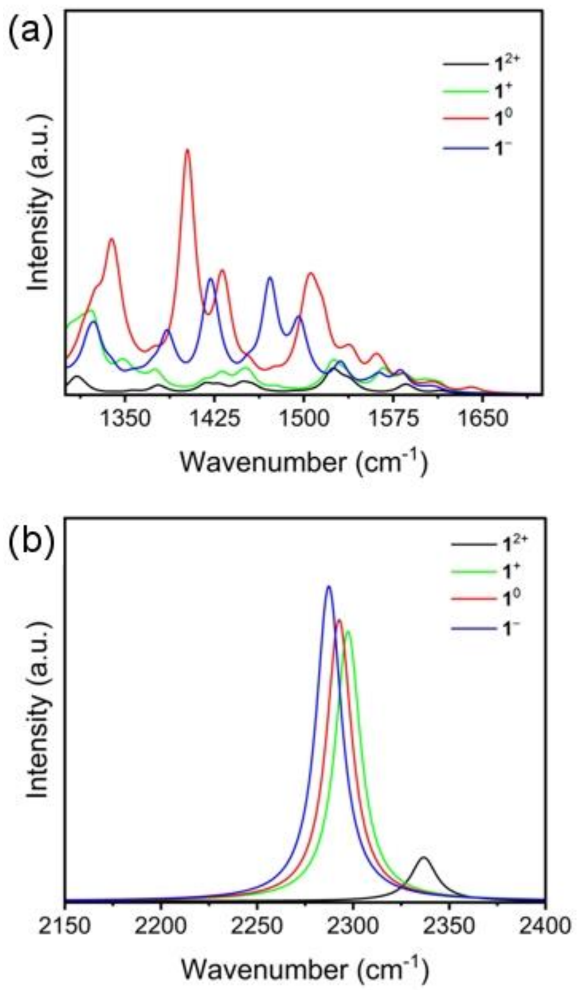

Figure S21. Calculated IR spectra of $\mathbf{1}^{2+}$ (black line), $\mathbf{1}^{+}$ (green line), $\mathbf{1}^{0}$ (red line) and $\mathbf{1}^{-}$ (blue line).

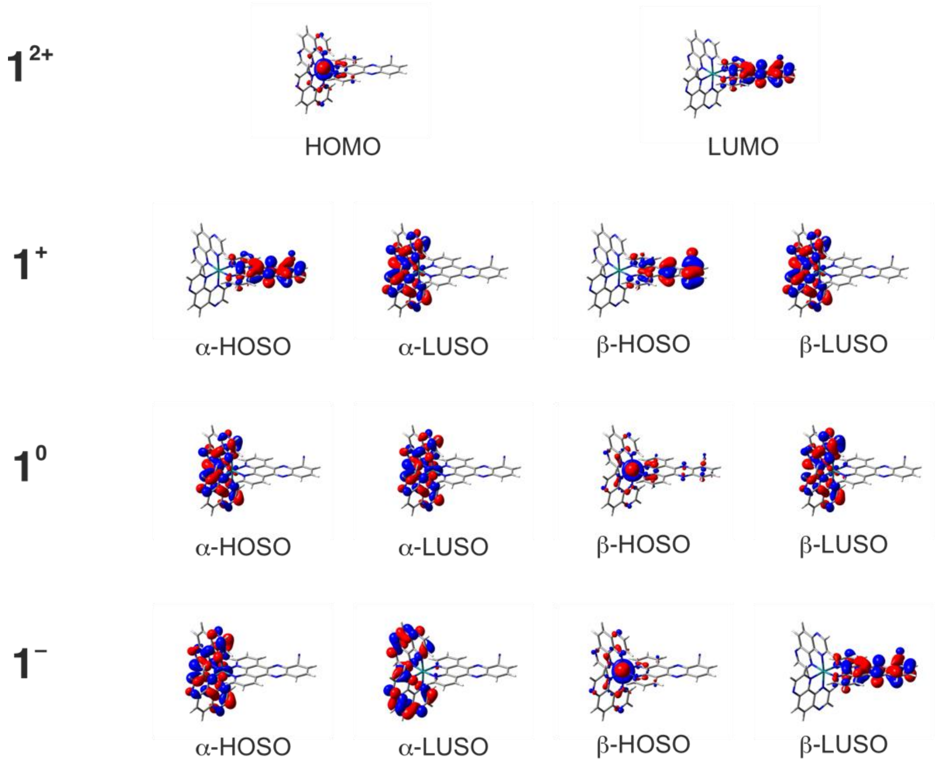

Figure S22. Frontier orbitals of $\mathbf{1}^{n}$ (n = 2+, 1+, 0 and 1–). TDDFT optimizations were performed for the state localized on the dppz part (^{1}MLCT). The outcome consistently results in a TAP-localized ^{3}MLCT state when attempting to achieve the energetic minimum of the ^{3}MLCT localized on dppz (see Table S4 below).

Table S4 Comparison of energies of the low-lying MLCT states of $\mathbf{1}^{2+}$.

state	localization	energy / eV	Δ*E* / eV		Im. freq.
GS	-	–62739.22	0.0000		no
^{3}MLCT	TAP	–62737.26	1.9521	0.0000	no
^{3}MLCT	dppz-10-CN	–62736.85	2.3706	0.4185	yes
^{1}MLCT	dppz-10-CN	–62736.39	2.8294	0.8773	yes

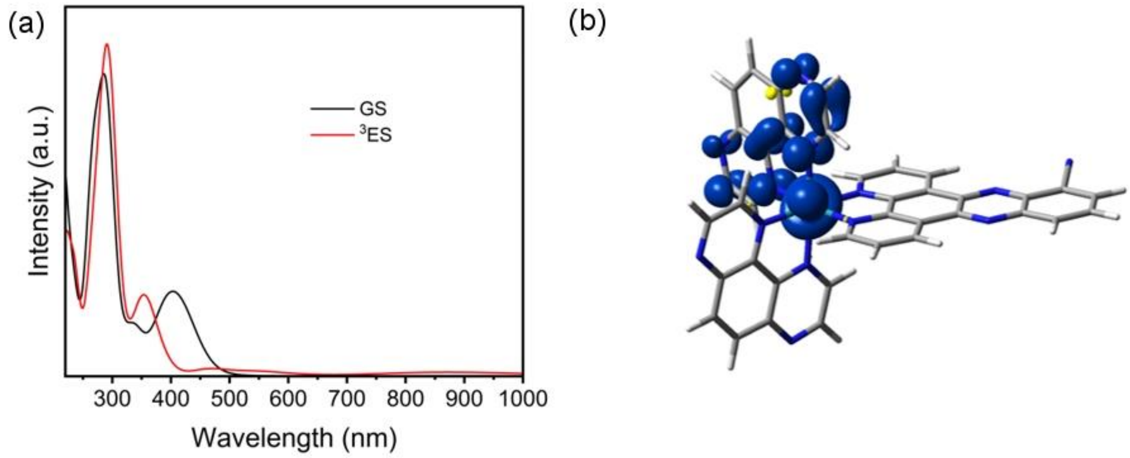

Figure S23. (a) Calculated UV-Vis absorption spectra of **1**$^{2+}$ in the ground state (black line) and the TAP-based ^{3}MLCT excited state (red line). (b) Spin density in the TAP-based lowest ^{3}MLCT excited state of **1**$^{2+}$.

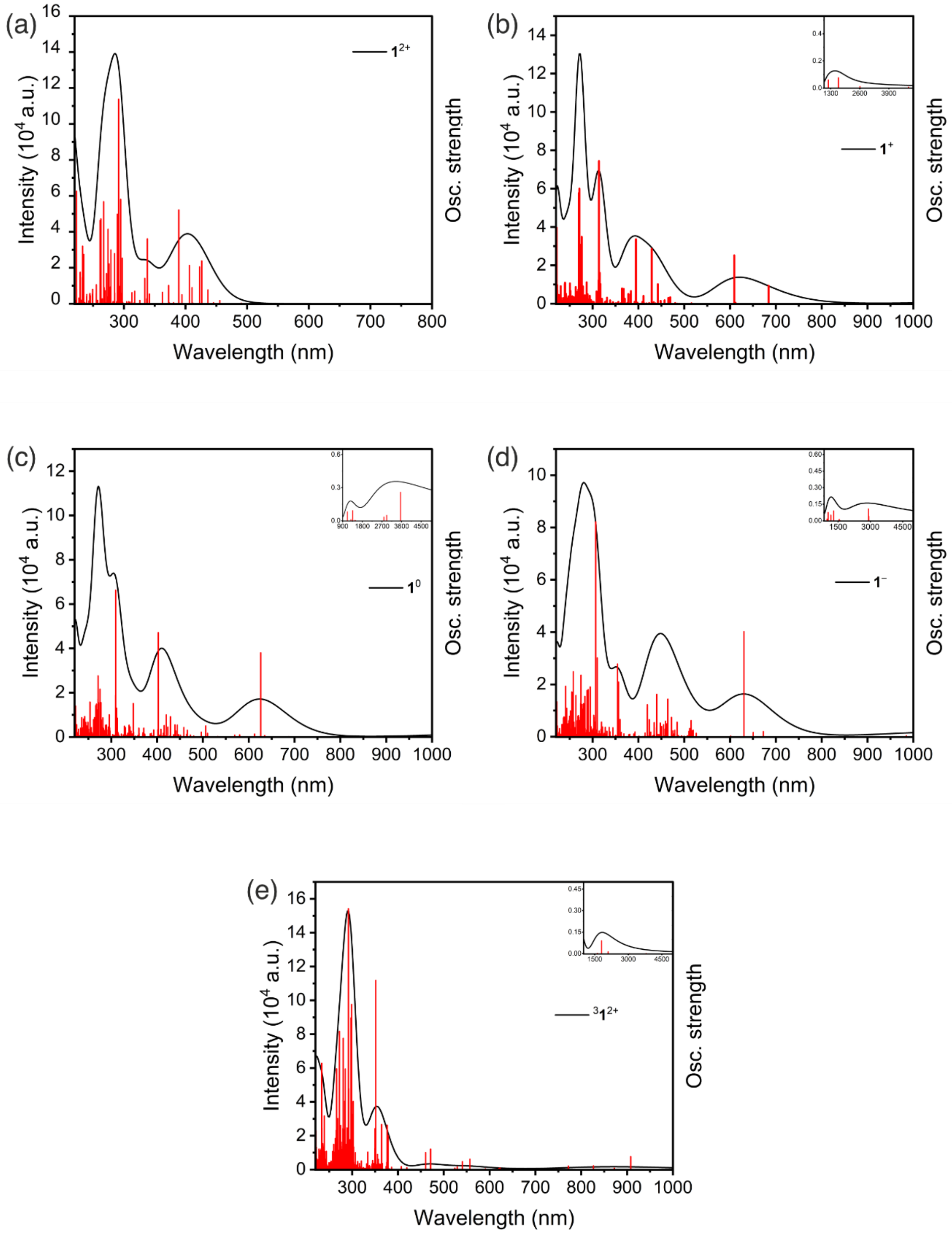

Figure S24. TDDFT-calculated UV-Vis absorption spectra of (a) **1**$^{2+}$ (GS) (b) **1**$^{+}$, (c) **1**^{0} (d) **1**$^{-}$ and (e) **1**$^{2+}$ (in the lowest ^{3}MLCT state), with vertical transitions (red) as listed in Tables S5–S9.

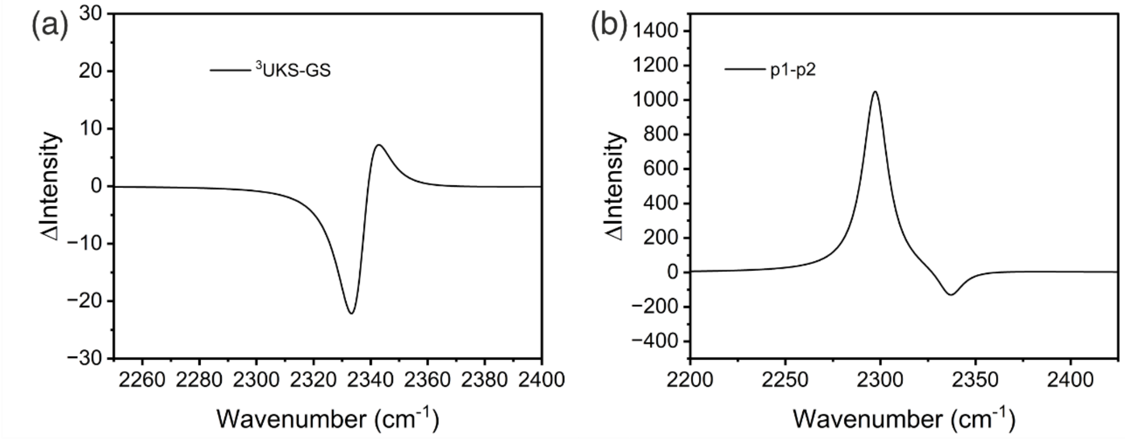

Figure S25. Calculated difference absorbance IR spectra of **1**$^{2+}$ in the lowest ^{3}MLCT state (left) and **1**$^{+}$ (right), showing the bleached GS **1**$^{2+}$.

Table S5. Major electronic excitations in $\mathbf{1}^{2+}$ determined by TD-DFT.

Wavelength (nm)	Osc. strength (*f*)	Major contributions (%)
**426**	0.120	HOMO-1 → LUMO (66)
**423**	0.103	HOMO-2 → LUMO+1 (30)<br>HOMO-1 → LUMO+3 (32)<br>HOMO → LUMO+4 (52)
**406**	0.107	HOMO-1 → LUMO+3 (47)<br>HOMO→ LUMO+5 (41)
**389**	0.262	HOMO-2 → LUMO+2 (31)<br>HOMO-2 → LUMO+3 (31)<br>HOMO-1 → LUMO+4 (20)<br>HOMO-1 → LUMO+5 (42)
**338**	0.181	HOMO-4 → LUMO (58)<br>HOMO-3 → LUMO+6 (28)<br>HOMO-2 → LUMO+6 (21)
**297**	0.128	HOMO-4 → LUMO+4 (65)
**295**	0.291	HOMO-4 → LUMO+5 (50)<br>HOMO-3 → LUMO+6 (32)
**291**	0.569	HOMO-9 → LUMO (39)<br>HOMO-4 → LUMO (20)<br>HOMO-4 → LUMO+5 (32)<br>HOMO-3 → LUMO+6 (40)
**289**	0.250	HOMO-9 → LUMO (51)<br>HOMO-4 → LUMO+5 (21)<br>HOMO-3 → LUMO+6 (28)
**285**	0.140	HOMO-10 → LUMO+4 (31)<br>HOMO-9 → LUMO+3 (43)<br>HOMO-2 → LUMO+7 (30)
**279**	0.150	HOMO-9 → LUMO+2 (24)<br>HOMO-8 → LUMO+4 (22)<br>HOMO-5 → LUMO+5 (31)<br>HOMO-2 → LUMO+7 (36)
**276**	0.111	HOMO-10 → LUMO+4 (36)<br>HOMO-9 → LUMO+3 (30)<br>HOMO-8 → LUMO+5 (22)<br>HOMO-4 → LUMO+6 (23)
**274.3**	0.124	HOMO-10 → LUMO+4 (38)<br>HOMO-2 → LUMO+7 (37)
**274.2**	0.208	HOMO-10 → LUMO+2 (29)<br>HOMO-6 → LUMO+4 (20)<br>HOMO-1 → LUMO+7 (26)<br>HOMO → LUMO+9 (32)
**267**	0.284	HOMO-1 → LUMO+9 (53)<br>HOMO → LUMO+8 (27)
**263**	0.236	HOMO-10 → LUMO+5 (46)<br>HOMO-8 → LUMO+5 (36)<br>HOMO-4 → LUMO+6 (20)
**261**	0.232	HOMO-10 → LUMO+5 (47)<br>HOMO-8 → LUMO+5 (29)<br>HOMO-4 → LUMO+6 (25)
**235**	0.138	HOMO-15 → LUMO (37)<br>HOMO-3 → LUMO+8 (43)
**233**	0.161	HOMO-18 → LUMO (30)<br>HOMO-3 → LUMO+10 (30)<br>HOMO-1 → LUMO+13 (29)
**223**	0.313	HOMO-5 → LUMO+7 (62)

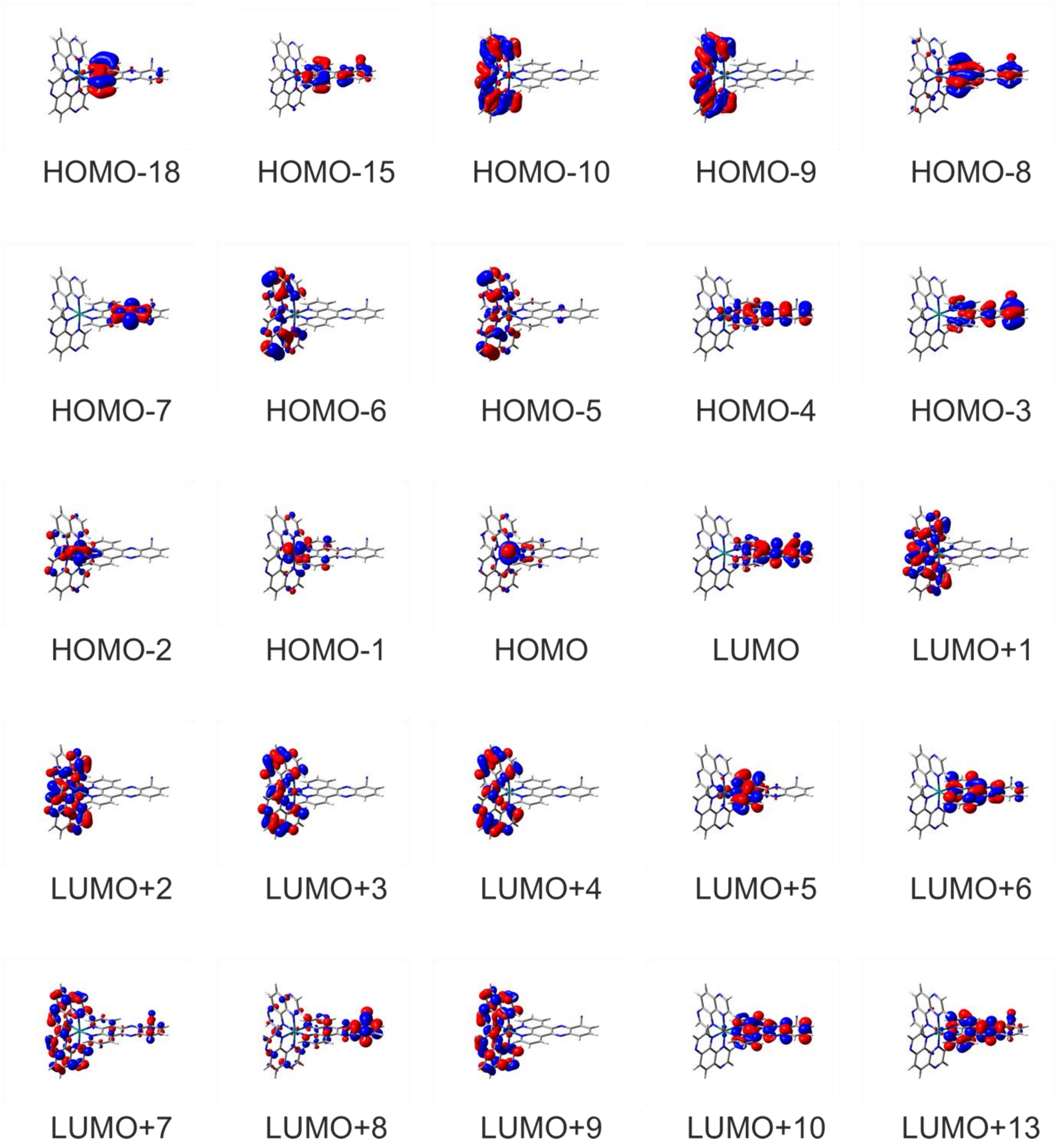

Figure S26. MOs involved in the electronic transitions in **1**$^{2+}$ (listed in Table S5).

Table S6. Major electronic excitations in **1**⁺ determined by TD-DFT.

**Wavelength (nm)**	**Osc. strength (*f*)**	**Major contributions (%)**
**1644**	0.015	α-HOSO → α-LUSO+4 (99.5)
**1194**	0.012	α-HOSO → α-LUSO+5 (99)
**684**	0.064	α-HOSO → α-LUSO+6 (98)<br>α-HOSO → α-LUSO+8 (21)
**609**	0.169	α-HOSO → α-LUSO+6 (21)<br>α-HOSO → α-LUSO+8 (93)
**442**	0.069	α-HOSO-4 → α-LUSO (31)<br>α-HOSO-3 → α-LUSO+2 (37)<br>α-HOSO-2 → α-LUSO+3 (26)<br>α-HOSO-1 → α-LUSO+3 (22)<br>β-HOSO-4 → β-LUSO (26)<br>β-HOSO-2 → β-LUSO+2 (34)<br>β-HOSO-2 → β-LUSO+3 (21)<br>β-HOSO-1 → β-LUSO+2 (23)<br>β-HOSO-1 → β-LUSO+3 (31)<br>β-HOSO → β-LUSO+3 (22)
**429**	0.192	α-HOSO-4 → α-LUSO (25)<br>α-HOSO-3 → α-LUSO+1 (21)<br>α-HOSO-3 → α-LUSO+2 (50)<br>α-HOSO-3 → α-LUSO+3 (23)<br>α-HOSO-2 → α-LUSO+3 (25)<br>β-HOSO-4 → β-LUSO (21)<br>β-HOSO-2 → β-LUSO+1 (21)<br>β-HOSO-2 → β-LUSO+2 (38)<br>β-HOSO-2 → β-LUSO+3 (27)<br>β-HOSO-1 → β-LUSO+3 (25)
**395**	0.225	α-HOSO-4 → α-LUSO+1 (37)<br>α-HOSO-4 → α-LUSO+2 (22)<br>α-HOSO-3 → α-LUSO+4 (23)<br>α-HOSO-1 → α-LUSO+2 (24)<br>β-HOSO-5 → β-LUSO (34)<br>β-HOSO-4 → β-LUSO+1 (26)<br>β-HOSO-3 → β-LUSO+1 (28)<br>β-HOSO-2 → β-LUSO (29)<br>β-HOSO-2 → β-LUSO+4 (22)
**316**	0.109	α-HOSO-14 → α-LUSO (23)<br>α-HOSO-5 → α-LUSO+5 (45)<br>α-HOSO-4 → α-LUSO+5 (23)<br>β-HOSO-14 → β-LUSO (21)<br>β-HOSO-3 → β-LUSO+6 (26)<br>β-HOSO-1 → β-LUSO+6 (27)
**314**	0.497	α-HOSO-5 → α-LUSO+5 (33)<br>α-HOSO-2 → α-LUSO+5 (39)<br>α-HOSO-1 → α-LUSO+5 (43)<br>α-HOSO → α-LUSO+20 (22)<br>β-HOSO-5 → β-LUSO+4 (27)<br>β-HOSO → β-LUSO+6 (36)

Wavelength (nm)	Osc. strength (*f*)	Major contributions (%)
**276**	0.234	α-HOSO-11 → α-LUSO+1 (37)<br>α-HOSO-9 → α-LUSO+3 (20)<br>α-HOSO-1 → α-LUSO+6 (24)<br>β-HOSO-10 → β-LUSO+1 (37)<br>β-HOSO-1 → β-LUSO+8 (21)
**275**	0.206	α-HOSO-7 → α-LUSO+4 (34)<br>α-HOSO-6 → α-LUSO+5 (29)<br>α-HOSO-3 → α-LUSO+7 (25)<br>β-HOSO-9 → β-LUSO+1 (20)<br>β-HOSO-6 → β-LUSO+5 (29)<br>β-HOSO-5 → β-LUSO+6 (21)<br>β-HOSO-2 → β-LUSO+8 (23)
**271**	0.401	α-HOSO-15 → α-LUSO (23)<br>α-HOSO-14 → α-LUSO (22)<br>α-HOSO-14 → α-LUSO+1 (34)<br>α-HOSO-7 → α-LUSO+4 (27)<br>α-HOSO-6 → α-LUSO+5 (20)<br>β-HOSO-15 → β-LUSO (21)<br>β-HOSO-13 → β-LUSO (20)<br>β-HOSO-13 → β-LUSO+1 (24)<br>β-HOSO-12 → β-LUSO+1 (20)<br>β-HOSO-6 → β-LUSO+5 (26)
**270.3**	0.123	β-HOSO-11 → β-LUSO+1 (83)
**270.0**	0.386	α-HOSO-15 → α-LUSO (20)<br>α-HOSO-12 → α-LUSO+1 (21)<br>β-HOSO-14 → β-LUSO (23)<br>β-HOSO-14 → β-LUSO+1 (27)<br>β-HOSO-11 → β-LUSO+1 (44)
**221**	0.265	α-HOSO-8 → α-LUSO+6 (54)<br>α-HOSO-5 → α-LUSO+10 (24)<br>β-HOSO-7 → β-LUSO+7 (53)

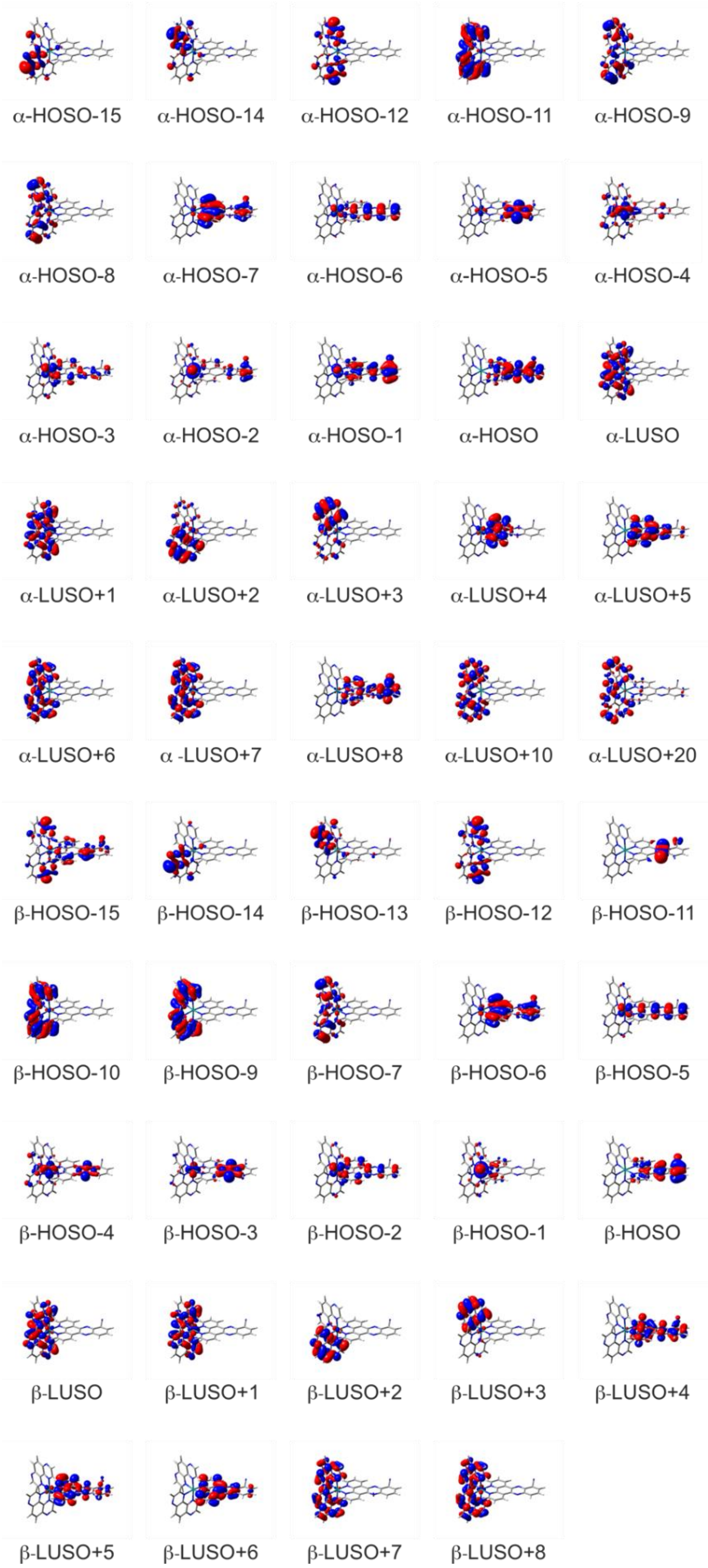

Figure S27. MOs involved in the electronic transitions in **1**$^+$ (listed in Table S6).

Table S7. Major electronic excitations of **1**^{0} determined by TD-DFT.

Wavelength (nm)	Osc. strength (*f*)	Major contributions (%)
**3553**	0.04	α-HOSO → α-LUSO (102)
**2919**	0.008	α-HOSO → α-LUSO+1 (90)<br>α-HOSO → α-LUSO+2 (42)
**1360**	0.015	α-HOSO-1 → α-LUSO+1 (20)<br>α-HOSO-1 → α-LUSO+2 (48)<br>α-HOSO-1→ α-LUSO+3 (84)
**1117**	0.013	α-HOSO-1 → α-LUSO+4 (98)
**626**	0.234	α-HOSO-1 → α-LUSO+5 (68)<br>α-HOSO-1 → α-LUSO+6 (70)
**403**	0.290	α-HOSO-3 → α-LUSO+3 (38)<br>β-HOSO-3 → β-LUSO+3 (26)<br>β-HOSO-2 → β-LUSO+4 (58)<br>β-HOSO-2 → β-LUSO+5 (26)<br>β-HOSO-1 → β-LUSO (22)
**310**	0.407	α-HOSO-5 → α-LUSO+4 (46)<br>α-HOSO-1→ α-LUSO+17 (27)<br>α-HOSO-1 → α-LUSO+18 (27)<br>β-HOSO-13 → β-LUSO (20)<br>β-HOSO-5 → β-LUSO+4 (22)<br>β-HOSO-1 → β-LUSO+6 (31)
**309**	0.115	α-HOSO-16 → α-LUSO (24)<br>α-HOSO-16 → α-LUSO+1 (26)<br>α-HOSO-5 → α-LUSO+4 (30)<br>β-HOSO-14 → β-LUSO+1 (27)<br>β-HOSO-13 → β-LUSO (23)<br>β-HOSO-11 → β-LUSO (27)<br>β-HOSO-5 → β-LUSO+4 (23)<br>β-HOSO-1 → β-LUSO+6 (20)
**275**	0.133	α-HOSO-12 → α-LUSO (21)<br>α-HOSO-2 → α-LUSO+7 (21)<br>β-HOSO-10 → β-LUSO+3 (29)<br>β-HOSO-8 → β-LUSO+4 (29)<br>β-HOSO-3 → β-LUSO+8 (24)<br>β-HOSO → β-LUSO+8 (38)
**271.3**	0.170	α-HOSO-9 → α-LUSO+3 (34)<br>α-HOSO-3 → α-LUSO+7 (25)<br>β-HOSO-9 → β-LUSO+4 (22)<br>β-HOSO-2 → β-LUSO+8 (34)<br>β-HOSO-1 → β-LUSO+7 (38)
**271.0**	0.105	α-HOSO-9 → α-LUSO+3 (30)<br>α-HOSO-8 → α-LUSO+3 (44)<br>α-HOSO-4 → α-LUSO+6 (21)<br>β-HOSO-6 → β-LUSO+5 (27)<br>β-HOSO-1 → β-LUSO+7 (38)
**219**	0.143	α-HOSO-20 → α-LUSO+1 (25)<br>α-HOSO-9 → α-LUSO+5 (33)<br>α-HOSO-8 → α-LUSO+6 (21)<br>α-HOSO-5 → α-LUSO+9 (22)<br>β-HOSO-8 → β-LUSO+7 (25)<br>β-HOSO-7 → β-LUSO+7 (44)<br>β-HOSO-6 → β-LUSO+7 (20)

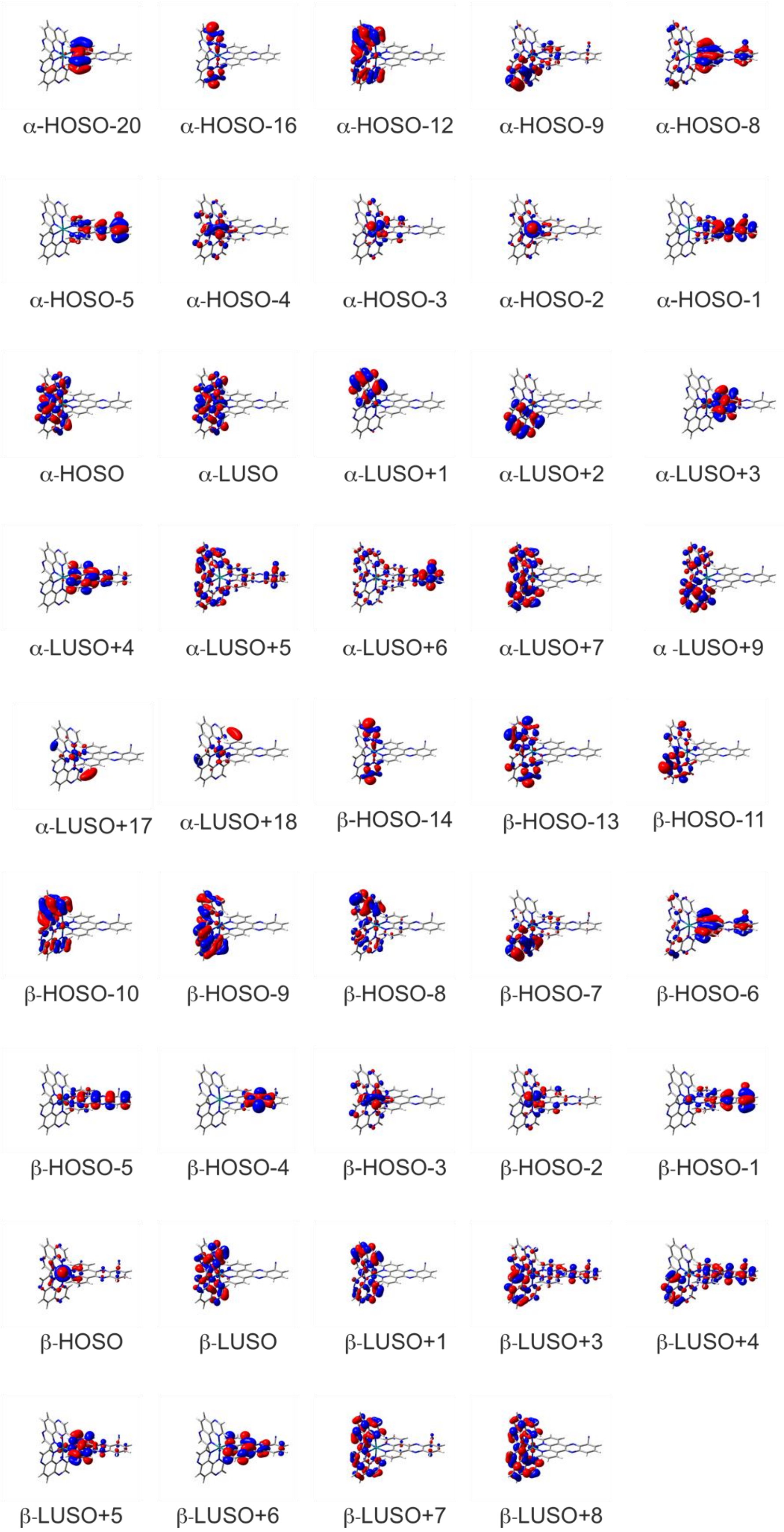

Figure S28. MOs involved in the electronic transitions of $\mathbf{1}^0$ (listed in Table S7).

Table S8. Major electronic excitations in **1**⁻ determined by TD-DFT.

Wavelength (nm)	Osc. strength (*f*)	Major contributions (%)
**2915**	0.017	α-HOSO-1 → α-LUSO+1 (56)<br>α-HOSO → α-LUSO (80)
**1300**	0.014	α-HOSO-1 → α-LUSO+2 (98)
**1044**	0.012	α-HOSO-2 → α-LUSO+3 (97)
**673**	0.011	α-HOSO-1 → α-LUSO+6 (42)<br>α-HOSO → α-LUSO+4 (49)<br>α-HOSO → α-LUSO+5 (74)
**630**	0.220	α-HOSO-2 → α-LUSO+4 (96)
**464**	0.079	α-HOSO-4 → α-LUSO (41)<br>α-HOSO-4 → α-LUSO+2 (23)<br>α-HOSO-3 → α-LUSO+1 (21)<br>α-HOSO-2 → α-LUSO+5 (49)<br>α-HOSO-2 → α-LUSO+7 (31)<br>α-HOSO-2 → α-LUSO+8 (25)<br>β-HOSO-1 → β-LUSO+5 (38)<br>β-HOSO → β-LUSO+3 (21)
**439**	0.089	α-HOSO-4 → α-LUSO+1 (39)<br>β-HOSO-2 → β-LUSO+2 (22)<br>β-HOSO-2 → β-LUSO+3 (72)<br>β-HOSO-1 → β-LUSO+2 (25)<br>β-HOSO-1 → β-LUSO+3 (23)
**357**	0.114	β-HOSO-6 → β-LUSO (55)<br>β-HOSO-4 → β-LUSO (60)<br>β-HOSO-3 → β-LUSO+6 (44)
**354**	0.152	α-HOSO-6 → α-LUSO+3 (47)<br>α-HOSO-2 → α-LUSO+12 (23)<br>β-HOSO-6 → β-LUSO (20)<br>β-HOSO-4 → β-LUSO (24)<br>β-HOSO-3 → β-LUSO+6 (60)
**310**	0.165	α-HOSO-6 → -αLUSO+3 (21)<br>α-HOSO-4 → α-LUSO+4 (61)<br>α-HOSO-4 → α-LUSO+5 (20)<br>β-HOSO-3 → β-LUSO+6 (33)<br>β-HOSO-2 → β-LUSO+7 (44)
**307**	0.448	α-HOSO-8 → α-LUSO+2 (23)<br>α-HOSO-6 → α-LUSO+3 (51)<br>α-HOSO-4 → α-LUSO+4 (52)<br>β-HOSO-3 → β-LUSO+6 (29)
**295**	0.104	α-HOSO-12 → α-LUSO (31)<br>α-HOSO-11 → α-LUSO+1 (28)<br>α-HOSO-10 → α-LUSO+1 (22)<br>α-HOSO-8 → α-LUSO+1 (31)<br>β-HOSO-6 → β-LUSO+3 (24)<br>β-HOSO-4 → β-LUSO+3 (51)<br>β-HOSO-1 → β-LUSO+7 (21)

Wavelength (nm)	Osc. strength (*f*)	Major contributions (%)
**275**	0.129	α-HOSO-4 → α-LUSO+6 (26)<br>α-HOSO-3 → α-LUSO+7 (40)<br>α-HOSO-3 → α-LUSO+10 (27)<br>β-HOSO-6 → β-LUSO+6 (32)<br>β-HOSO-4 → β-LUSO+6 (43)<br>β-HOSO-2 → β-LUSO+9 (21)
**258**	0.136	α-HOSO-12 → α-LUSO+3 (27)<br>α-HOSO-4 → α-LUSO+8 (51)<br>β-HOSO-2 → β-LUSO+10 (22)<br>β-HOSO-2 → β-LUSO+11 (30)<br>β-HOSO → β-LUSO+12 (21)
**241**	0.105	β HOSO-17 → β-LUSO (70)<br>β-HOSO-3 → β-LUSO+10 (32)
**222**	0.073	α-HOSO-9 → α-LUSO+4 (28)<br>α-HOSO-6 → α-LUSO+8 (31)<br>α-HOSO-2 → α-LUSO+31 (21)<br>β-HOSO-3 → β-LUSO+11 (27)<br>β-HOSO-3 → β-LUSO+12 (34)
**218**	0.067	α-HOSO-12 → α-LUSO+4 (44)<br>α-HOSO-2 → α-LUSO+31 (36)<br>β-HOSO-4 → β-LUSO+8 (21)

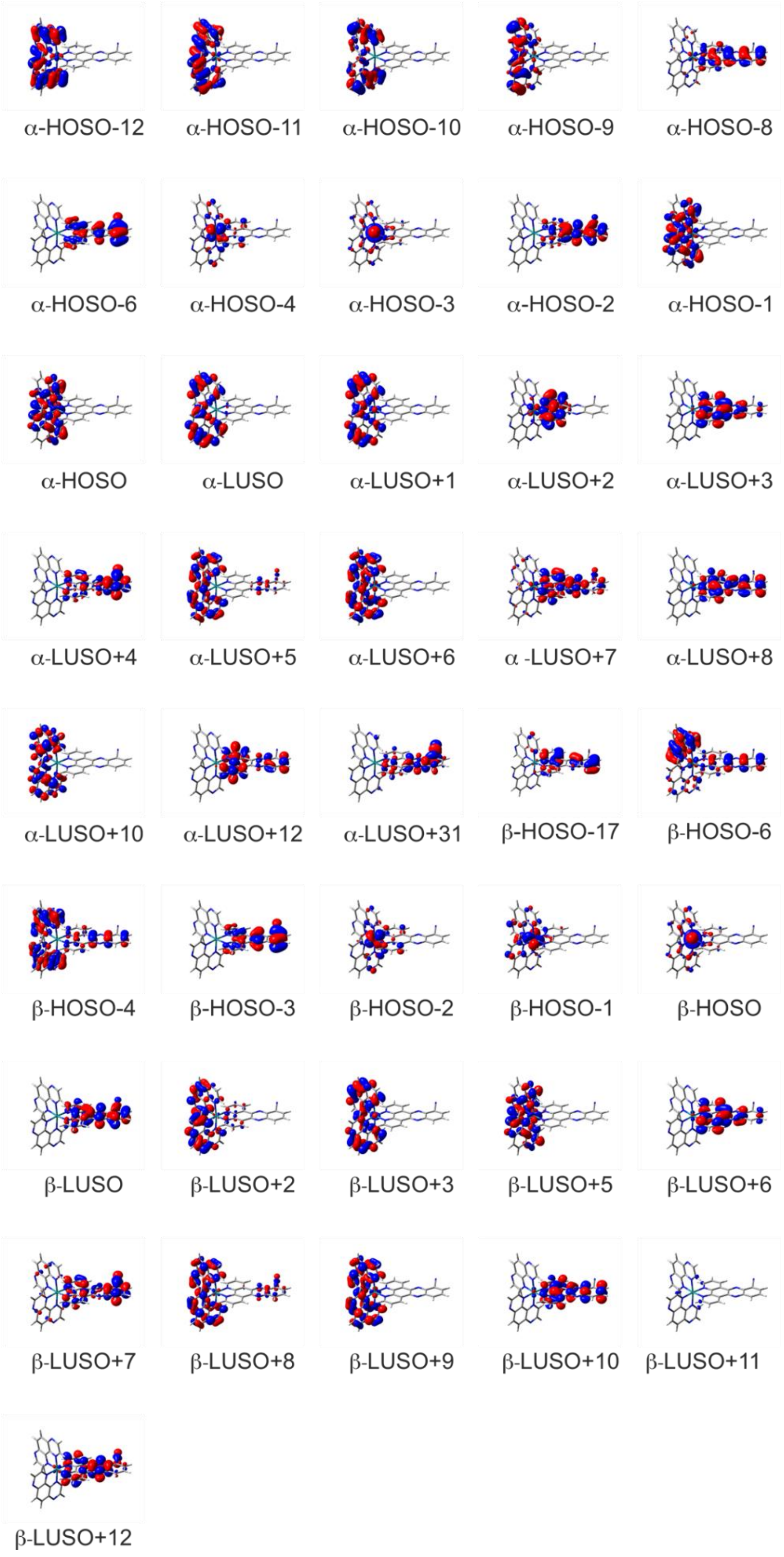

Figure S29. MOs involved in the electronic transitions in **1**$^{-}$ (listed in Table S8).

Table S9. Major electronic excitations of 1^{2+} in the lowest ^{3}MLCT state, determined by TD-DFT.

Wavelength (nm)	Osc. strength (*f*)	Major contributions (%)
**1804**	0.018	α-HOSO → α-LUSO+4 (92)
**908**	0.016	β-HOSO-3 → β-LUSO (25)<br>β-HOSO-2 → β-LUSO (31)<br>β-HOSO-1 → β-LUSO (80)<br>β-HOSO → β-LUSO (29)
**827**	0.005	α-HOSO → α-LUSO+6 (95)
**772**	0.005	α-HOSO → α-LUSO+7 (22)<br>α-HOSO → α-LUSO+8 (94)
**557**	0.013	α-HOSO → α-LUSO+7 (24)<br>α-HOSO → α-LUSO+9 (41)<br>α-HOSO → α-LUSO+11 (22)<br>α-HOSO → α-LUSO+12 (78)<br>α-HOSO → α-LUSO+13 (24)
**540**	0.010	β-HOSO-10 → β-LUSO (72)<br>β-HOSO-9 → β-LUSO (55)<br>β-HOSO-2 → β-LUSO (23)
**471**	0.025	α-HOSO → α-LUSO+10 (56)<br>α-HOSO → α-LUSO+11 (42)<br>α-HOSO → α-LUSO+12 (21)<br>α-HOSO → α-LUSO+14 (62)
**460**	0.021	α-HOSO → α-LUSO+10 (49)<br>α-HOSO → α-LUSO+11 (43)<br>α-HOSO → α-LUSO+14 (70)
**378**	0.042	α-HOSO-4 → α-LUSO+3 (31)<br>β-HOSO-17 → β-LUSO (21)<br>β-HOSO-14 → β-LUSO (60)<br>β-HOSO-3 → β-LUSO+4 (38)<br>β-HOSO → β-LUSO+4 (21)
**376**	0.054	α-HOSO-1 → α-LUSO+1 (64)<br>β-HOSO-2 → β-LUSO+2 (57)<br>β-HOSO-1 → β-LUSO+2 (20)<br>β-HOSO → β-LUSO+2 (22)
**364**	0.055	α-HOSO-3 → α-LUSO+4 (22)<br>β-HOSO-17 → β-LUSO (32)<br>β-HOSO-15 → β-LUSO (32)<br>β-HOSO-2 → β-LUSO+2 (27)<br>β-HOSO-1 → β-LUSO+2 (33)<br>β-HOSO-1 → β-LUSO+7 (20)<br>β-HOSO → β-LUSO+2 (49)
**352**	0.230	α-HOSO-4 → α-LUSO+1 (39)<br>α-HOSO-3 → α-LUSO (28)<br>α-HOSO-2 → α-LUSO+1 (28)<br>α-HOSO-1 → α-LUSO+5 (22)<br>β-HOSO-3 → β-LUSO+2 (55)<br>β-HOSO → β-LUSO+2 (24)

Wavelength (nm)	Osc. strength (*f*)	Major contributions (%)
**299**	0.201	α-HOSO-12 → α-LUSO (22)<br>α-HOSO-9 → α-LUSO+5 (25)<br>α-HOSO-8 → α-LUSO+1 (39)<br>β-HOSO-10 → β-LUSO+1 (25)<br>β-HOSO-2 → β-LUSO+6 (29)<br>β-HOSO-1 → β-LUSO+6 (22)
**297**	0.185	α-HOSO-9 → α-LUSO+5 (32)<br>α-HOSO-1 → α-LUSO+5 (37)<br>β-HOSO-9 → β-LUSO+6 (20)<br>β-HOSO-8 → β-LUSO+3 (25)<br>β-HOSO-4 → β-LUSO+4 (26)<br>β-HOSO-2 → β-LUSO+6 (25)
**292**	0.317	α-HOSO-11 → α-LUSO+1 (29)<br>α-HOSO-10 → α-LUSO+1 (40)<br>β-HOSO-25 → β-LUSO (21)<br>β-HOSO-20 → β-LUSO (30)<br>β-HOSO-9 → β-LUSO+2 (23)
**285**	0.123	α-HOSO-13 → α-LUSO+2 (22)<br>α-HOSO-9 → α-LUSO+2 (27)<br>β-HOSO-24 → β-LUSO (29)<br>β-HOSO-23 → β-LUSO (23)<br>β-HOSO-11 → β-LUSO+1 (20)
**280**	0.160	α-HOSO-12 → α-LUSO+1 (38)<br>α-HOSO-12 → α-LUSO+2 (28)<br>α-HOSO-10 → α-LUSO+1 (22)<br>α-HOSO-9 → α-LUSO+2 (22)<br>α-HOSO-8 → α-LUSO+2 (22)<br>β-HOSO-11 → β-LUSO+1 (21)<br>β-HOSO-11 → β-LUSO+2 (25)
**272**	0.168	α-HOSO-12 → α-LUSO+1 (20)<br>α-HOSO-12 → α-LUSO+3 (37)<br>α-HOSO-9 → α-LUSO+3 (27)<br>α-HOSO-4 → α-LUSO+5 (38)<br>β-HOSO-10 → β-LUSO+4 (32)
**266**	0.123	α-HOSO-12 → α-LUSO+3 (31)<br>α-HOSO-9 → α-LUSO+3 (21)<br>β-HOSO-29 → β-LUSO (22)<br>β-HOSO-28 → β-LUSO (27)<br>β-HOSO-3 → β-LUSO+6 (20)
**234**	0.130	α-HOSO-17 → α-LUSO+1 (37)<br>α-HOSO-11 → α-LUSO+6 (20)<br>α-HOSO-8 → α-LUSO+6 (42)<br>α-HOSO-1 → α-LUSO+10 (22)<br>β-HOSO-15 → β-LUSO+2 (26)<br>β-HOSO-2 → β-LUSO+12 (20)

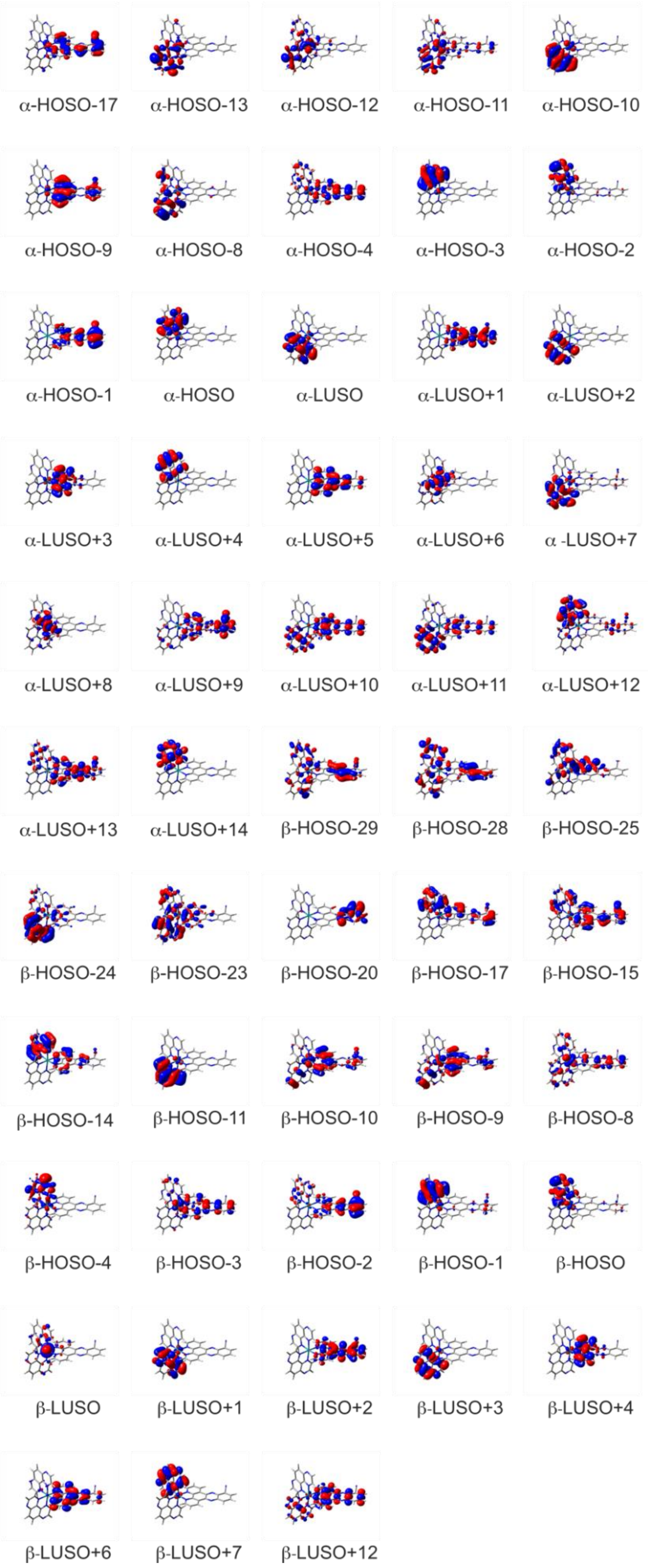

Figure S30. MOs involved in the electronic transitions of **1**$^{2+}$ in the lowest ^{3}MLCT excited state (listed in Table S9).

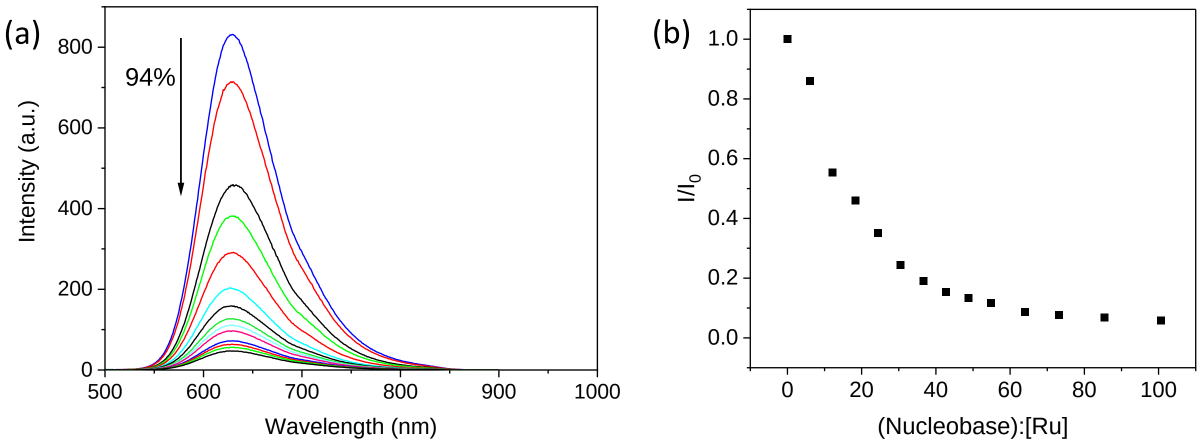

Figure S31. (a) Visible-range emission spectrum of *rac*-[**1**$^{2+}$]Cl_2 (31.5 μM) (λ_{exc} = 450 nm) titrated against increasing concentrations of GMP (0 → 3.13 mM nucl.) in 50 mM potassium phosphate buffer, *T* = 298 K, pH 7.

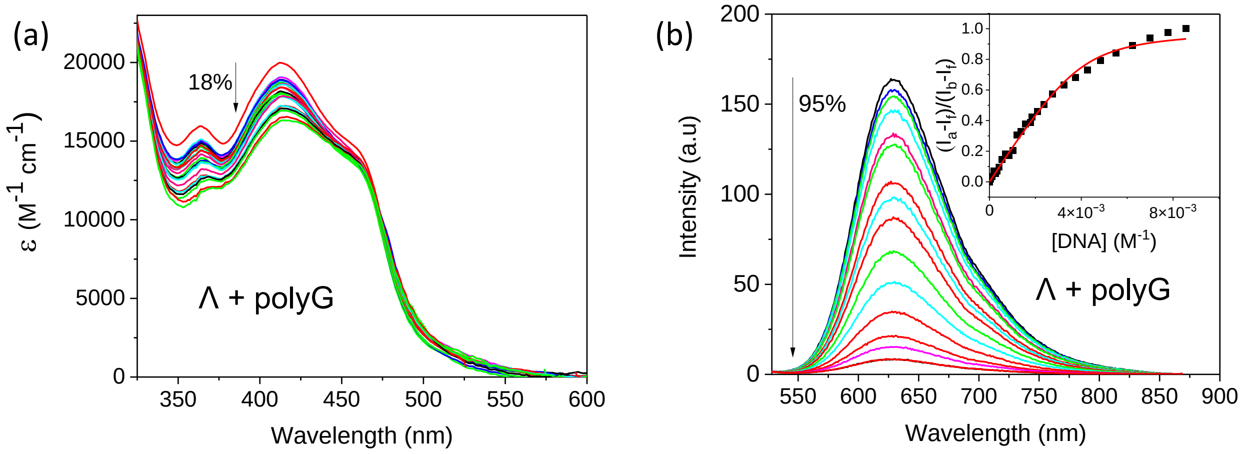

Figure S32. (a) Visible absorption and (b) emission (λ_{exc} = 450 nm) spectra of Λ-[**1**$^{2+}$]Cl_2 (21.4 μM) titrated against increasing concentrations of polyG (0 → 13.8 mM nucl.) in 50 mM potassium phosphate buffer.

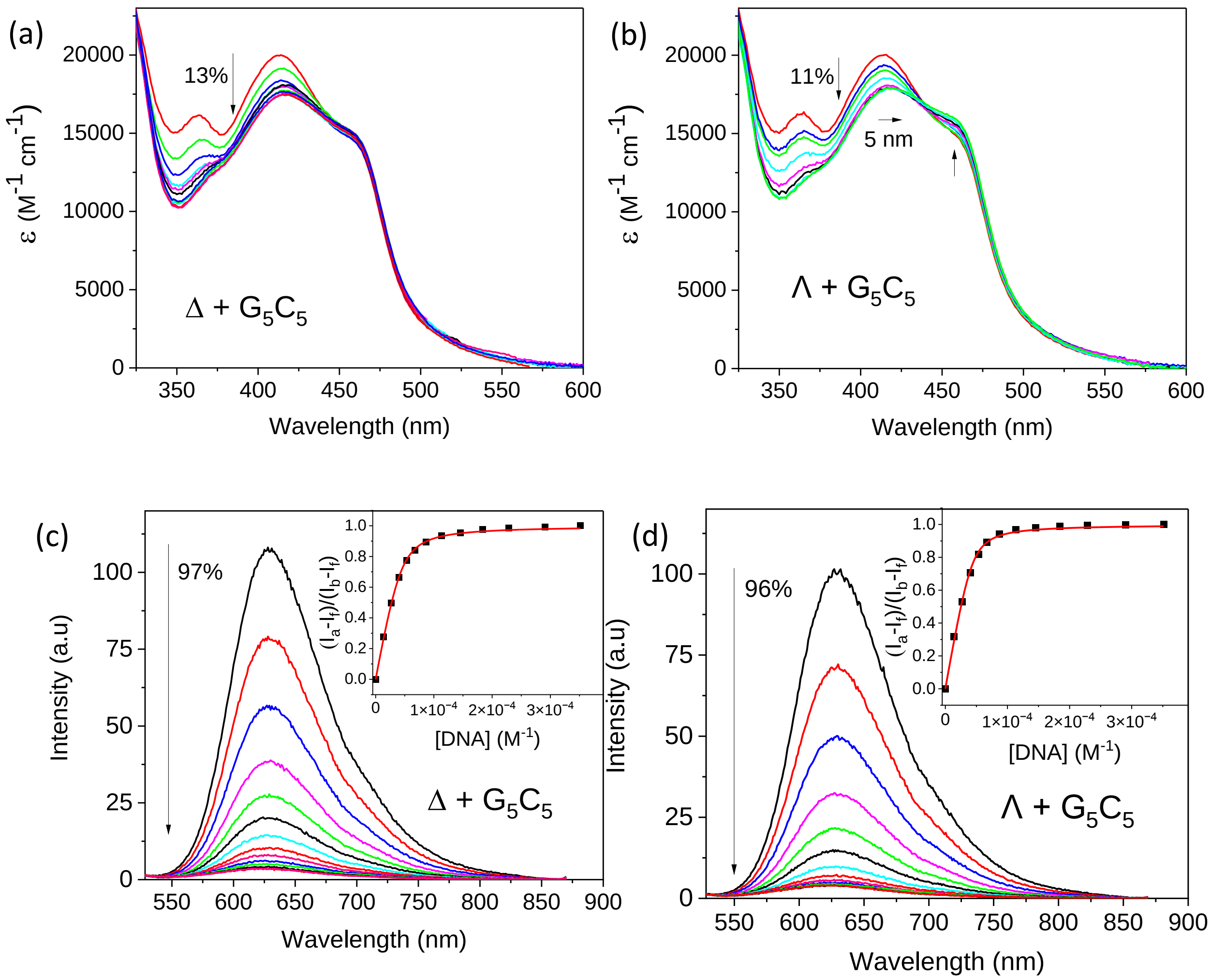

Figure S33. (a-b) Visible absorption and (c-d) emission (λ_{exc} = 450 nm) spectra of Λ-[**1**$^{2+}$]Cl_2 and Δ-[**1**$^{2+}$]Cl_2 (21.4 μM) titrated against increasing concentrations of G_5C_5 (0 → 91 μM duplex) in 50 mM potassium phosphate buffer.

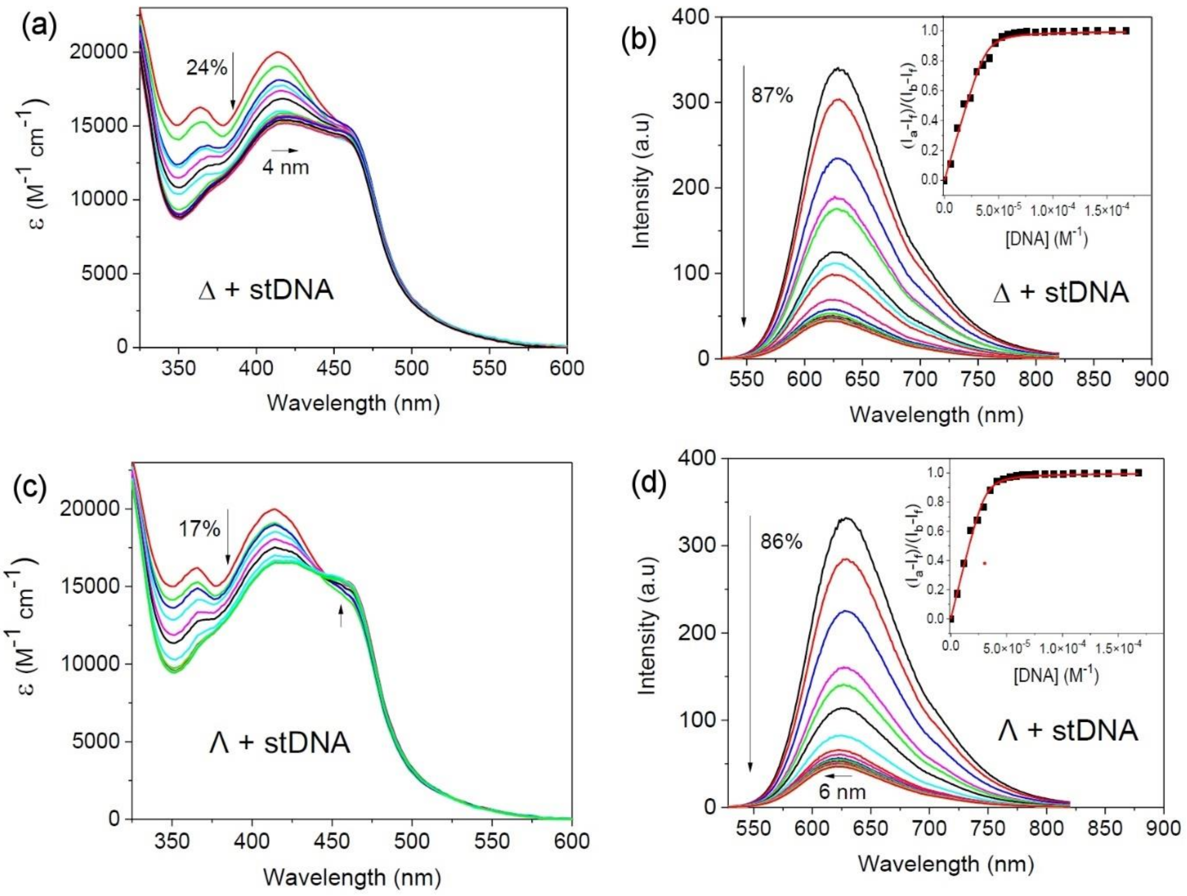

Figure S34. (a, c) Visible absorption and (b, d) emission (λ_{exc} = 450 nm) spectra of Δ-[**1**$^{2+}$]Cl_2 and Λ-[**1**$^{2+}$]Cl_2 (21.4 μM) titrated against increasing concentrations of st-DNA (0 → 1.8 mM nucl) in 50 mM potassium phosphate buffer.

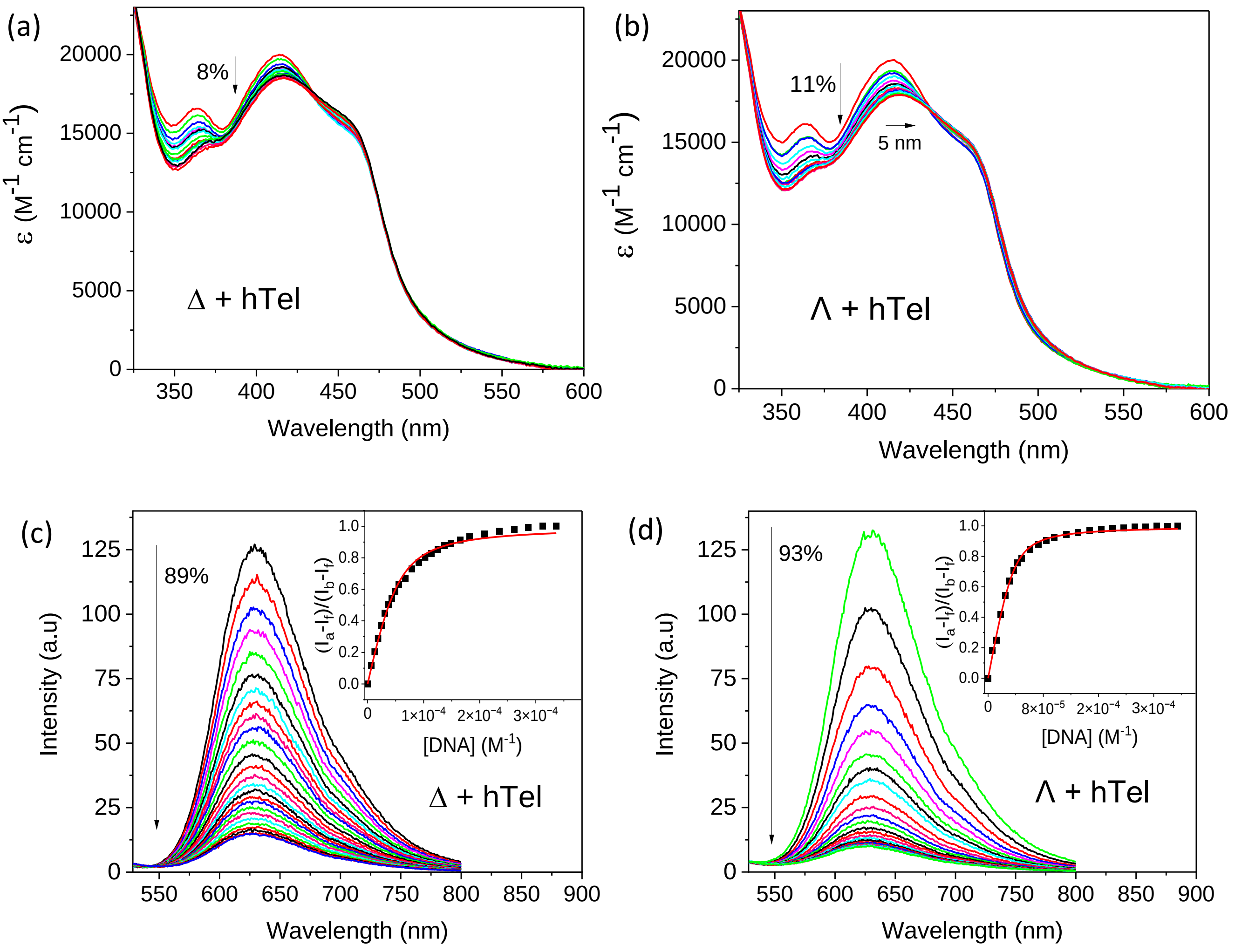

Figure S35. (a-b) Visible absorption and (c-d) emission (λ_{exc} = 450 nm) spectra of Δ-[**1**$^{2+}$]Cl_2 and Λ-[**1**$^{2+}$]Cl_2 (21.4 μM) titrated against increasing concentrations of **hTel** (0 → 150 μM htel) in 50 mM potassium phosphate buffer and 100 mM KCl.

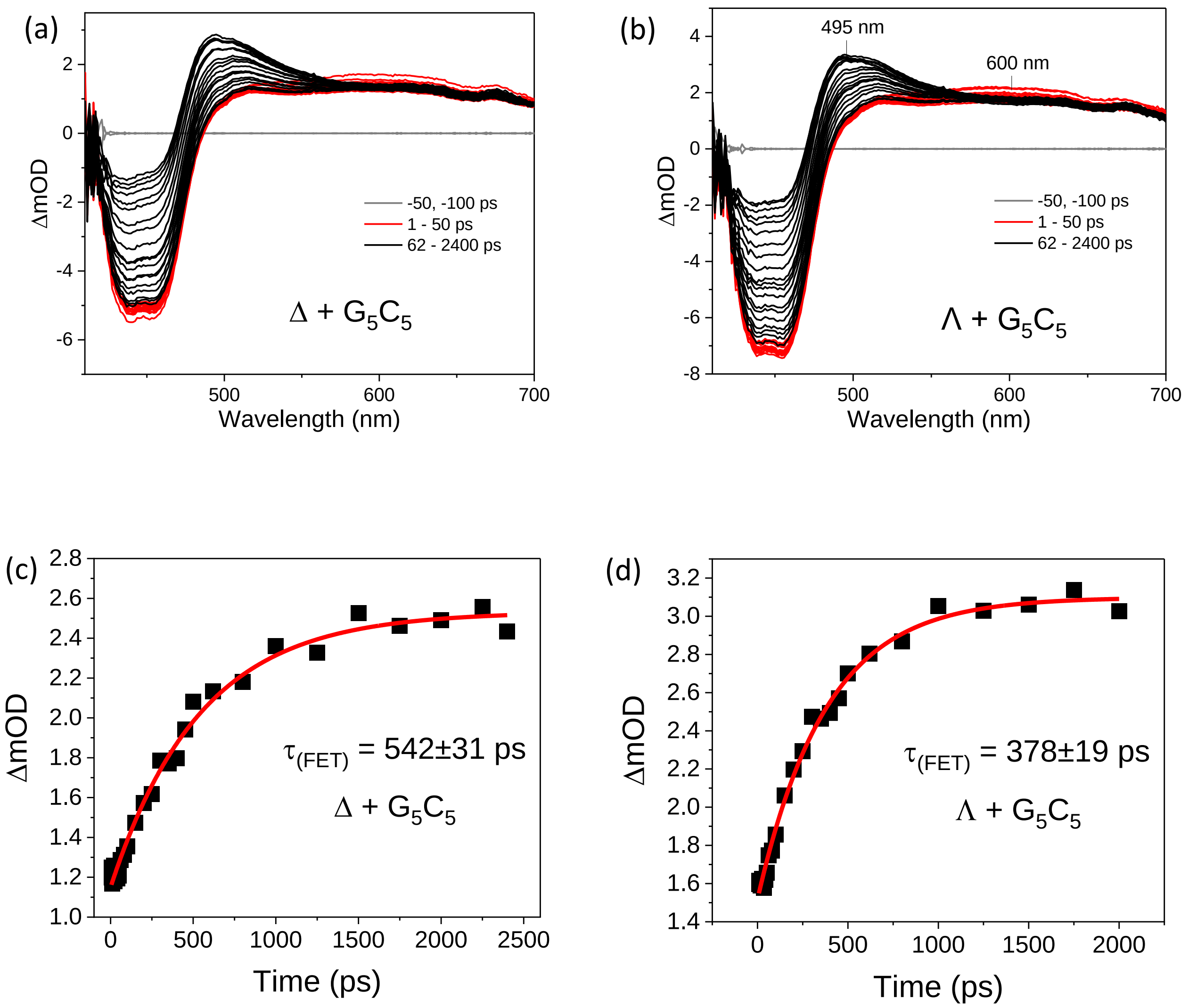

Figure S36. TrA spectroscopy of (a) Λ-[**1**$^{2+}$]Cl_2 (50 µM) and (b) Δ-[**1**$^{2+}$]Cl_2 (50 µM) in the presence of 2 mM DNA (per nucleobase) in 50 mM phosphate buffer. The absorption band maxima of the complex in the lowest ^{3}MLCT excited state (600 nm) and the singly reduced complex (505 nm) are also included. Fitted kinetics (c-d).

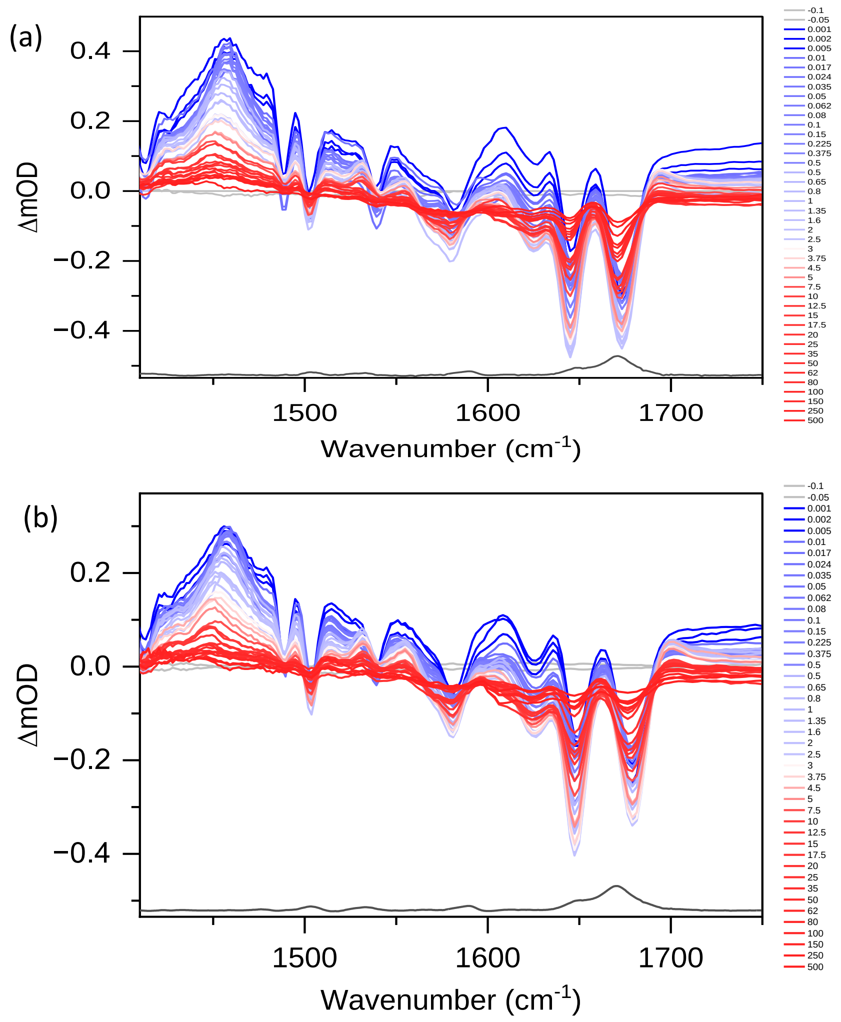

Figure S37. TRIR difference-absorbance spectra of 0.4 mM of (a) Λ-[**1**$^{2+}$]Cl$_2$ and (b) Δ-[**1**$^{2+}$]Cl$_2$ in the 0.5 mM (per duplex**) G$_5$C$_5$** DNA in 50 mM K-phosphate, pH 7, in D_2O (λ_{exc} = 400 nm, 2 kHz,150 fs).

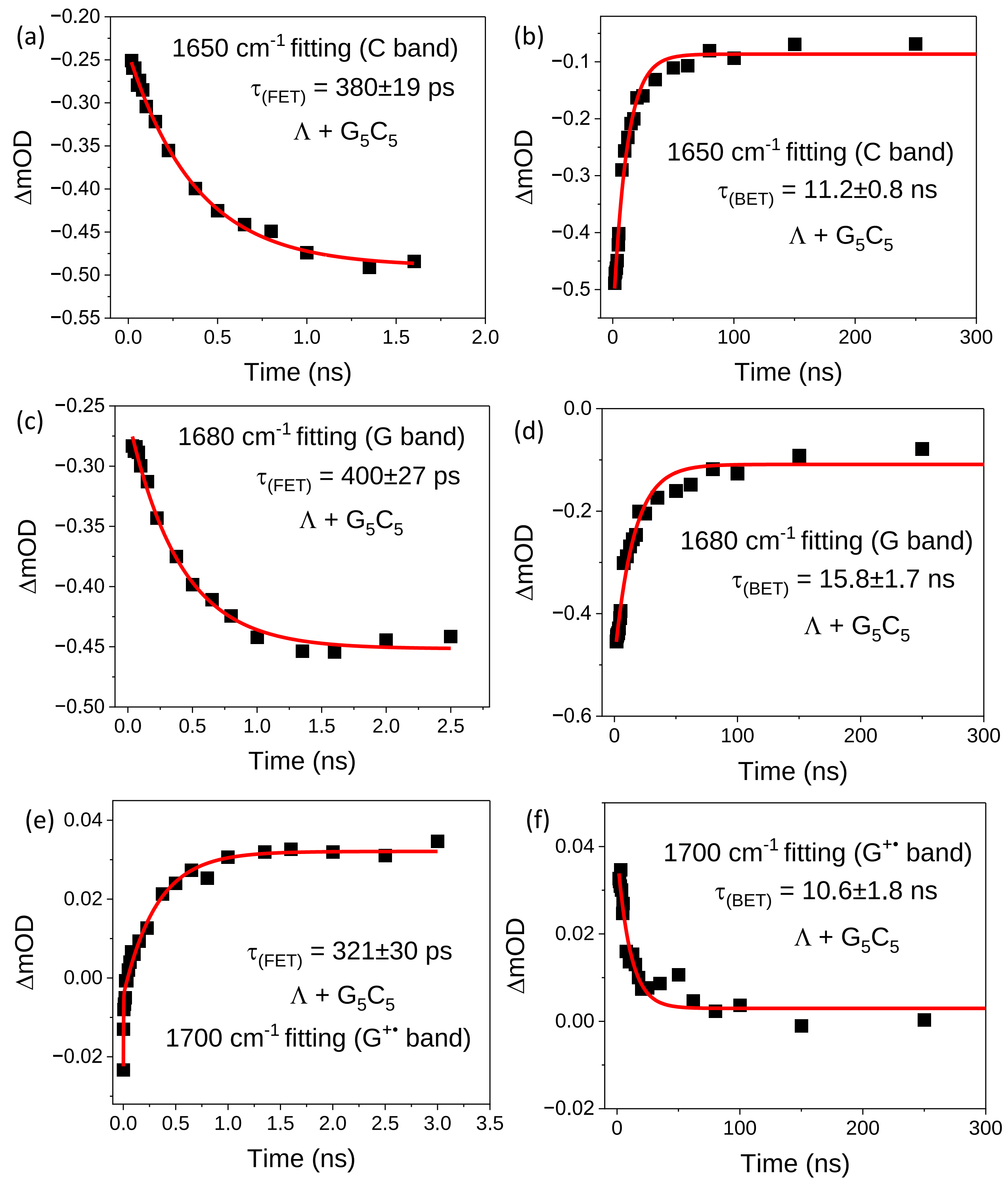

Figure S38. (a-f) TRIR kinetic plots for Λ-[**1**$^{2+}$]Cl_2 (0.4 mM) in the presence of (0.5 mM) (per duplex) **G_5C_5** in 50 mM phosphate buffer.

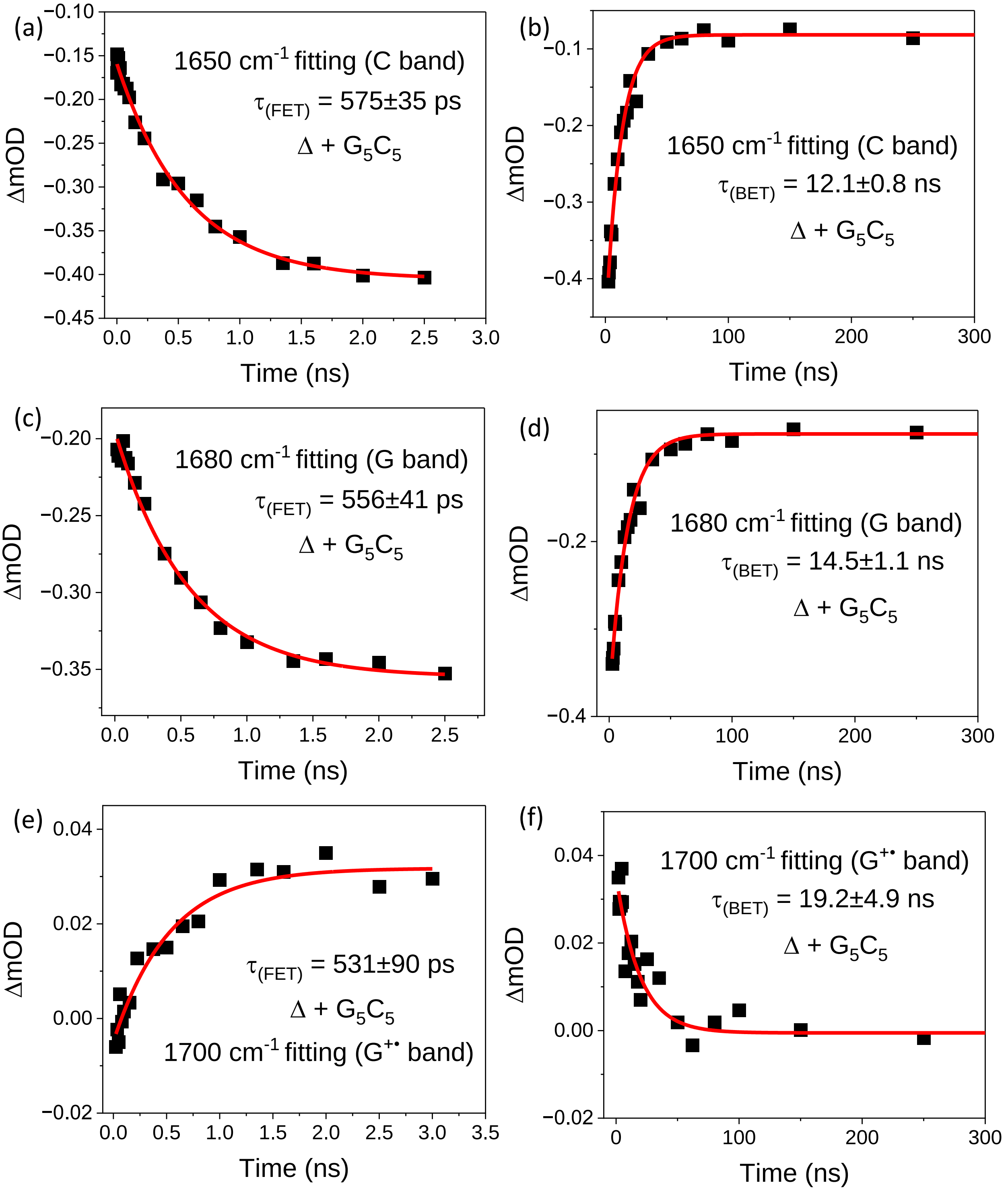

Figure S39. (a-f) TRIR kinetic plots for Δ-[**1**$^{2+}$]Cl$_2$ (0.4 mM) in the presence of (0.5 mM) (per duplex) **G$_5$C$_5$** in 50 mM phosphate buffer.

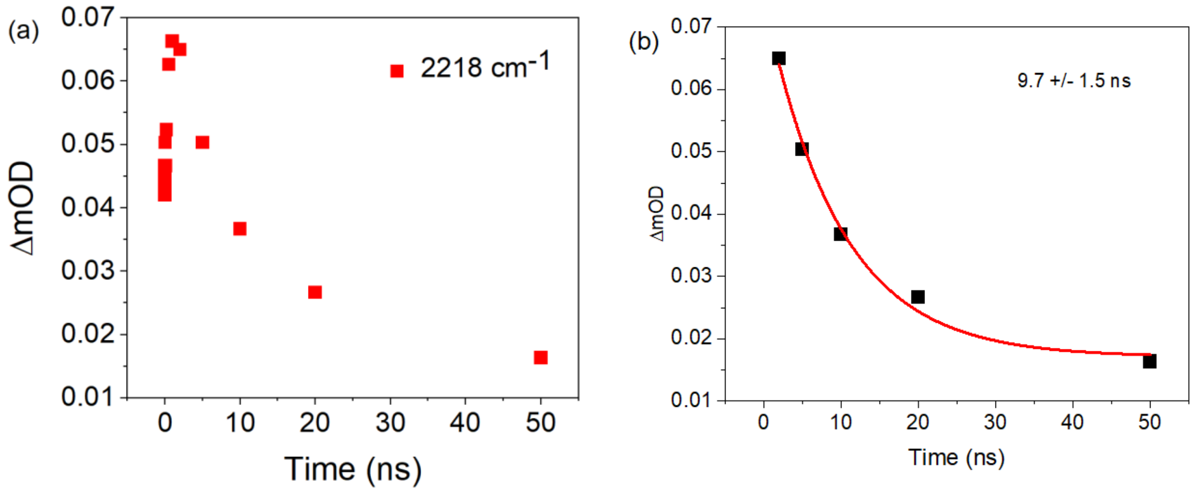

Figure S40: (a) overview of the FET and BET monitored at 2218 cm^{-1} for 0.4 mM of Λ-**1**$^{2+}$ in the presence of 0.5 mM (per duplex) G_5C_5 DNA in 50 mM phosphate buffer in D_2O, pH 7 (λ_{exc} = 400 nm, 2 kHz,150 fs). (b) Extracted kinetic analysis of the BET TRIR-monitored at 2218 cm^{-1}.

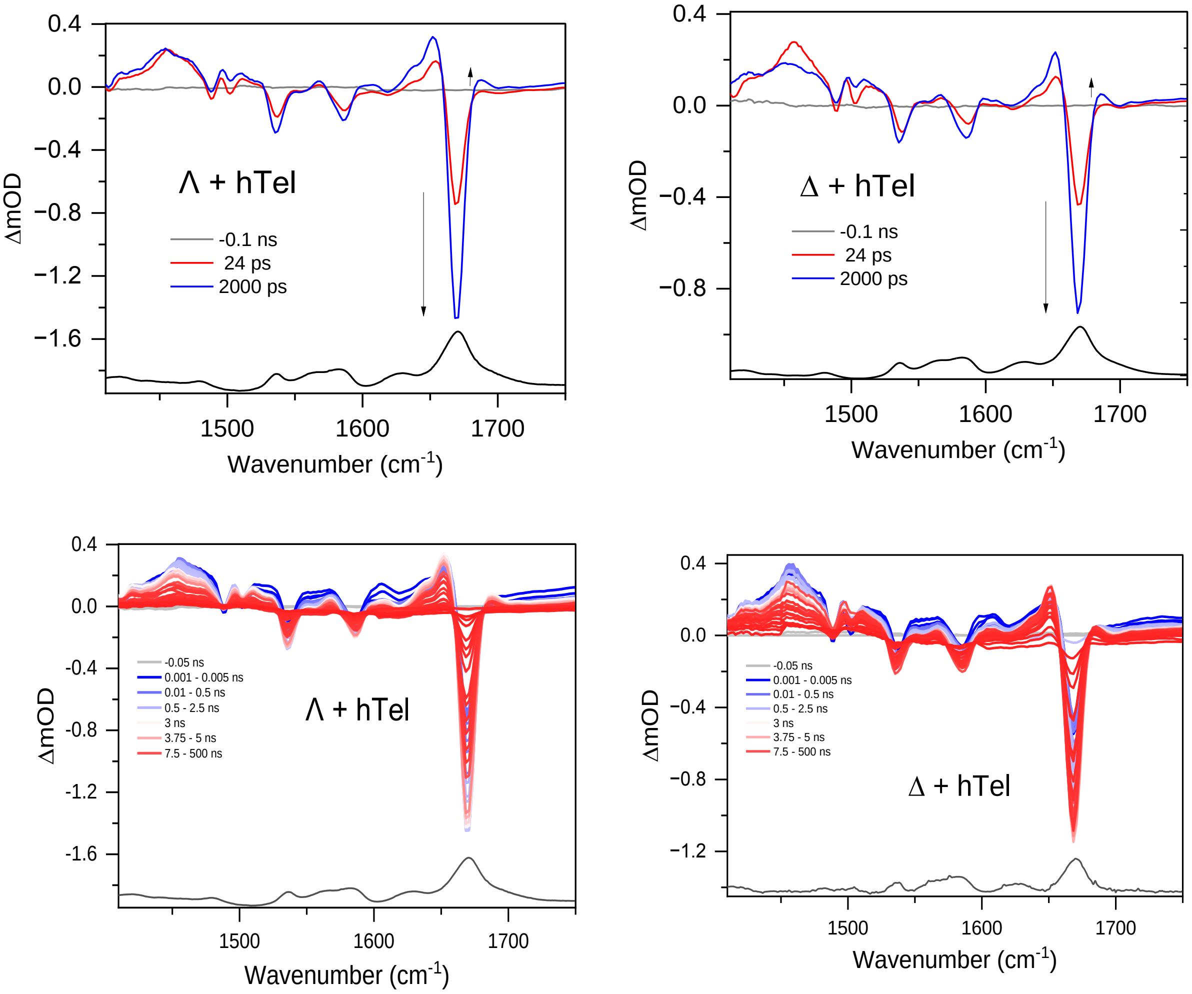

Figure S41. Difference-absorbance TRIR spectra of 0.4 mM of (a) Λ-[**1**$^{2+}$]Cl_2 and (b) Δ -[**1**$^{2+}$]Cl_2 in the presence of 1.2 mM of **hTel** (per G4) in 50 mM K-phosphate and 100 mM KCl, pH 7, in D_2O (λ_{exc} = 400 nm, 2 kHz,150 fs).

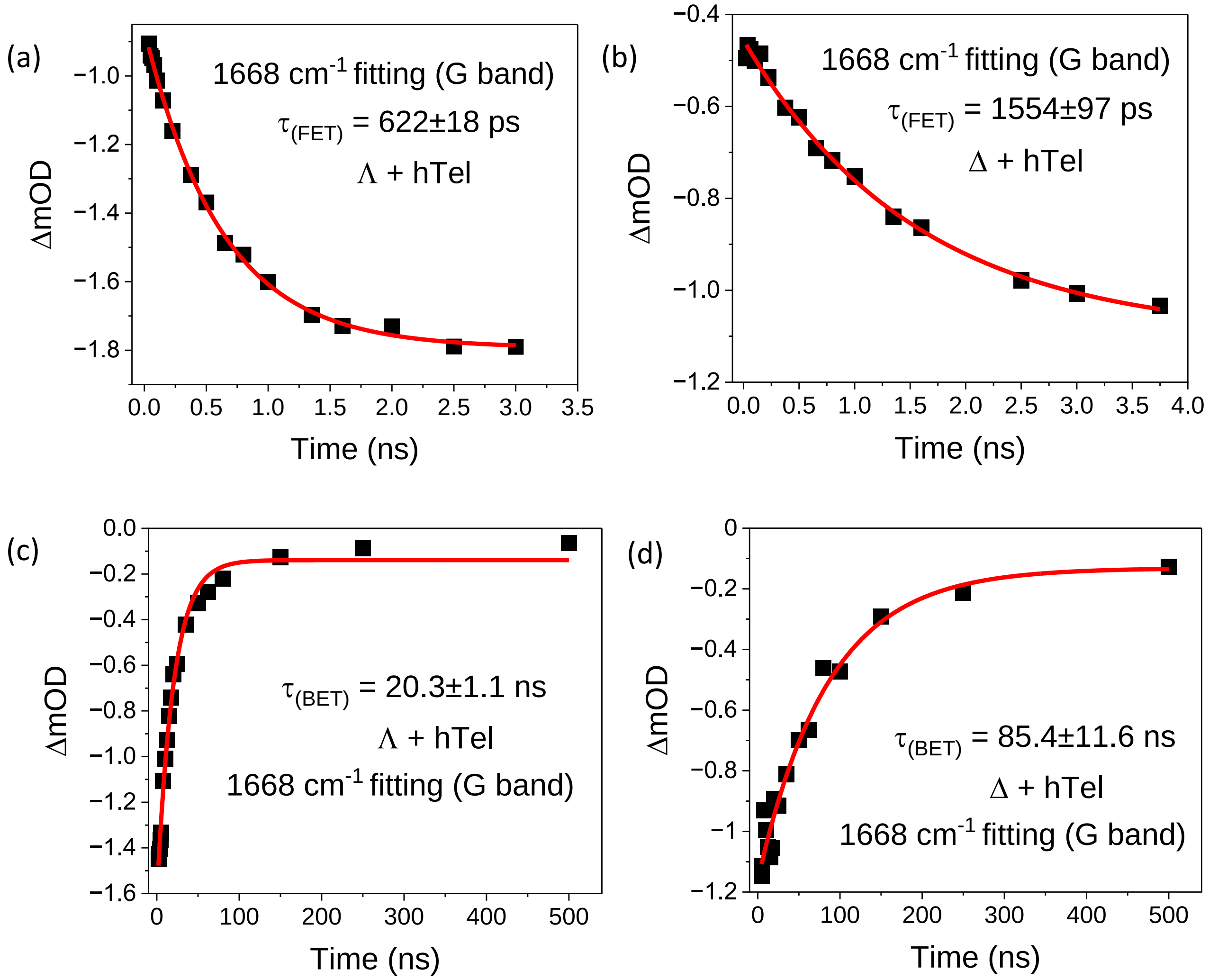

Figure S42. (a-d) TRIR kinetic plots of Λ-[**1**$^{2+}$]Cl$_2$ and Δ-[**1**$^{2+}$]Cl$_2$ (0.4 mM) in the presence of 1.2 mM **hTel** (per G4) in 50 mM phosphate buffer and 100 mM KCl.

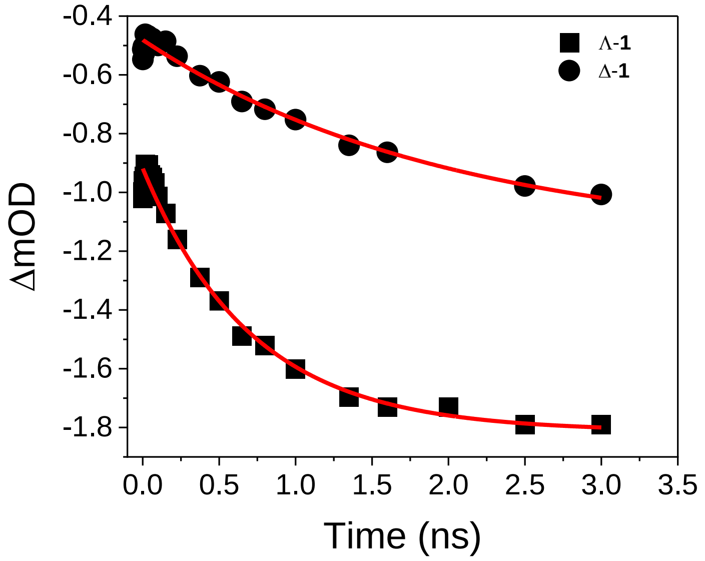

Figure S43. Comparative TRIR kinetic analysis of the FET (TRIR-monitored at 1668 cm^{-1}) of Λ-[**1**$^{2+}$]Cl_2 and Δ-[**1**$^{2+}$]Cl_2 (0.4 mM) in the presence of 1.2 mM **hTel** (per G4) in 50 mM phosphate buffer and 100 mM KCl.

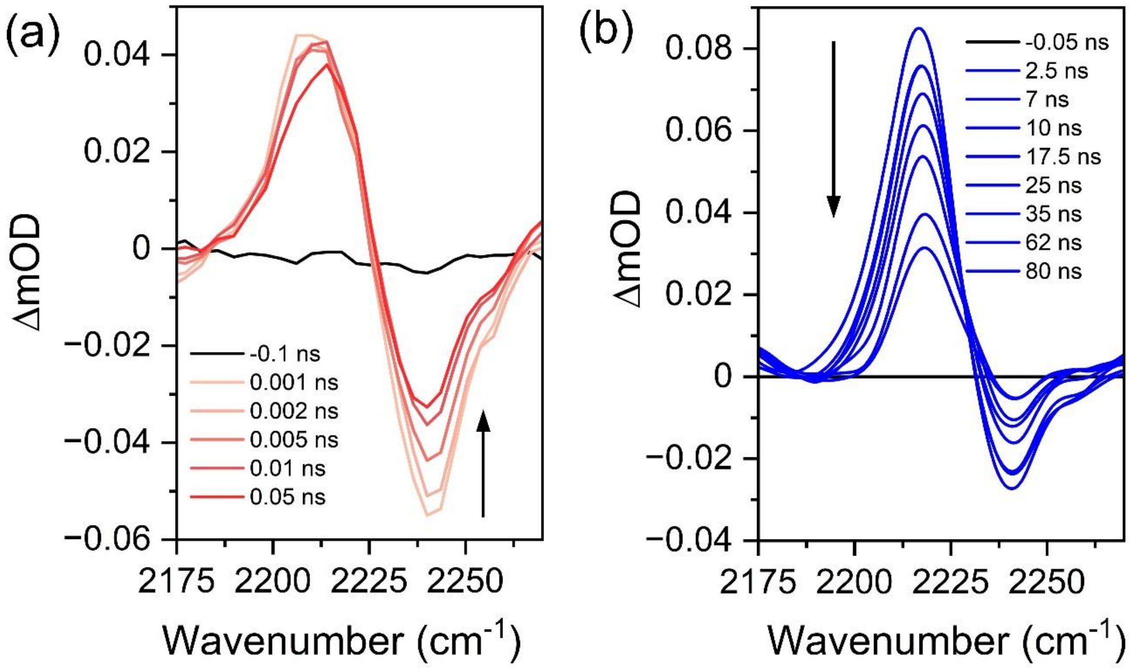

Figure 44. (a) Difference-absorbance TRIR spectra of 0.8 mM Λ-[**1**$^{2+}$]Cl_2 in the presence of 2.4 mM **hTel** (per G4) in 50 mM potassium phosphate and 100 mM KCl, pH 7, in H_2O (λ_{exc} = 400 nm, 2 kHz,150 fs). (a) 0–50 ps and (b) 2.5 ns to 80 ns.

References

1. Krejčik, M.; Daněk, M.; Hartl, F., Simple construction of an infrared optically transparent thin-layer electrochemical cell: Applications to the redox reactions of ferrocene, Mn2(CO)10 and Mn(CO)3(3,5-di-t-butyl-catecholate). *J. Electroanal. Chem. and Interf. Electrochem.* **1991,** *317*, 179-187.
2. Frisch, M. J.; Trucks, G. W.; Schlegel, H. B.; Scuseria, G. E.; Robb, M. A.; Cheeseman, J. R.; Scalmani, G.; Barone, V.; Petersson, G. A.; Nakatsuji, H.; Li, X.; Caricato, M.; Marenich, A. V.; Bloino, J.; Janesko, B. G.; Gomperts, R.; Mennucci, B.; Hratchian, H. P.; Ortiz, J. V.; Izmaylov, A. F.; Sonnenberg, J. L.; Williams; Ding, F.; Lipparini, F.; Egidi, F.; Goings, J.; Peng, B.; Petrone, A.; Henderson, T.; Ranasinghe, D.; Zakrzewski, V. G.; Gao, J.; Rega, N.; Zheng, G.; Liang, W.; Hada, M.; Ehara, M.; Toyota, K.; Fukuda, R.; Hasegawa, J.; Ishida, M.; Nakajima, T.; Honda, Y.; Kitao, O.; Nakai, H.; Vreven, T.; Throssell, K.; Montgomery Jr., J. A.; Peralta, J. E.; Ogliaro, F.; Bearpark, M. J.; Heyd, J. J.; Brothers, E. N.; Kudin, K. N.; Staroverov, V. N.; Keith, T. A.; Kobayashi, R.; Normand, J.; Raghavachari, K.; Rendell, A. P.; Burant, J. C.; Iyengar, S. S.; Tomasi, J.; Cossi, M.; Millam, J. M.; Klene, M.; Adamo, C.; Cammi, R.; Ochterski, J. W.; Martin, R. L.; Morokuma, K.; Farkas, O.; Foresman, J. B.; Fox, D. J. *Gaussian 16 Rev. C.01*, Wallingford, CT, 2016.
3. Becke, A. D., Density-functional thermochemistry. III. The role of exact exchange. *J. Chem. Phys.* **1993,** *98*, 5648-5652.
4. Lee, C. T.; Yang, W. T.; Parr, R. G., Development of the Colle-Salvetti correlation-energy formula into a functional of the electron-density *Phys. Rev. B: Condens. Matter.* **1988,** *37*, 785-789.
5. Andrae, D.; Häussermann, U.; Dolg, M.; Stoll, H.; Preuss, H., Energy-adjusted ab initio pseudopotentials for the second and third row transition elements. *Theor. Chim. Acta* **1990,** *77*, 123-141.
6. Krishnan, R.; Binkley, J. S.; Seeger, R.; Pople, J. A., Self-consistent molecular orbital methods. XX. A basis set for correlated wave functions. *J. Chem. Phys.* **1980,** *72*, 650-654.
7. Cossi, M.; Rega, N.; Scalmani, G.; Barone, V., Energies, Structures, and Electronic Properties of Molecules in Solution with the C-PCM Solvation Model. *J. Comput. Chem.* **2003,** *24*, 669-681.
8. Vlček, A., Jr.; Záliš, S., Modeling of Charge Transfer Transitions and Excited States in d6 Transition Metal Complexes by DFT Techniques. *Coord. Chem. Rev.* **2007,** *251*, 258-287.
9. Greetham, G. M.; Donaldson, P. M.; Nation, C.; Sazanovich, I. V.; Clark, I. P.; Shaw, D. J.; Parker, A. W.; Towrie, M., A 100 kHz Time-Resolved Multiple-Probe Femtosecond to Second Infrared Absorption Spectrometer. *Appl. Spectrosc.* **2016,** *70*, 645-653.
10. Greetham, G. M.; Burgos, P.; Cao, Q.; Clark, I. P.; Codd, P. S.; Farrow, R. C.; George, M. W.; Kogimtzis, M.; Matousek, P.; Parker, A. W.; Pollard, M. R.; Robinson, D. A.; Xin, Z.-J.; Towrie, M., ULTRA: A Unique Instrument for Time-Resolved Spectroscopy. *Appl. Spectrosc.* **2010,** *64*, 1311-1319.
11. Shinde, S. S.; Maroz, A.; Hay, M. P.; Anderson, R. F., One-Electron Reduction Potential of the Neutral Guanyl Radical in the GC Base Pair of Duplex DNA. *J.Phys.Chem.* **1996,** *100*, 5541-5553.
12. Aranzaes, J. R.; Daniel, M.-C.; Astruc, D., Metallocenes as references for the determination of redox potentials by cyclic voltammetry: Permethylated iron and cobalt sandwich complexes, inhibition by polyamine dendrimers, and the role of hydroxy-containing ferrocenes. *Can. J. Chem.* **2006,** *84*, 288-299.
13. Sawyer, D. T. S., Andrzej; Roberts, Julian L., *Electrochemistry for Chemists, 2nd Ed*.; Wiley: 1995.
